# Cellular therapy for myocardial ischemia using a temperature-responsive biodegradable injectable polymer system with adipose-derived stem cells

**DOI:** 10.1080/14686996.2021.1938212

**Published:** 2021-08-06

**Authors:** Yuta Yoshizaki, Hiroki Takai, Nozomi Mayumi, Soichiro Fujiwara, Akinori Kuzuya, Yuichi Ohya

**Affiliations:** a Organization for Research and Development of Innovative Science and Technology (ORDIST), Kansai University, Suita, OsakaJapan; b Faculty of Chemistry, Materials, Bioengineering, Kansai University, Suita, OsakaJapan; c Kansai University Medical Polymer Research Center (KUMP-RC), Kansai University, Suita, Osaka, Japan

**Keywords:** Adipose derived stem cell, biodegradable injectable polymer, cellular therapy, temperature-responsive sol-to-gel transition, myocardial ischemia, paracrine effect, 30 Bio-inspired and biomedical materials; Polymer Materials; Biomaterials; Biomedical application

## Abstract

Adipose-derived stem cell (AdSC) has been attracting attention as a convenient stem cell source. Not only AdSC can differentiate into various tissue cells, but it can also accelerate cell proliferation, anti-inflammation, and angiogenesis by secreting paracrine factors. Studies have demonstrated AdSC treatment of ischemic heart. However, an improvement in the remaining live AdSCs administered at the injected site while maintaining paracrine factor secretion is desired to achieve effective regenerative medicine. We previously reported the ABA-type tri-block copolymer of poly(ɛ-caprolactone-*co*-glycolic acid) and poly(ethylene glycol) (tri-PCG), exhibiting temperature-responsive sol-to-gel transition as biodegradable injectable polymer (IP) systems. Moreover, we recently reported that the biodegradable temperature-triggered chemically cross-linked gelation systems exhibited longer gel state durations using tri-PCG attaching acryloyl groups and a polythiol derivative. In this study, we explored this IP-mediated AdSC delivery system. We investigated the cell viability, mRNA expression, and cytokine secretion of AdSCs cultured in the physical or chemical IP hydrogels. Both of these IP hydrogels retained a certain number of viable cells, and RT-PCR and ELISA analyses revealed that mRNA expression and secretion of vascular endothelial growth factor of the AdSCs cultured in the chemical hydrogel were higher than the physical hydrogel. Moreover, AdSCs injected with the chemical hydrogel into ischemic heart model mice showed longer retention of the cells at the injected site and recovery from the ischemic condition. The results mean that the IP system is a promising candidate for a stem cell delivery system that exhibits the recovery of cardiac function for myocardial infarction treatment.

## Graphical Abstract

**Figure:**
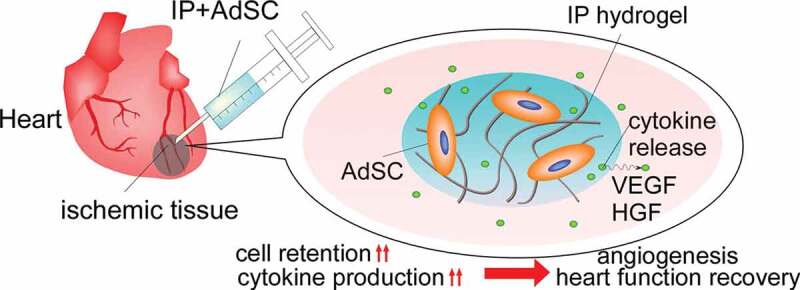

## 1. Introduction

Injectable polymers (IPs) [1–3] are a category of *in situ* gel-forming polymers that occur in a solution (sol) state outside, can be injected by a syringe, and exhibit a sol-to-gel transition after being injected into the body upon mixing of the two solutions via chemical reactions [4–8] or in response to various physical stimuli, such as temperature [9,10], light [11,12], pH [13,14], both temperature and pH [15] etc. IPs are attracting much attention as biomedical materials due to their properties. Among them, temperature-responsive IPs, which show a sol-to-gel transition in response to a temperature increase from room temperature (r.t.) to body temperature, are highly versatile because they can form a hydrogel *in situ* just upon body injection. Besides, materials to be injected into the body should be readily decomposed and absorbed or metabolized after fulfilling their purpose, that is, biodegradable or bioabsorbable.

**Figure 1.**
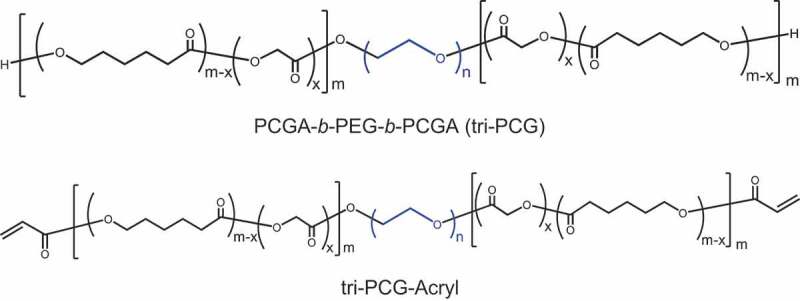
Structure of the polymers used in this study

Recently, we have explored an ABA-type triblock copolymer of poly (ε-caprolactone-*co*-glycolide) (PCGA) and polyethylene glycol (PEG), PCGA-*b*-PEG-*b*-PCGA (tri-PCG) (Figure 1), as a temperature-responsive biodegradable IP and its medical applications [16–18]. Furthermore, we reported on a temperature-responsive covalent cross-link forming biodegradable IP system made by introducing acryloyl groups at both termini of tri-PCG (tri-PCG-Acryl) (Figure 1) by mixing tri-PCG-Acryl micelle solution with tri-PCG micelle solution containing the hydrophobic polythiol (dipentaerythritolhexakis(3-mercaptopropionate) (DPMP)) [19,20]. In this system, upon gelling in response to temperature, the IP can form partial chemically cross-linked networks by bio-orthogonal Michael addition-type thiol-ene reaction between acryloyl groups and thiol groups. We could control the elastic modulus and degradation periods of the obtained hydrogels by a simple method: changing the mixing ratio of tri-PCG-Acryl and tri-PCG/DPMP. We have reported on the potential application of this IP system for sustained-release drug delivery materials [21,22] and postoperative adhesion prevention materials [23].

In recent years, therapeutics using cells as ‘medicines’ have been developed [24,25]. Chimeric antigen receptor-T cells (CAR-T cells) have been marketed as a ‘therapeutic agent’ for hematopoietic tumors [26,27]. Induced pluripotent stem (iPS) cells have been clinically tested as therapeutics for age-related macular degeneration [28]. We reason that embryonic stem (ES) cells or induced pluripotent stem (iPS) cells, which have pluripotency to differentiate into various organs, can be used for the treatment of injuries or diseases that were previously incurable. Although iPS cells and ES cells have been explored as pluripotent stem cells, studies on these are limited; besides, ethical and safety concerns regarding their practical use remain.

Increasing attention has turned to adipose-derived stem cells (AdSCs) due to their convenience as a stem cell source [29,30]. AdSC is a type of mesenchymal stem cell existing in adipose tissue. Similar to bone marrow mesenchymal stem cells, AdSCs can differentiate into osteoblasts, adipocytes, cardiomyocytes, vascular endothelial cells, and so on [31]. Furthermore, AdSC accelerates cell proliferation, anti-inflammation, and angiogenesis by secreting numerous paracrine factors, including cytokines [32–34]. For example, studies have been conducted to treat ischemic heart disease by administering AdSCs [35–37]. Ischemia is a heart disease in which the myocardium is damaged and can be fatal due to a decrease in blood flow in the coronary arteries [38]. A study reported that infusion of AdSCs into mice with myocardial infarction showed recovery of cardiac function, but large doses of AdSCs were required to obtain an effective healing effect [39,40]. To achieve effective therapeutic effects using such cells, IP hydrogel systems containing such cells have been investigated [41–44]. By entrapping these cells in a hydrogel, we expect the possible cell retention at the injected site and the hydrogel function as a scaffold for cell growth and improvement of the cell engraftment efficiency or paracrine effects.

**Figure 2.**
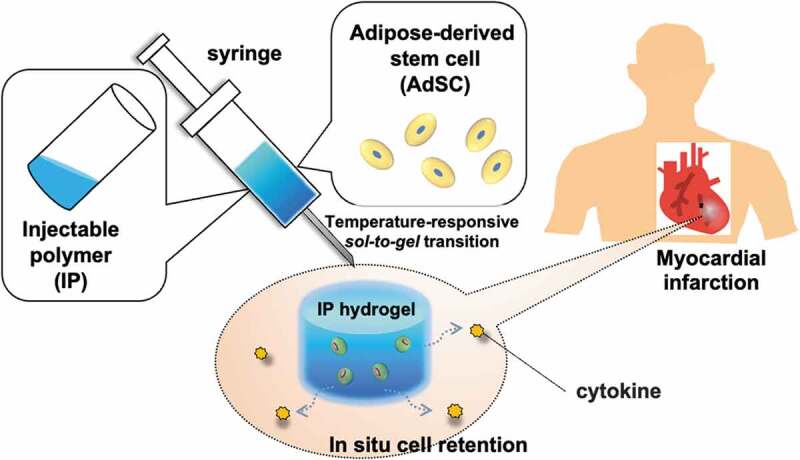
Schematic illustration of the concept of a cellular therapy using adipose-derived stem cells (AdSC) and biodegradable temperature-responsive injectable polymers (IPs) for myocardial infarction

Here, we develop a cell delivery system that can efficiently exert cell functions by mixing AdSCs with our previously developed temperature-responsive biodegradable IP system (Figure 2). Since the IP solution is in a sol state at r.t., AdSCs can be easily mixed and injected into the body to form a hydrogel that entraps the cells. To evaluate the feasibility of the material for cell delivery, we investigated the viability and physiological function of AdSCs retained in the IP hydrogel *in vitro*. Furthermore, the IP formulation with AdSCs was administered to a mice myocardial infarction model, and the therapeutic effects were investigated.

## 2. Materials and Methods

### 2.1. Materials

PEG (*M*_n_ = 1,500 Da), ɛ-caprolactone, tin-2-ethylhexanoate (Sn(Oct)_2_), *N,N*’-dicyclohexylcarbodiimide (DCC), 4-dimethylaminopyridine (DMAP), 5(6)- carboxyfluorescein and the other chemicals for synthesis and organic solvents were purchased from FUJI FILM Wako Pure Chemical Ind., Ltd. (Osaka, Japan). Glycolide, DAPI, and collagenase from *Clostridium histolyticum* were obtained from Sigma–Aldrich Chemical Co. LLC. (St. Louis, MO, USA). DPMP was donated by SC Organic Chemical Co., Ltd. (Osaka, Japan). Eagle’s minimum essential medium (EMEM) and Dulbecco’s modified Eagle medium Nutrient Mixture F-12 (DMEM/F12), were obtained from Nacalai Tesque Inc. (Kyoto, Japan). Media for AdSC (KBM ADSC-1 and KBM ADSC-2) were purchased from Kohjin Bio Co., Ltd. (Saitama, Japan). Fetal bovine serum (FBS) was purchased from Biowest (Nuaillé, France). Water was purified using a Millipore Elix UV3 direct-Q UV (Merck, Darmstadt, Germany). Isoflurane was purchased from Pfizer Inc. (NY, USA). RNeasy Mini Kit was purchased from Qiagen Corp. (Hilden, Germany). PrimeScript^TM^ RT Reagent Kit (Perfect Real Time) was purchased from Takara Bio Inc. (Shiga, Japan). SsoAdvanced^TM^ Universal SYBER Green Mix was purchased from Bio-Rad Laboratories Inc. (Hercules, CA, USA). Hank’s balanced salt solution (HBSS), LIVE/DEAD^TM^ Cell Viability Kit, Cell Tracker Green, and DiI (1,1ʹ-dioctadecyl-3,3,3ʹ,3ʹ-teramethylindocarbocyanine) were obtained from Thermo Fisher Scientific (Waltham, MA, USA).

### 2.2. Measurements

^1^H-Nucleic magnetic resonance (NMR) spectra were obtained using a JNM-ECS400 or GSX-400 spectrometer (JEOL, Tokyo, Japan). Molecular weights of polymers were estimated by ^1^H-NMR or size-exclusion chromatography (SEC) (Tosoh GPC-8020 series system, column: Tosoh TSKgel α-5000, Tosoh Corporation, Tokyo, Japan). Fluorescent images were captured on a confocal laser scanning microscope (CLSM), LSM 800 Axio Observer.Z1 (Carl Zeiss AG, Oberkochen, Germany) using diode lasers at 488 nm and 561 nm. Frozen sections were prepared by LEICA CM1520 cryostat (Leica Biosystems GmbH, Wetzlar, Germany). Cardiac function was sequentially evaluated by echocardiography (Nemio 30; Toshiba Medical Systems, Tochigi, Japan). Real-time PCR was performed on a Bio-Rad CFX96 deep well real-time system (Bio-Rad Laboratories, Inc., Hercules, CA, USA).

### 2.3. Synthesis of tri-PCG and tri-PCG-Acryl

Tri-PCG was synthesized according to the previous report [16] (Scheme S1). Briefly, PEG (*M*_n_ = 1,500, 17.89 g, 11.62 mmol) was placed in a 100 mL pear-shaped flask and dried under reduced pressure in an oil bath for 4 hours at 100°C. Then, ε-Caprolactone (38.57 g, 338.4 mmol), glycolide (6.77 g, 58.4 mmol), and Sn(Oct)_2_ (164.3 mg, 397 μmol) were added into the flask. The content of the flask was frozen by liquid nitrogen and dried under reduced pressure overnight. The obtained solid was melted in an oil bath at 160°C under vacuum to proceed with polymerization reaction for 12 hours. The products were reprecipitated three times using 100 mL of chloroform and 1000 mL of diethyl ether as good and poor solvents, respectively. The obtained yield was 91.5%. Tri-PCG with different segments lengths (tri-PCG-1 and tri-PCG-2) were synthesized by modifying the feeding ratio of glycolide and ɛ-caprolactone to PEG (Table 1).

**Table 1.** Characterization of tri-PCGs and tri-PCG-Acryl

Code	DP of CL ^a,e)^	DP of GA ^b,e)^	CL/GL ^c,e)^	*M*_n_× 10^−3 e)^	*M*_w_/*M*_n_ ^f)^	DS (%) ^d,e)^
tri-PCG-1	14	4.2	3.4	5.3	1.4	-
tri-PCG-2	9.4	2.6	3.7	3.1	1.3	-
tri-PCG-Acryl	9.3	2.6	3.6	3.2	1.3	92

Degree of polymerization of ε-caprolactone in a PCGA segment. Degree of polymerization of glycolic acid in a PCGA segment. Molar ratio of ε-caprolactone to glycolic acid in the polymer. Degree of substitution of acrylic acid per terminal. Estimated by ^1^H-NMR (solvent: CDCl_3_). Estimated by GPC (eluent: DMF, standard: PEG).

The synthesis of tri-PCG-Acryl was also performed according to the previous report [19] (Scheme S2). Acrylic acid (2.0 mL, 29.2 mmol) was weighed in a pear-shaped flask and dissolved in anhydrous CH_2_Cl_2_. Then, DCC (6.1 g, 29.5 mmol) was weighed in a sample tube and dissolved in anhydrous CH_2_Cl_2_. The DCC solution was added to the acrylic acid solution under cooling in an ice bath, and the mixture was stirred for 1 hour. Simultaneously, tri-PCG-2 (20.3 g, 5.1 mmol) and DMAP (299.5 mg, 2.5 mmol) were dissolved in anhydrous CH_2_Cl_2_ in another flask. The solution was added to the acrylic acid/DCC solution and stirred at 25°C for 24 hours. The dicylohexylurea (DCU) was then removed by suction filtration. The filtrate was evaporated, and the products reprecipitated using hexane: ethanol (8:2) mixture (as a poor solvent) three times. The obtained solid was dried under reduced pressure to obtain a slightly yellowish-white solid. The yield and degree of acryloyl group introduction were 67% and 92%, respectively. Characterizations of these polymers are summarized in Table 1.

Fluorescein-labeled tri-PCG was prepared as follows: carboxyfluorescein (0.083 g, 0.22 mmol) and DCC (0.045 g, 0.22 mmol) were dissolved in 1 mL of dimetylformamide (DMF) in a pear-shaped flask and stirred in an ice bath. Tri-PCG (0.981 g, 0.186 mmol) and DMAP (26 μg, 0.22 μmol) dissolved in 3 mL of DMF were then added dropwise to the flask. The mixture was stirred in an ice bath for 2 h, and then at r.t. for 120 h. The DCU and DMF were removed by filtration and under reduced pressure, respectively. The obtained solid was dissolved in chloroform, and reprecipitation was performed four times using diethyl ether/methanol (9/1, v/v) (as a poor solvent) to yield fluorescein-labeled tri-PCG. of 75.5%.

### 2.4. Preparation of IP formulation

IP formulations were prepared as previously described [19]. Briefly, tri-PCG-1 with or without predetermined amounts of DPMP were dissolved in small amounts of acetone. The solution was added dropwise to water with stirring, lyophilized to yield the powdered DPMP-containing tri-PCG. The dried DPMP-containing tri-PCG (total weight: 452.4 mg) weighed in sample tube No. 1. Serum-free medium or phosphate-buffered saline (PBS) (1580 μL) was added to the sample tube with stirring at 25°C. Then, the sample tube was immersed in a warm water bath of approximately 80°C for 5 seconds and stirred at 25°C for approximately 1 minute. This process was performed three times, and bubbles were removed using a bath-type sonicator with ice-cooling. Then, the pH of the obtained solution was adjusted to neutral using 1 M NaOHaq. and serum-free medium to yield DPMP-containing tri-PCG solution (22 wt%). Conversely, 100.1 mg of tri-PCG-Acryl was placed in sample tube No. 2. Serum-free medium or PBS (425 μL) was added to the sample tube, and the mixture was stirred at r.t. for 10 minutes. Bubbles were removed using a bath-type sonicator with ice cooling. Then, the pH of the obtained solution was adjusted to neutral using 1 M NaOHaq. and serum-free medium to yield tri-PCG-Acryl solution (22 wt%). The two solutions in sample tubes No.1 and 2 were mixed in a predetermined ratio at 25°C and vortexed to prepare the IP formulation (total polymer concentration = 22 wt%). By changing mixing ratios of tri-PCG, DPMP, and tri-PCG-Acryl, two different IP formulations, IP(A16) and IP(A25), containing 16 and 25 wt% tri-PCG-Acryl, respectively, in the total polymer (tri-PCG + tri-PCG-Acryl) were prepared. The IP formulations with a total polymer concentration of 25 wt% were also prepared by the same method as above. The final polymer concentration of the IP formulations containing cells was adjusted to 15 wt% or 20 wt% by mixing with cell suspension in medium or dilution with solvents.

### 2.5. Rheological measurements

Temperature-dependent and time course rheological measurements on the temperature-responsive sol-to-gel transition of the IP formulations were performed according to the previous report [19] by using a dynamic rheometer (Thermo HAAKE RS600, Thermo Fisher Scientific, Waltham, MA, USA). A solvent trap was used to prevent solvent vaporization. Each sample was placed between parallel plates (25 mm diameter and 1.0 mm gap) using a syringe. The data was collected under controlled stress (4.0 dyn/cm^2^) and a frequency of 1.0 rad/s. For temperature-dependent measurements, the heating rate was 0.5°C/min. The storage modulus (*G*’) and loss modulus (*G*”) of the formulations ranging 20–50°C were monitored, and the gelation temperature (*T_gel_*) was defined as the cross-over point of *G*’ and *G*”. For post-gelation time course measurements, the temperature was increased from r.t. to 37°C at time 0, and kept at 37°C, while *G*’ and *G*” values were monitored for 60 min.

### 2.6. Animals

C57BL/6 N mice (9 week-old, male) were purchased from SHIMIZU Laboratory Supplies Co., Ltd. (Kyoto, Japan). The experiments were performed for mice under anesthesia with isoflurane using a small animal anesthesia station (DS Pharma Biomedical Co. Ltd., Osaka, Japan). The animal experiments described below followed the guidelines for animal experiments at Kansai University and were approved by the Ethical Committee for Animal Experiments of Kansai University (7 May 2019, Identification number 1903).

### 2.7. Harvesting AdSC

AdSCs were collected as previously reported [36]. Two C57BL/6 N mice (9 week-old, male) were euthanized by cardiac blood sampling under isoflurane anesthesia. Subsequently, the inguinal subcutaneous adipose tissue was collected. The adipose tissue was shredded with scissors and placed in a 15-mL centrifuge tube. Hank’s balanced salt solution (HBSS, 3 mL) containing 1% BSA and collagenase VIII solution (2 mg/mL) was added to the tube at 37°C. The contents of the tube were mixed by inversion, and the tube was then shaken at 37°C for 30 minutes. After removing the uppermost fat layer via aspiration, 3 mL of DMEM/F12 containing 10% FBS was added. The supernatant was passed through a cell strainer (40 μm, BD Bioscience, Franklin Lake, NJ, USA), collected in a centrifuge tube, and removed by centrifugation (250 g, 5 min). After dispersing the pellets in a 1 mL PBS (-) containing 1 mM ethylenediaminetetraacetic acid (EDTA), PBS (-) containing 9 mL of 1 mM EDTA was further added. The supernatant was removed by centrifuging again (250 g, 5 min). Thereafter, the cells were dispersed with medium ADSC-1 containing serum (1 × 10^6^ cells/12 mL), seeded in a petri dish (diameter = 10 cm), and incubated in a humidity atmosphere containing 5% CO_2_ for 3 days at 37°C. After confirming proliferation, the obtained AdSCs were passaged and used for the experiments. The identification of AdSCs obtained was performed out by flow cytometric analysis (Gallios, Beckman Coulter, Inc., Brea, CA, USA) using mesenchymal stem cell positive and negative antigen makers [45], and described in Supporting Information (Figure S1).

### 2.8. Viability of cells cultured in IP hydrogel

The murine fibroblast cell line L929 was obtained from the Health Science Research Resources Bank (HSRRB, Osaka, Japan). AdSCs were obtained as described above. After sterilization by filtering through a 0.45-μm filter, IP formulations were mixed with serum-free media (EMEM or ADSC-2) containing 2 × 10^4^ cells (L929 or AdSC). Aliquots (100 μL) were dispensed into sterilized Eppendorf tubes. After gelation by incubating for 30 minutes at 37°C, 200 μL of serum-free medium or medium containing FBS were gently placed onto the top of the IP hydrogel containing cells and incubated in a humidity atmosphere containing 5% CO_2_ at 37°C. After a predetermined period, the hydrogel was taken out, and 200 μL of the serum-free medium was further added. After breaking the hydrogel by repeated pipetting, a uniform suspension was obtained. The cells were precipitated by centrifuging and the supernatant was collected. This process was performed three times to wash the cells. The cells were stained by adding 100 μL of Live/Dead^TM^ reagent and left to stand for 30 minutes. Then, the cells were counted and cell viability was evaluated by observing cells under a microscope on a hemocytometer.

### 2.9. Observation of AdSC location in IP hydrogel

A vertical flat plate-type culture vessel (Figure S2) was prepared and sterilized by UV irradiation for 30 minutes. AdSCs were previously stained by Cell Tracker Green. The IP hydrogel was formed by placing the IP formulation containing AdSCs in the culture vessel and incubating at 37°C for 30 minutes. Serum-free ADSC-2 or ADSC-1 containing 5% FBS was then poured on the top of IP hydrogel and incubated in a humidity atmosphere containing 5% CO_2_ at 37°C. After culturing for a predetermined time, the medium was removed and observed on CLSM.

### 2.10. Measurement of mRNA expression in AdSC

An IP formulation (200 μL) containing 4 × 10^4^ AdSCs was added to an Eppendorf tube and incubated for 15 minutes at 37°C for gelation. ADSC-1 (400 μL) containing serum was placed on the hydrogel and incubated in a humidity atmosphere containing 5% CO_2_ at 37°C. When tissue culture polystyrene (TCPS) was used as a control, 4 × 10^4^ AdSCs in ADSC-1 (400 μL) were seeded in a 48-well dish (IWAKI, AGC Techno Glass Co., Ltd., Shizuoka, Japan) and incubated in a humidity atmosphere containing 5% CO_2_ at 37°C. Every 24 hours, 200 μL of the medium was removed and replaced with a fresh ADSC-1 medium (200 μL). After a predetermined time, cells were collected from the hydrogel as described above (section 2.8), and RNA extraction was performed using RNeasy Mini Kit. cDNA synthesis by reverse transcription was performed using PrimeScript^TM^ RT Reagent Kit. The amount of mRNA was estimated using real-time PCR (BIORAD CFX96 Deep well Real-Time System, Bio-Rad Laboratories Inc., Hercules, CA, USA) and SsoAdvanced Universal SYBR Green mix (Bio-Rad Laboratories Inc., Hercules, CA, USA). Relative RNA expression was determined using the formula: Rel ExP = 2^−ΔCt^, where ΔCt = Ct gene of interest – Ct GAPDH in experimental samples.

### 2.11. Determination of cytokine production of AdSC

In the experiment described in **2.10**, the supernatant medium of the cell culture was collected, transferred to an Eppendorf tube, and cryopreserved at −30°C. Before measurement, it was thawed and the vascular endothelial growth factor (VEGF) concentration was measured using an enzyme-linked immunosorbent assay (ELISA) kit for VEGF (Pepro Tech Inc., Cranbury, NJ, USA). Measurements were carried out by following the instructions of the reagent supplier.

### 2.12. Therapeutic effect on myocardial infarction model mice

The preparation of a myocardial infarction model and the evaluation of cardiac function were performed according to the previous report [36]. C57BL6/N mice (9 week-old, male) were anesthetized by intraperitoneal administration of 2,2,2-tribromoethanol (Avertin, Sigma–Aldrich Japan, Tokyo, Japan) at 400 mg/kg body weight. A myocardial infarction model was prepared by ligating the left anterior descending (LAD) branch of the coronary artery. Subsequently, dispersion of AdSCs in ADSC-1 (intact AdSC), an IP formulation (polymer concentration = 20 wt%) containing AdSCs (2 × 10^5^ cells in 20 μL), or IP formulation without cells was injected into the ischemic tissue. The mice were recovered in cages with free access to water and feed. In tracking the AdSCs three days after administration, AdSC collected from wild-type C57BL6/N was used by fluorescently labeling with DiI. For the experiments investigating cell persistence and cardiac function for 28 days post-administration, AdSC from *lacZ* transgenic mouse (B6.129S7-Gt(ROSA) 26Sor/J) (The Jackson Laboratories, Bar Harbor, ME, USA) was used. A cardiac ultrasonographic examination was performed to investigate the therapeutic effect of myocardial infarction. The left ventricular end-diastolic dimension (LVDd) and left ventricular end-systolic dimension were measured to determine Ejection Fraction (EF) (blood pumping volume per left ventricular volume), and left ventricular (LV) fractional shortening (FS) was calculated.

### 2.13. Histological analysis

The histological evaluation was performed as in the previous report [36]. Mice were euthanized 28 days after the induction of myocardial infarction. PBS and then 4% paraformaldehyde (PFA)/PBS was perfused from the right carotid artery and the heart was removed. The heart was immersed in 4% PFA/PBS for 6 hours and then in 20% sucrose/PBS overnight. It was embedded in an optimal cutting temperature (OCT) compound and frozen to prepare a 5-μm thick section approximately 5 mm the lower part of the ligation site. Fluorescent immune-staining was performed on each of the prepared sections. An antibody against β-galactosidase (β-gal) (rhodamine-labeled, MP Biomedicals, Santa Ana, CA) was used to detect the *LacZ* gene expressing exogenously infused AdSCs. FITC-labeled anti-isolectin-B4 (ILB4) (Vector Laboratories, Burlingame, CA USA) was used to detect the endothelial cells. Nuclei were counterstained with DAPI. The prepared sections were encapsulated with a water-soluble mounting medium and observed under a fluorescence microscope (BZ8000, Keyence, Osaka, Japan). The density of capillaries was estimated from the strongly magnified image (× 200). Masson’s trichrome staining was also performed on each prepared tissue section, and the ratios of the length and area of the fibrotic region (stained in blue) to the entire left ventricular tissue were determined using Image J software.

### 2.14. Statistical analysis

Data are expressed as means ± standard error of the mean (SEM) unless otherwise cited, and were analyzed with statistical analysis software for Microsoft Excel (Tokyo, Japan). Significant differences between mean values of two groups were evaluated using the Student’s *t*-test or the Mann–Whitney *U* test; those among multiple groups were tested via analysis of variance followed by post hoc testing with a Tukey procedure. Values of *p* < 0.05 were considered significant.

## 3. Results and Discussion

### 3.1. Preparation of the IP formulations

**Figure 3.**
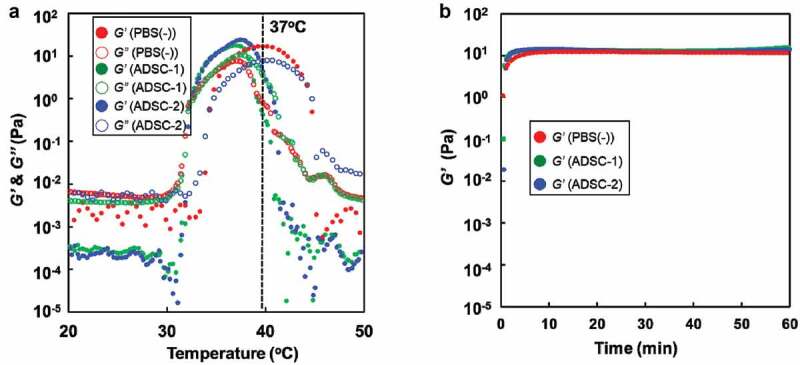
(a) Temperature-dependence of storage modulus (*G’*) (closed circle) and loss modulus (*G”*) (open circle) as a function of temperature for IP formulation (IP(P)) in PBS (-): red, ADSC-1 (medium containing 5% FBS): green, ADSC-2 (serum-free medium): blue. (b) Time course of storage (*G’*) modulus of IP formulation (IP(P)) after warming at 37°C in PBS (-) (red), ADSC-1 (green), ADSC-2 (blue). Polymer concentration = 15 wt%

Tri-PCGs (tri-PCG-1 and tri-PCG-2) and tri-PCG-Acryl were successfully synthesized as previously reported [19]. The characterizations of these polymers are summarized in Table 1. First, the viscoelastic properties of tri-PCG dissolved in PBS (-), ADSC-1 (medium containing 5% FBS), or ADSC-2 (serum-free medium) were investigated to estimate the influence of the solvents (polymer concentration = 15 wt%). The storage (G′) and loss moduli (G″) of the IP solutions were monitored on temperature increase (Figure 3(a)). Gel state and gelation temperature *T*_gel_ can be defined as the state where G′ > G″ and the temperature at which G′ take over G″, respectively. It was confirmed that these samples existed in a gel state at 37°C and showed *T*_gel_ < 37°C. The *T*_gel_ values are also shown in Table 2. The tendencies of the IP in the medium exhibiting narrower gel temperature ranges and storage modulus in the presence of FBS was slightly lower; however, these differences were not significant and all of the samples exhibited similar *T*_gel_ value. When this IP solution was kept at 37°C and measured, no significant difference in *G’* was observed (Figure 3(b)). These results reveal that ADSC-1 and ADSC-2 can be used as a solvent for IP formulation, and these samples can be regarded as almost identical on physical properties.

**Table 2.** Rheological properties of tri-PCG in various solvents

Solvent	*T*_gel_ (°C)	*G*′ at 37°C (Pa)	*G*′_max_ (Pa) and the temperature (°C)
PBS(-)	33.3	18.7	21.2 (38.3)
ADSC-1 (FBS(+))	32.3	17.4	17.4 (37.0)
ADSC-2 (FBS(-))	32.6	22.7	24.0 (37.5)

IP formulations containing tri-PCG/DPMP and tri-PCG-Acryl were prepared using the media for the respective experiments. The sample containing only tri-PCG, 16 wt% tri-PCG-Acryl, and 25 wt% tri-PCG-Acryl were defined as sample codes IP(P), IP(A16), and IP(A25), respectively, and their composition are summarized in Table 3. These IP solutions were confirmed to show irreversible sol-to-gel transition similar to that reported previously (Figure S3) [19].

**Table 3.** Sample codes of IP formulations and the contents

Code of IP formulation	tri-PCG-1 (%)	tri-PCG-Acryl (%)	DPMP (%)
IP(P)	100	-	-
IP(A16)	83.1	16.0	0.9
IP(A25)	73.9	24.6	1.4

### 3.2. Cell culture in IP hydrogel

**Figure 4.**
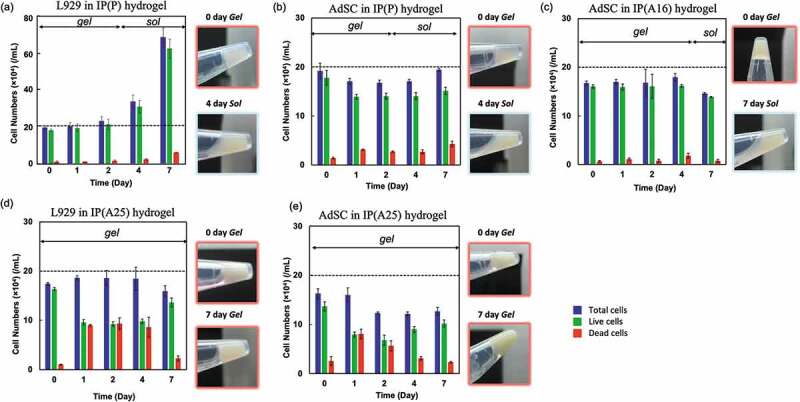
Proliferation of L929 cells or AdSCs cultured in IP hydrogels for 7 days at 37 °C. (a) L929 cells in IP(P) hydrogel, (b) AdSCs in IP(P) hydrogel, (c) AdSCs in IP(A16) hydrogel, (d) L929 cells in IP(A25) hydrogel, (e) AdSCs in IP(A25) hydrogel. Blue, green, and red bars represent total, live, and dead cell numbers, respectively. The dotted lines indicate the number of primary cells used in this experiment. The state of the IP formulation (gel or sol) during the culturing is indicated inside the graphs. The representative pictures for the cell-containing hydrogels in Eppendorf tubes are also presented. Cells (2 × 10^4^) were mixed with IP formulations at day 0 and incubated in IP hydrogels sunk in the serum-free medium (EMEM, ADSC-2). Polymer concentration = 15 wt%

The viabilities of L929 cells and AdSCs cultured in the IP(P) hydrogels for 7 days are shown in Figure 4(a and Figure 4b). The IP(P) hydrogel remained in a gel state for 3 days in the medium and entered sol state on day 4. When the cells (L929 cells and AdSCs) were collected immediately after gelation, it was confirmed that ≥ 90% of both cells were viable in the IP(P) hydrogel even after sol-to-gel transition. The results indicate that the sol-to-gel transition of the IP does not affect cell survival. Our previous study showed that the sol-to-gel transition of tri-PCG-based IP containing liposomes neither affected the stability of the entrapped liposomes nor internal content retention [22]. Although the IPs have surfactant-like amphiphilic structures and the hydrophobic interaction among the IP molecules occurred on the sol-to-gel transition, the transition of the IP system does not strongly perturb the lipid membrane, and therefore, the cells were retained in the hydrogel without being damaged. The number of viable L929 cells was almost constant for 2 days but increased after day 4 (Figure 4(a)) when the hydrogel transitioned to the sol state. Contrariwise, more than 70% of AdSCs were viable in IP(P) hydrogel for 7 days, but no significant increase in the cell numbers was observed (Figure 4(b)). The viability of AdSCs in IP(A16) hydrogel containing DPMP and 16 wt% tri-PCG-Acryl is shown in Figure 4(c). Similar results as in IP(P) were obtained. Figure 4(d and Figure 4e) show the results when using IP(A25) hydrogel for L929 cells and AdSCs, respectively. We observed that approximately 50% of the cells of both L929 cells and AdSCs died after a 1-day culturing. However, after 2-day incubation, percentages of live cells slightly increased, suggesting that cells had proliferated. We previously reported the cytotoxicity of the components [21]. The viability of L929 cells was decreased when incubated with tri-PCG-Acryl micelles or tri-PCG micelles containing DPMP at relatively high concentrations, but not with the mixture of them. Considering the results above and those of the previous study [21], the tri-PCG-Acryl acryloyl and DPMP thiol groups might exhibit some damage on L929 cells and AdSCs at relatively high concentrations. However, most acryloyl and thiol groups in this formulation reacted with each other within 24 h, and the reaction products are not harmful to the cells. The substantial concentration of these functional groups decreased below the toxic level. Besides, when culturing AdSCs in IP hydrogels using ADSC-1 containing serum, similar results to those of the serum-free medium were obtained (Figure S4).

**Figure 5.**
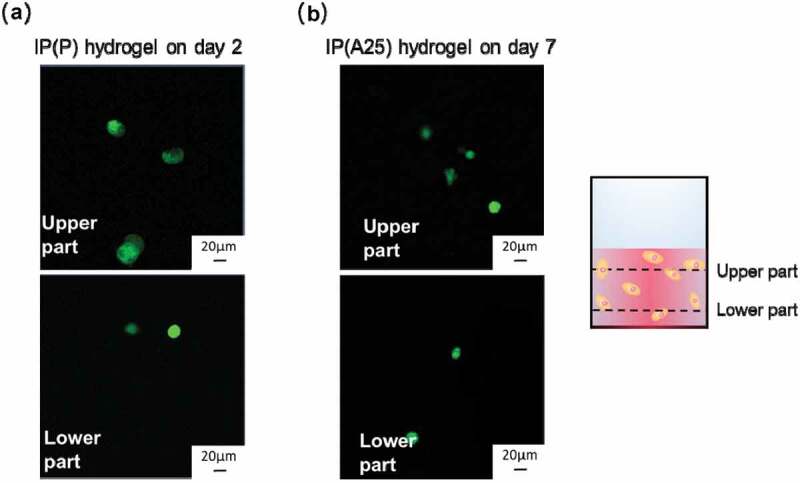
The location of AdSCs in (a) IP(P) and (b) IP(A25) hydrogels. Cells were previously stained with Cell-tracker Green. After incubation, the upper part and the lower part of IP hydrogels were observed with CLSM. Polymer concentration = 15 wt%

Next, to investigate the possible sedimentation of AdSCs cultured in the IP hydrogel, the location of the cells in a vertical flat plate-type culture vessel (Figure S2) was investigated. Cells in the IP(P) hydrogel were monitored on day 2, whereas those in the IP(A25) hydrogel were observed on day 7 after gelation and incubation, and these hydrogels maintained their gel states. The upper and lower parts in the vertical direction of the culture vessel were observed by CLSM, and the cell densities were compared (Figure 5). No change in cell density at both upper and lower parts was observed. We proposed that the cells cultured in the IP hydrogel maintained their locations in the hydrogel without sedimentation during the gel state.

From these results, it was confirmed that 1) AdSCs can be retained in the hydrogel while maintaining their positions, 2) approximately half of the AdSCs were alive, but the cell proliferation was suppressed 3) differences in proliferation behavior depending on the cell type occurred.　It is interesting that there was a difference in the cell growth rate in the IP hydrogel depending on the cell type, but the reason is not clear at this time. There was a report that cell cycles inside of soft hydrogel were different from those on normal cell culture [46]. Differences in cell proliferation rates and differentiation ability depending on the elastic modulus of gel-like materials surface were also reported [47,48]. It is well known that there is a relationship between cell proliferation and cell adhesion on the matrices. In soft hydrogels, the state of cell adhesion may differ depending on the cell type, which may lead to the difference in proliferation rate in the hydrogel depending on the cell type. Considering the chemical structure of the polymer that comprised the IP hydrogel used in this study, the environment surrounded by hydrophilic PEG chains in the hydrogel influenced the growth of AdSCs. It is also interesting that the AdSCs can be differentiated or can keep their pluripotency in the IP hydrogel. These issues will be reported in the subsequent reports.

### 3.3. Physiological functions of AdSCs cultured in IP hydrogel

**Figure 6.**
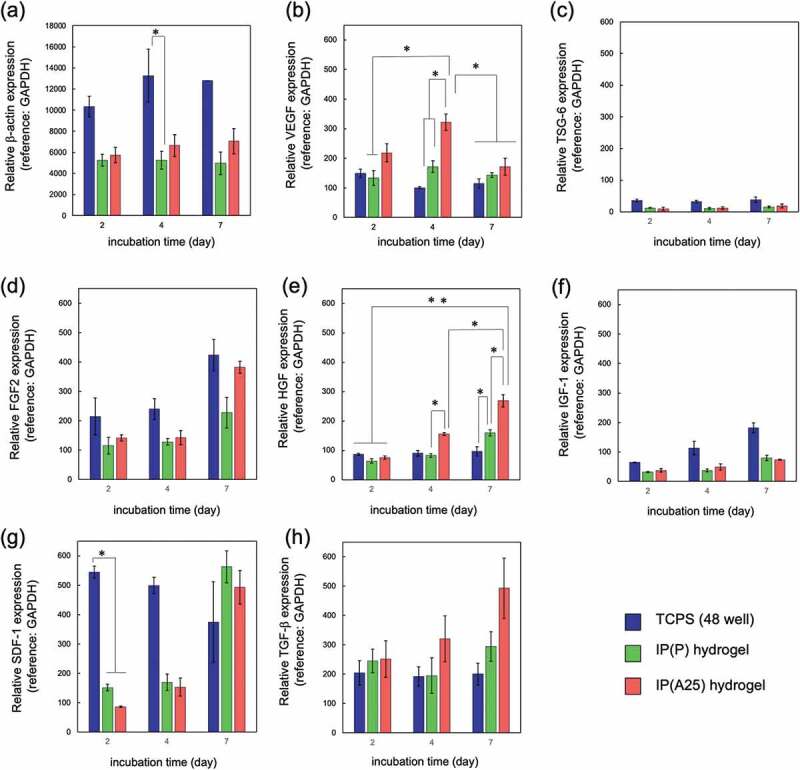
Relative mRNA expression of (a) β -actin, (b) VEGF, (c) TSG-6, (d) FGF2, (e) HGF, (f) IGF-1, (g) SDF-1, (h) TGF-β day 2, 4 and 7 after culturing on TCPS (blue) or in IP(P) hydrogel (green), or IP(A25) hydrogel (red) estimated by RT-PCR. The medium containing 5% FBS (ADSC-1) was used and replaced on day 2 and day 4. GAPDH was used as the reference gene. Polymer concentration = 15 wt%. The data represent the mean ± SD (n = 3). * *p* < 0.05, ** *p* < 0.01

To investigate the physiological function of AdSCs cultured in IP hydrogels (IP(P) and IP(A25)), mRNA expression for representative genes was investigated by reverse transcription real-time PCR on days 2, 4, and 7 after AdSC-containing IP hydrogels were prepared (Figure 6). The data obtained after culturing AdSCs in IP(P) and IP(A25) hydrogels were compared with those after culturing in a 48-well TCPS dish. When the expression level of β-actin was quantified using GAPDH as a reference gene, the expression levels of β-actin in IP(P) and IP(A25) hydrogels were decreased compared with those on TCPS (Figure 6(a)). Both GAPDH and β-actin are known as housekeeping genes; thus, either of them was fluctuating. Figure 6(b) shows the results for VEGF expression. The VEGF gene expression in IP(A25) was slightly higher than those in IP(P) and TCPS dish, and the difference reached a maximum on day 4. These results mean that the AdSCs cultured in IP(A25) maintained the production of VEGF, and the VEGF-producing ability was rather increased upon culturing in IP(A25) hydrogel. If the data for β-actin was used as a reference gene, the tendency would be the same or as significant as the results when GAPDH was used because the expression levels of β-actin in TCPS were higher than in IP hydrogels.

Other mRNA expressions were also investigated; the results are shown in Figure 6(c-Figure 6h). Among them, the expression levels of hepatocyte growth factor (HGF) (Figure 6(e)) and transforming growth factor (TGF-β) (Figure 6(h)) of IP hydrogel-cultured AdSCs were approximately equal to or higher than those in the TCPS dish. The results of IP(A25) hydrogel were higher than those of IP(P) hydrogel. Conversely, the expression levels of tumor necrosis-inducing gene 6 (TSG-6) (Figure 6(c)) and insulin-like growth factor (IGF-1) (Figure 6(f)) in both IP(P) and IP(A25) were lower than those in the TCPS dish. For fibroblast growth factor (FGF-2) (Figure 6(d)) and stromal cell-derived factor-1 (SDF-1) (chemokines, which are known to promote angiogenesis (Figure 6(d))), their expression levels in IP hydrogels were lower than in TCPS dish for the first 4 days but increased to levels similar to TCPS after 7 days. These differences in gene expression must be due to the elastic moduli-dependent differences in mechanical stimuli of the IP hydrogels and TCPS. Overall, we proposed that AdSCs cultured in IP(A25) hydrogels almost maintained their physiological properties and retain their ability to secrete major cytokines.

**Figure 7.**
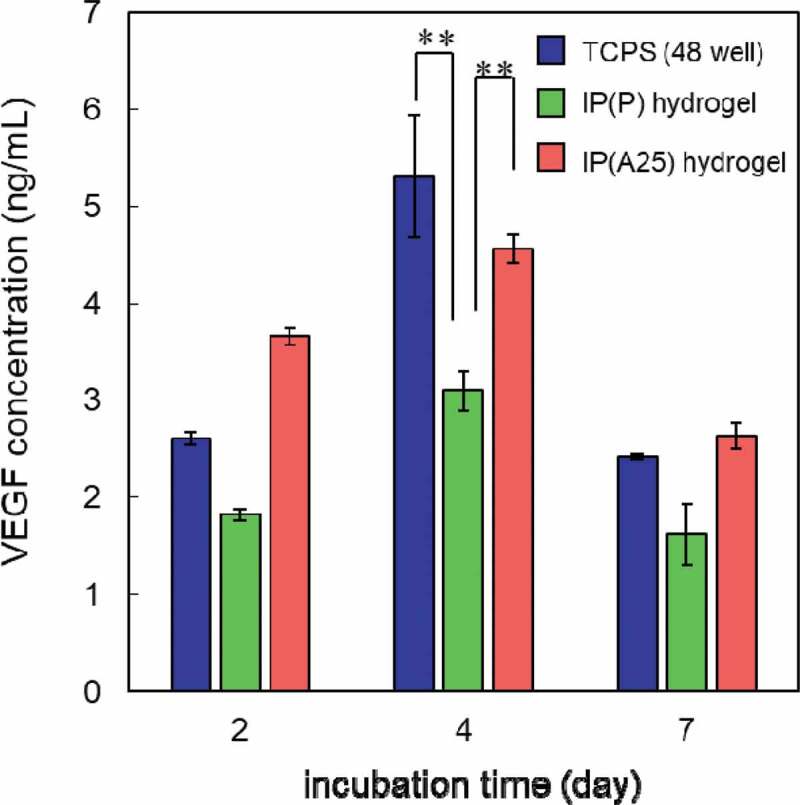
VEGF production from AdSCs cultured on TCPS (blue) or in IP(P) (green) and IP(A25) (red) hydrogels for 2, 4, and 7 days estimated by ELISA. Polymer concentration = 15 wt%. The data represent the mean ± SD (n = 3). **P < 0.01

Moreover, to confirm actual cytokine secretion from AdSCs cultured in IP hydrogels, the amount of VEGF in the medium was estimated by ELISA (Figure 7). AdSCs cultured in IP(A25) hydrogel showed higher VEGF production than those cultured on TCPS on day 2 and showed similar levels on days 4 and 7. VEGF production in IP(P) hydrogel was lower than IP(A25) and TCPS throughout the observation. These results seem to be somewhat inconsistent with the results of mRNA expression shown in Figure 6(b). However, the mRNA expression is represented as data per cell, while the cytokine (VEGF) production is data per well, which reflects the total cell numbers in the well. A significant increase in numbers of AdSCs cultured on TCPS was observed (Figure S5) compared with those in IP hydrogels (Figure S4). Considering the cell numbers in each well, the data shown in Figure 7 were consistent with the quantitative result of mRNA expression level. An amount of VEGF was detected in the supernatant medium on IP hydrogels containing AdSCs. Thus, these results mean that the AdSCs in IP(A25) hydrogel maintained VEGF production ability and VEGF could be released from the IP(A25) hydrogel. We suggested that a cytokine-releasing hydrogel system can be provided by injecting an IP formulation that entraps cytokine-producing AdSCs *in situ*.

It is interesting that enhanced production of VEGF and some other cytokines were observed in AdSC cultured in IP(A25) compared with IP(P). However, the mechanism for this enhancement is unclear at present. IP(A25) hydrogel has a higher storage modulus due to the formation of chemical crosslinks than IP(P). One of the possibility is that AdSCs detected the difference in elastic modulus and accelerate the cytokine production. However, further experiments are needed to elucidate the detailed mechanism. We are currently conducting an experiment to investigate changes in the mRNA expression level of cells encapsulated in hydrogel by continuously changing the elastic modulus of IP. We are planning to publish this matter in the next paper.

### 3.4. Retention of AdSCs injected at ischemic heart

**Figure 8.**
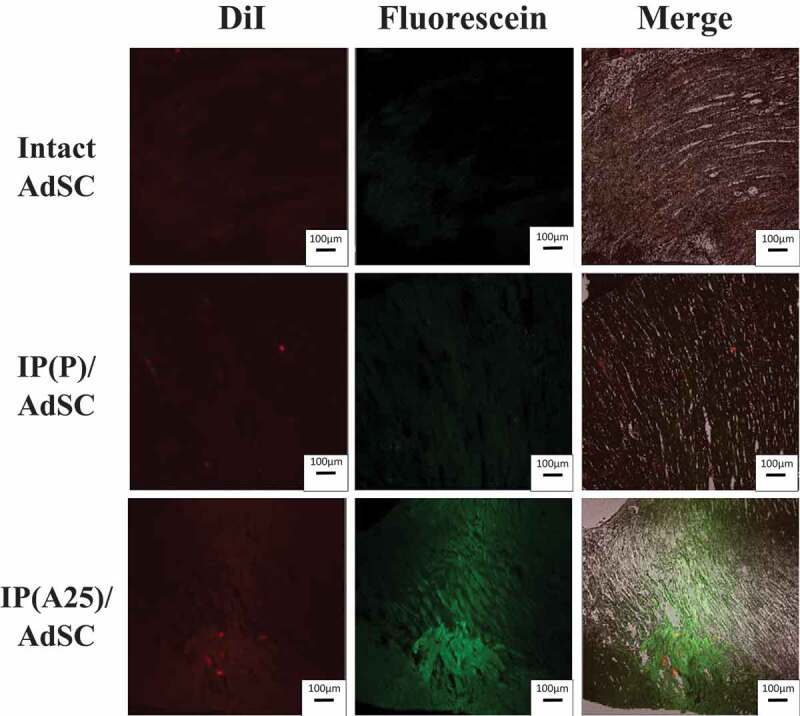
Fluorescence images of the cryosection of heart extracted from the mouse injected with intact AdSC, AdSC-containing IP(P) (IP(P)/AdSC), or AdSC- containing IP(A25) (IP(A25)/AdSC). The AdSCs were stained with DiI. IP hydrogel was labeled by adding a small amount of fluorescein-labeled tri-PCG. AdSCs (2 × 10^5^) were dispersed in 20-µL medium (ADSC-1) or mixed in IP formulations, and then injected into the cardiac infarction site. After 3 days, the heart was extracted and fixed with formalin

The survival of AdSCs and the production of VEGF in the IP hydrogel led us to investigate the potential use of AdSC delivery via IP to the myocardial infarction site. Before evaluating the therapeutic potential for ischemic heart, we investigated retention of the IP hydrogel and AdSCs at the injected site near myocardial infarction (Figure 8). The AdSC was stained by DiI and the IP(A25) hydrogel was labeled by mixing with a small amount of fluorescein-labeled tri-PCG. Intact AdSCs (dispersion in ADSC-1), IP(A25) formulation containing AdSCs (IP(A)/AdSC and IP(A25)/AdSC) was injected into an artificially produced myocardial infarction site in mice. The retention of AdSCs and IP hydrogel at the injection site was investigated for 3 days after the injection. When intact AdSCs were administered, red fluorescence of DiI from AdSCs was not observed in the heart. However, in the groups administered with IP(P)/AdSC or IP(A25)/AdSC, bright spots of red fluorescence were observed. The IP(A25)/AdSC showed more red spots and stronger fluorescence intensity than that of IP(P)/AdSC. Moreover, regarding IP(A25)/AdSC, stronger green fluorescence from fluorescein-labeled tri-PCG was observed than in IP(P)/AdSC. These results suggested that IP(A25) hydrogel demonstrates a longer retention time *in vivo* than IP(P). Using IP(A25) formulation, the possibility of the persistence of AdSCs at the injected site was increased compared with using intact AdSCs or IP(P) formulation. Therefore, we reason that the AdSCs administered with IP(A25) remain at the injected site, and release cytokines there for a definite period. Therefore, in the following *in vivo* experiments, we used IP(A25), which forms chemically cross-linked hydrogel on sol-to-gel transition, shows a longer gel state duration, and is expected to retain active AdSCs for a long period at the site of myocardial infarction.

### 3.5. Therapeutic effects of IP hydrogel-delivered AdSCs

**Figure 9.**
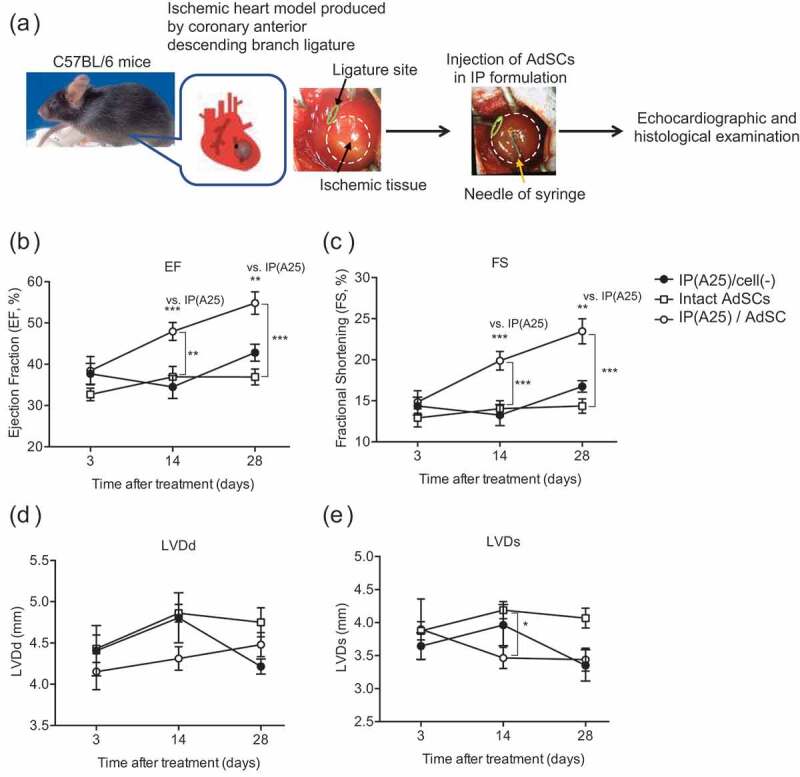
(a) Schematic image of treating ischemic heart model. (b-e) Cardiac functions were evaluated by echocardiography on days 3, 14 and 28; (b) Ejection fraction (EF), (c) Fractional shortening (FS), (d) Left ventricular end-diastolic dimension (LVDd), and (e) Left ventricular end-systolic dimension (LVDs). Closed circle: IP(A25)/cwll(-), open square: intact AdSC, open circle: IP(A25)/AdSC. * *p* < 0.05, ** *p* < 0.01, *** *p* < 0.001

We investigated the therapeutic effects of AdSCs with IP(A25) formulation for myocardial infarction model mice. AdSCs collected from *lacZ* transgenic mice were mixed with IP(A25) formulation and injected into the myocardial infarction site in mice (Figure 9(a)). The injected hydrogel did not leak, and the color of the myocardial infarction site changed slightly. It was considered that almost all AdSCs are present at the administration site together with the hydrogel after administration. Echocardiographic examination for mice cardiac function was performed 3, 14, and 28 days after inducing myocardial infarction (Figure 9(b-Figure 9e)). Although the ejection fraction (EF) (indicating cardiac output) and the left ventricular fractional shortening (FS) (indicating myocardial contractility) did not increase in the group receiving IP(A25) without cells (IP(A25)/cell(-)) or intact AdSC, these values significantly recovered over time in the group receiving IP(A25) formulation containing AdSC (IP(A25)/AdSC). In the IP(A25)/AdSC-administered, the left ventricular end-diastolic diameter (LVDd) increased while the left ventricular end-systolic diameter LVDs decreased. The left ventricular fractional shortening (FS) (%) of normal mice were reported to be approximately 41–52% [49], and no recovery in PBS-administrated mice of the same model mice were observed as shown in the reference [36]. As shown in Figure 9(c), the FS value after 28 days for IP(A25)/AdSC-administration group was about 23%. Therefore, cardiac function was not completely recovered, but some recovery was surely observed. These results suggest that administration of AdSCs with IP(A25) hydrogel to the site of myocardial infarction caused regeneration of myocardial contractile force and promoted cardiac function recovery.

**Figure 10.**
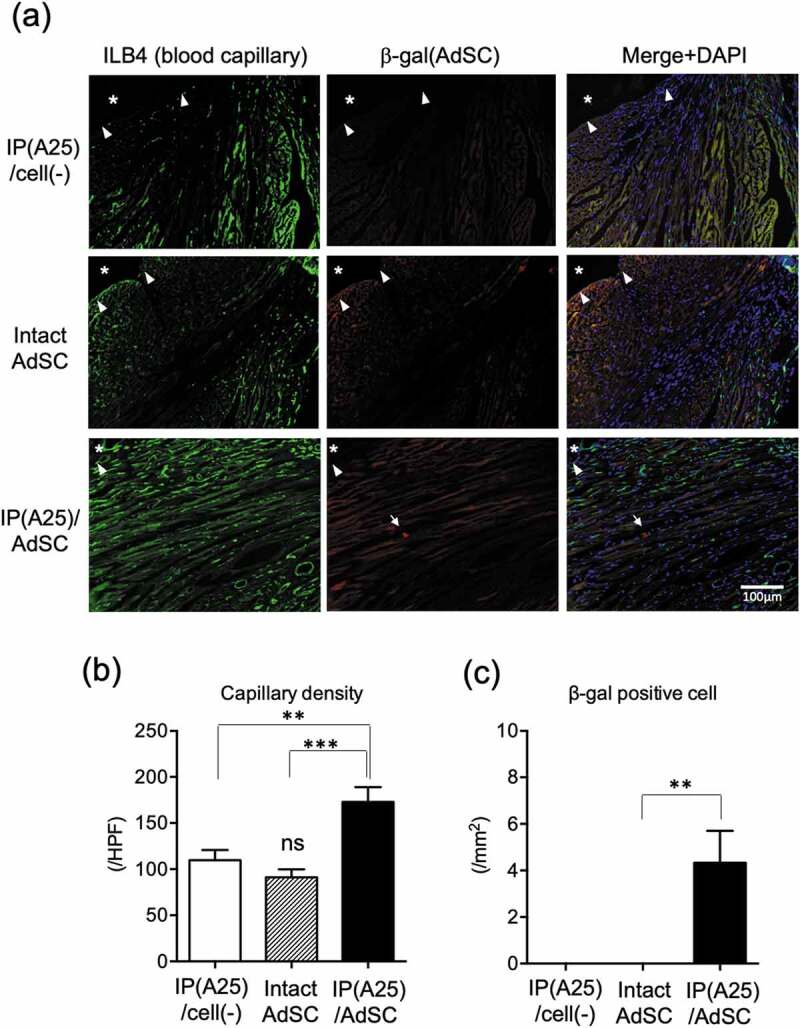
(a) Immunofluorescence staining of mice heart section after 28 days. The asterisk represents the left ventricular cavity, the triangle represents the endocardium, and the arrow represents the β-galactosidase-positive cell (= AdSC). Quantitative data for (b) capillary density and (c) the number of β-galactosidase-positive cells (=AdSCs) detected around the ischemic part. ** *p* < 0.01, *** *p* < 0.001

To investigate the mechanism of AdSC cardiac function recovery with IP(A25), the hearts of treated mice were removed 28 days after the onset of myocardial infarction, and histological evaluations were performed. Figure 10(a) shows the images of the capillary endothelium (ILB4) and the injected AdSC (β-gal) stained by immunofluorescence staining in green and red, respectively. Quantitative data for capillary density and the number of β-gal-positive cells/area are shown in Figure 10(b and Figure 10c), respectively. The highest green fluorescence of vascular endothelium was observed in the IP(A25)/AdSC-administered group, indicating the existence of several blood capillaries. Furthermore, bright red spots indicating β-gal positive cells were observed in the IP(A25)/AdSC-administered group (about 4 spots/1 mm^2^) only. These results suggested that IP(A25) formulation improved the number of remaining AdSCs at the injection site, and the AdSCs produced cytokines such as VEGF, which is essential for inducing capillaries. β-Gal-positive cells mean AdSCs remained at the injected site, but few cells were. Thus, we reason that most of the AdSCs died within 28 days, and the recovery of cardiac function is not due to transplanted AdSCs differentiating into cardiomyocytes, but more likely because of cytokines secreted by the AdSCs as paracrine effects.

**Figure 11.**
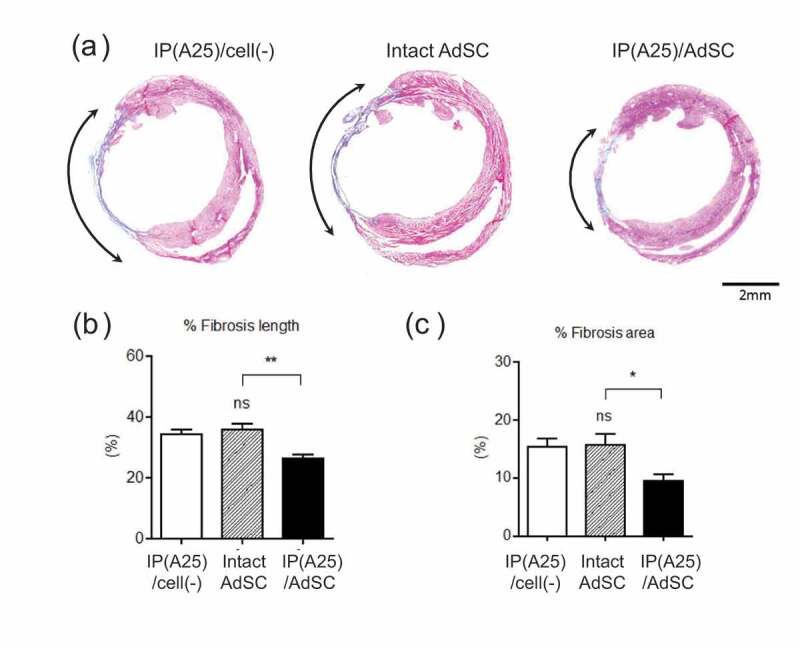
(a) Section of mice hearts stained by Masson’s trichrome staining after 28 days. Fibrotic regions are indicated with arrows. Quantitative data for the ratio of the length (b) and area (c) of the fibrotic region to the entire left ventricular tissue

It is known that when myocardial infarction occurs, cell shedding and fibrosis follow due to myocardial necrosis at the ischemic site. To evaluate the fibrotic state of the myocardial tissue, the length and area of the fibrotic site are estimated from the images of Masson’s trichrome-stained tissue sections 28 days after inducing myocardial infarction (Figure 11). In Figure 11(a), the blue-stained section indicates collagen (shown by curved arrows), that is, fibrotic tissue, and the red portion indicates myocardium. The fibrotic part was smaller in the IP(A25)/AdSC administration group than in the intact AdSC-administered and IP(A25)/cell(-)-administered groups. These results suggest that in the IP(A25)/AdSC-administered group, the AdSCs secreted angiogenesis-promoting factors such as VEGF *in situ* and promoted ischemic condition recovery. Moreover, no undesirable inflammatory phenomena were observed. We reason that the improvement of both AdSC persistence by IP hydrogel and ischemic state by AdSC-secreted cytokines contributed to cardiac function recovery.

In previous studies, besides direct injection of AdSCs [36–40], transplanting of cell sheets [50] and transplanting cardiomyocytes together with a conductive polymer scaffold [51] have been reported. Compared to the direct injection of AdSC, our method is characterized by the combined use of AdSCs and biodegradable IP hydrogel to prolong the retention time of the AdSCs at the injected site. On the other hand, it has been reported by another group that injecting a biodegradable cell-free hydrogel into the myocardial infarction site reduced fibrosis and cardiomyocyte hypertrophy and promotes myocardial tissue regeneration [52]. This is because the biodegradable hydrogel injected into the myocardial infarction site maintained the thickness of the ventricular wall after the induction of myocardial infarction for a certain period to prevent fibrosis and cardiomyocyte hypertrophy. On the contrary, it has been reported that when non-degradable PEG hydrogel was injected into the myocardial infarction site, fibrosis and hypertrophy of myocardial tissue occur to the same extent as in the PBS-administered group [53]. This can be interpreted that non-degradable hydrogel remained for a long time and induces fibrosis and cardiomyocyte hypertrophy by causing chronic inflammation. Therefore, more efficient treatment of myocardial infarction can be realized by using biodegradable IP hydrogel and AdSC together. In Figures 9–11, the combined use of IP hydrogel and AdSC improved cardiac function and suppressed fibrosis. This method can be performed by a very simple method of mixing cells with IP formulation. In addition since the IP formulation containing cells can be easily injected, it can be used in minimally invasive surgery using a thoracoscope. Furthermore, since it is possible to mix not only cells but also drugs, this method has an advantage on possibility to improve the therapeutic effect of myocardial infarction by sustained release of the drug from the IP hydrogel.

## 4. Conclusion

This study demonstrated an AdSC delivery using a temperature-responsive biodegradable IP system and the potential application for treating ischemic heart disease. AdSCs could be cultured in the IP hydrogel *in vitro*. The effective gene expression and secretion of VEGF from entrapped AdSCs in the IP hydrogel were confirmed especially when an IP(A25) forming chemical cross-linking hydrogel was used. When the IP(A25) formulation containing AdSCs was injected into the heart of myocardial infarction model mice, improvement in AdSC persistence at the injection site, blood capillary density increase at the ischemic tissue, and cardiac function recovery were observed. This suggested that the recovery of cardiac function is due to the longer retention of AdSCs in ischemic tissue via IP(A25) hydrogel support and angiogenesis-inducing cytokine (VEGF) release. This study results suggest that the AdSC-associated chemical cross-linkage-forming IP hydrogel system that exhibits longer retention time *in vivo* is a promising therapeutic method for ischemic heart disease, and the IP system can also be applied in cellular therapy for other cell and disease combinations.

## Supplementary Material

Supplemental Material

Supplemental Material
