# Supplementary material for: Cellular therapy for myocardial ischemia using a temperature-responsive biodegradable injectable polymer system with adipose-derived stem cells
Source: Sci Technol Adv Mater. 2021 Aug 6;22(1):627–42. doi: 10.1080/14686996.2021.1938212 (PMC8354160; doi:10.1080/14686996.2021.1938212)
Supplement: Supplemental Material [file TSTA_A_1938212_SM1517.pdf]

Biomaterials Science

PAPER

Cite this: *Biomater. Sci.*, 2017, **5**, 1304

Biodegradable injectable polymer systems exhibiting a longer and controllable duration time of the gel state†

Yasuyuki Yoshida,‡[a] Hiroki Takai,[a] Keisuke Kawahara,[a] Shintaro Mitsumune,[a] Kazuyuki Takata,[a] Akinori Kuzuya[a,b,c] and Yuichi Ohya *[a,b]

Received 19th April 2017, Accepted 22nd May 2017

DOI: 10.1039/c7bm00357a

rsc.li/biomaterials-science

Here, we report biodegradable temperature-triggered covalent gelation systems exhibiting a longer and controllable duration time of the gel state by a "mixing strategy" utilizing a thiol–ene reaction. We synthesized a tri-block copolymer of poly(caprolactone-*co*-glycolic acid) and PEG (tri-PCG) as a temperature-responsive injectable polymer (IP) and attached acryloyl groups on both termini (tri-PCG-Acryl). A tri-PCG micelle solution containing hydrophobic hexa-functional polythiol (Solution-A) and a tri-PCG-Acryl micelle solution (Solution-B) were mixed together. After mixing, the solution was still in the sol state at r.t., but exhibited an irreversible sol-to-gel transition in response to temperature. The duration time of the gel state while soaking in PBS could be altered from 1 day to 93 days by changing the mixing ratio of Solution-A/B. The physical strengths of the hydrogels were also controllable by changing the mixing ratio. The IP system showed good biocompatibility and a long duration time of the gel state after subcutaneous implantation.

Introduction

Some types of polymer aqueous solutions exhibit sol-to-gel transitions in response to a temperature increase. Such thermo-gelling polymer systems have attracted great attention in the past two decades as implantable biomedical materials. In particular, a biodegradable thermo-gelling polymer aqueous solution with a sol-to-gel transition point (T_{gel}) between room temperature (r.t.) and body temperature is expected to be useful as an injectable polymer (IP) system.[1–5] A sol state IP solution at r.t. can easily entrap living cells or drugs, and the solution can be easily injected into the body with a syringe to become a hydrogel at the injected site. Based on their favourable properties, such as biodegradability, biocompatibility, physical softness, high swelling with water, *etc.*, biodegradable IP systems are expected to be applied as scaffolds for tissue engineering,[6–8] drug delivery systems (DDSs),[9–11] adhesion prevention materials,[12–14] embolization materials[15–17] and endoscopic submucosal dissection (ESD).[18] Several kinds of amphiphilic block copolymers consisting of hydrophobic biodegradable aliphatic polyesters and hydrophilic poly(ethylene glycol) (PEG) of a relatively small molecular weight (MW), which can be excreted from the kidneys, have been reported as typical examples of biodegradable IPs for biomedical applications.[3–5,19–25]

Such temperature-responsive IP systems exhibit rapid sol-to-gel transitions to form hydrogels by the physical (non-covalent) cross-linking (aggregation) of polymers. However, one of the practical problems for these temperature-responsive gelation systems is the short duration time of the gel state in the body. The formed hydrogel tends to revert to the sol state within a short period (typically less than 24 h) where a large amount of water or body fluid exists, such as in the intraperitoneal space and interior of blood vessels, because the main driving force of gelation in these systems is a non-covalent hydrophobic interaction and gel formation is an equilibrium process. This property might be a disadvantage for the application of biodegradable IP systems as implant biomedical materials.

Alternatively, *in situ* gelation systems by chemical (covalent) cross-linking such as polymerization systems and coupling reaction systems have been reported.[26–30] The covalent gelation systems have advantages of a longer duration time of the gel state and a relatively high mechanical strength of the obtained

[a]Department of Chemistry and Materials Engineering, Faculty of Chemistry, Materials and Bioengineering, 3-3-35 Yamate, Suita, Osaka 564-8680, Japan. E-mail: yohya@kansai-u.ac.jp
[b]Organization for Research and Development of Innovative Science and Technology (ORDIST), Kansai University, Suita, Osaka 564-8680, Japan
[c]PREST, Japan Science and Technology Agency, 4-1-8 Honmachi, Kawaguchi, Saitama 332-0012, Japan

†Electronic supplementary information (ESI) available. See DOI: 10.1039/c7bm00357a

‡Research Fellow of the Japan Society for the Promotion of Science.

 This journal is © The Royal Society of Chemistry 2017

hydrogels, but also have several problems. In polymerization systems, how and when the polymerization starts is the issue. UV irradiation and photoinitiators are potentially toxic, and polymer chains produced by polymerization are not biodegradable. In coupling reaction systems, it is generally difficult to control the gelation time, and a special syringe for mixing of two solutions is needed. Therefore, biodegradable IP systems having the advantages of both the temperature-responsive physical gelation system and the covalent bond formation system are desired to achieve favourable gelation times, longer duration times of the gel state and biodegradability. Block copolymers of PEG and polycaprolactone (PCL)[20–22,31] or stereocomplex (SC) formation of polylactide (PLA) blocks[32–34] were reported to show irreversible gelation and provided some solutions to these issues by crystallization of the PCL block and SC formation of PLLA and PDLA. However, the duration times of the gel state were still not very long.

A biodegradable IP system with a longer and controllable duration time of the gel state should have a great advantage as implantable biomedical materials. For example, the sustained release of drugs from the hydrogel during the desired period may be achieved if the drug release occurred in a degradation-dependent manner. An ideal drug releasing period depends on the kind of drug. The IP hydrogel with a controllable degradation period must be useful for application as a drug releasing depot. Moreover, as a scaffold for regenerative medicine, the ideal degradation periods and moduli were different for the kind of tissue to be regenerated and the cell growth rate, such as bone, cartilage or soft tissues. The IP hydrogel systems with controllable degradation periods and moduli can provide wide varieties of scaffolds for the regeneration of different tissues.

Recently, we reported a temperature-triggered covalent gelation system by a "mixing strategy" using an IP having reactive succinimide ester (OSu) groups at the termini and water-soluble polyamines.[35] We synthesized a linear triblock copolymer consisting of PEG and a copolymer of ε-caprolactone (CL) and glycolic acid (GA) (PCGA), PCGA-*b*-PEG-*b*-PCGA (tri-PCG), as a temperature-responsive IP, and attached OSu groups at both termini. The obtained tri-PCG/tri-PCG-OSu mixture micelle solution was further mixed with a solution of a water-soluble polyamine, such as poly-L-lysine (PLys). The solution remained in the sol state after mixing at r.t., but underwent gelation in response to a temperature increase. Once formed, the hydrogel stayed in the gel state even after cooling, *i.e.*, the sol-to-gel transition was irreversible. In this system, OSu groups at the copolymer termini and primary amino groups of the polyamines existed separately in the micelle core and aqueous phase at r.t., respectively, and covalent bond formation between them occurred only upon the sol-to-gel transition induced by the temperature increase, where the micelle core was exposed to the aqueous phase and OSu could attack the amino groups of the polyamine. The formed hydrogel exhibited a longer duration time of the gel state under soaking in phosphate buffered saline (PBS) and the period was controllable from 1 day to 12 days.[35] However, this system uses an amide bond formation reaction between OSu groups and amino groups. Amino groups exist in many biomolecules, and OSu groups can react with these biomolecules located in blood, body fluid and cell surfaces. This may be an advantage in some cases, but may also have unexpected adverse effects in other cases.

In this study, we focused on a Michael-addition type (non-radical) thiol–ene reaction as a bio-orthogonal reaction,[36–39] *i.e.*, the chemical reaction can occur inside living systems without interfering with native biochemical processes. In addition, thiol–ene reactions produce no by-product, whereas the amide coupling reaction of OSu and NH_2 produces *N*-hydroxysuccinimide (NHS) as a by-product. Here, we designed temperature-triggered covalent gelation systems exhibiting longer and controllable duration times of the gel state by a "mixing strategy" utilizing the thiol–ene reaction. Acryloyl groups were introduced on both the termini of tri-PCG to give tri-PCG-Acryl. We chose dipentaerythritolhexakis(3-mercaptopropionate) (DPMP) as a commercially available hexa-functional polythiol to form chemical cross-links with acryloyl groups by the thiol–ene reaction. This molecule has low solubility in aqueous solution due to its hydrophobicity and should be incorporated into the core of the tri-PCG micelle. The obtained solution of DPMP-loaded tri-PCG micelles was mixed with a tri-PCG-Acryl micelle solution. We found that the mixed solution exhibited a temperature-responsive irreversible sol-to-gel transition. In this paper, we report on the temperature-responsive sol-to-gel transition behaviour and the irreversibility of the mixed system of DPMP-loaded tri-PCG micelles + tri-PCG-Acryl micelles. The differences compared to the previous OSu/amine system where the polyamine existed in the aqueous phase[35] are discussed. Moreover, the physical properties of the hydrogel, the duration time of the gel state under physiological conditions, and the biocompatibility of the hydrogels are investigated.

Experimental

Materials

PEG (MW = 1540 Da) (PEG_{1500}), CL, tin 2-ethylhexanoate [$Sn(Oct)_2$], *N*,*N*′-dicyclohexylcarbodiimide (DCC), and 4-dimethylaminopyridine (DMAP) were purchased from Wako Pure Chemical Industries Ltd (Osaka, Japan). Glycolide (GL) was obtained from Musashino Chemical Laboratory, Ltd (Tokyo, Japan). Acrylic acid was purchased from Sigma–Aldrich (St. Louis, USA). DPMP was a gift from SC Organic Chemical Co., Ltd (Osaka, Japan). Sprague–Dawley (SD) rats (7 weeks old, average body weights 150 g) were purchased from Japan SLC, Inc. (Hamamatsu, Japan). Water was purified by using a Milli-Q (Millipore) system. The other reagents were commercial grade and used without further purification.

Measurements

^1H nuclear magnetic resonance (^1H-NMR) spectra were recorded on a nuclear magnetic resonance spectrometer

(400 MHz, JNM-GSX-400, JEOL) using a deuterated solvent ($CDCl_3$). The chemical shifts were calibrated against tetramethylsilane (TMS) and/or solvent signal. The number-average molecular weights (M_n) were calculated from ^{1}H-NMR spectra. The weight-average molecular weights (M_w) and polydispersity indexes (M_w/M_n) of the polymers were determined by size exclusion chromatography (SEC) (column: TSKgel Multipore H_{XL}-M × 2, detector: RI). The measurements were performed using dimethylformamide (DMF) as an eluent at a flow rate of 1.0 mL min^{-1} at 40 °C using a series of PEGs as standards.

Synthesis of tri-PCG and tri-PCG-Acryl

The PCGA-*b*-PEG-*b*-PCGA triblock copolymer (tri-PCG) was synthesized by ring-opening copolymerization of CL and GL in the presence of PEG_{1500} as a macroinitiator and $Sn(Oct)_2$ as a catalyst at 160 °C for 12 h according to a method reported previously.[25] The syntheses were carried out by changing monomer feeds to give tri-PCG-1 and tri-PCG-2 having different PCGA lengths and CL/GA ratios. Tri-PCG-1 was used for the preparation of the IP formulations, and tri-PCG-2 was used to synthesize tri-PCG-Acryl.

Tri-PCG-Acryl was synthesized according to the method described in Scheme 1. DCC (6.24 g, 30.2 mmol) dissolved in dichloromethane (25 mL) was added dropwise into acrylic acid (2.17 g, 30.1 mmol) in dichloromethane (3 mL), and stirred at 0 °C for 2 h. Tri-PCG-2 (20.3 g, 5.08 mmol) and DMAP (616 mg, 5.04 mmol) dissolved in dichloromethane (40 mL) were added to the reaction mixture, and further stirred at r.t. for 24 h. The reaction mixture was filtered by suction filtration to remove dicyclohexylurea as a by-product. The filtrate was evaporated, and the obtained solid was reprecipitated using chloroform (40 mL) as a good solvent and a mixture of *n*-hexane and ethanol (8/2, v/v) (1000 mL) as a poor solvent. The obtained white solid was dried under vacuum for 72 h to give tri-PCG-Acryl.

Preparation of IP formulations

The IP formulations were prepared by the following method (Scheme 2). Aqueous solutions of DPMP-loaded tri-PCG-1 micelles were prepared by a solvent evaporation method as follows. Given amounts of tri-PCG-1 and DPMP were dissolved in acetone (copolymer concentration: 100 mg mL^{-1}) in a glass vial at r.t. The mixture solution was added dropwise into pure water (final copolymer concentration: 10 mg mL^{-1}). The mixture solution was stirred at r.t. for 5 min, and then sonicated in a bath-type sonicator at 0 °C for 30 min to give a clear solution. After removal of acetone by evaporation, the clear micelle solution was lyophilized to give powdery DPMP-

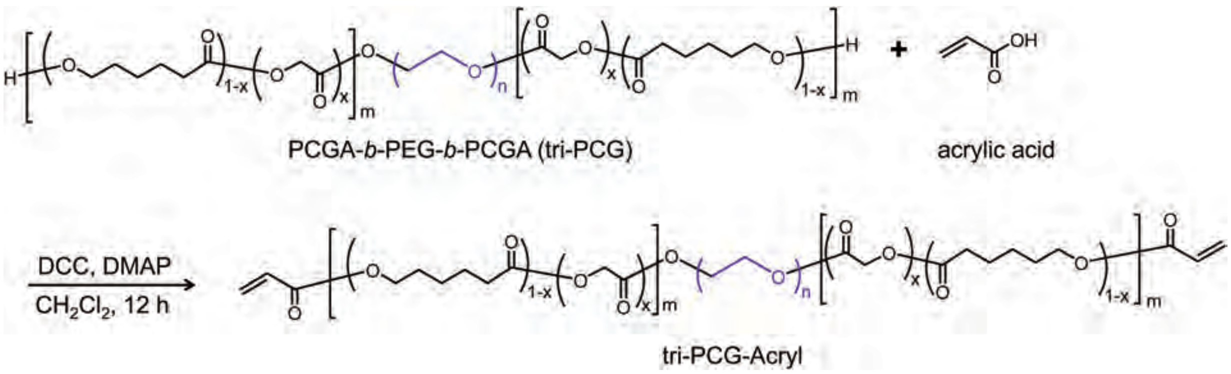

Scheme 1 Synthesis of tri-PCG-Acryl.

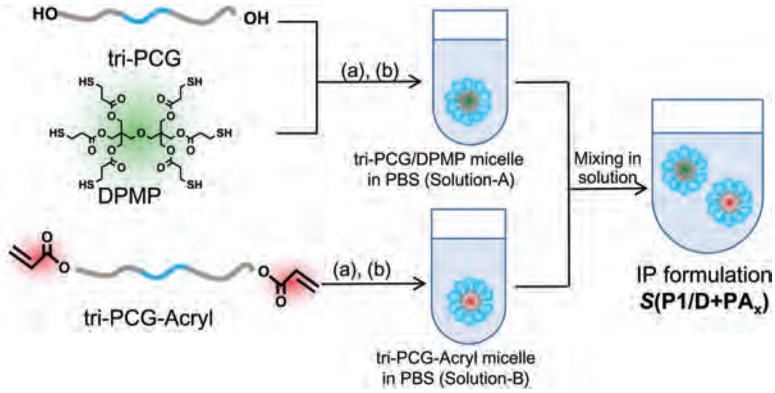

Scheme 2 Preparation of IP formulations. (a) Solvent evaporation: (i) dissolved in acetone, (ii) poured into pure water and evaporation to remove acetone. (b) (i) Freeze-dried, (ii) dissolved in PBS, repeating heat at 60 °C and cool at 0 °C.

This journal is © The Royal Society of Chemistry 2017

loaded micelles. A given amount of powdery DPMP-loaded micelles was placed in a vial, and PBS (10 mM, pH = 7.4) was added to the sample tube. After shaking with a vortex mixer for 1 min at r.t., the obtained dispersion was heated at 60 °C, sufficiently above the melting temperature (T_m) of the copolymers, for 5 s, and further stirred with a vortex mixer for 1 min at r.t. The tube was immersed in ice-cold water for 10 min. These procedures were repeated until no insoluble particles were observed. The obtained solution was further sonicated in a bath-type sonicator for a few minutes to give a clear solution. The pH of the solution was adjusted to 7.4 by adding a small amount of 1 M NaOH aq. or HCl aq. to give DPMP-loaded tri-PCG-1 micelles in PBS (Solution-A, 25 wt%). Confirmation of incorporation of DPMP into tri-PCG micelles was carried out by ^{1}H-NMR spectroscopy (see the ESI†). The tri-PCG-Acryl micelles in PBS were also prepared by the same method described above (Solution-B, 25 wt%). Solution-A (25 wt%) and Solution-B (25 wt%) were then mixed together at various ratios at r.t. to give an IP formulation (total polymer concentration = 25 wt%). Mixing ratios of Solution-A/Solution-B were 1/1, 1.5/1, 1.8/1, 2/1 and 5/1, whose weight contents of tri-PCG-Acryl in the total polymer were 50, 40, 36, 33 and 17 wt%, respectively. The amount of DPMP in each sample was adjusted in advance to achieve a ratio of SH/acryl = 1/1. The obtained IP formulations are expressed as ***S*(P1/D + PA*x*)**, where **P1**, **D** and **PA** mean tri-PCG-1 (**P1**) containing DPMP (**D**) and tri-PCG-Acryl (**PA**), and ***x*** refers to wt% of tri-PCG-Acryl in the total polymer. Thus, the formulation containing DPMP with 50, 40, 36, 33 and 17 wt% of tri-PCG-Acryl in the total polymer are expressed as ***S*(P1/D + PA50)**, ***S*(P1/D + PA40)**, ***S*(P1/D + PA36)**, ***S*(P1/D + PA33)**, and ***S*(P1/D + PA17)**, respectively. The control samples, tri-PCG-1 alone, tri-PCG-Acryl alone, a mixture of tri-PCG-1 and tri-PCG-Acryl without DPMP, and tri-PCG-1 containing DPMP, are expressed as ***S*(P1)**, ***S*(PA)**, ***S*(P1 + PA*x*)** and ***S*(P1/D)**, respectively.

Sol-to-gel transition and rheological measurements

The sol-to-gel transition behaviours of the IP formulations were investigated by a test-tube inverting method[7] and rheological measurements. The test-tube inverting method was carried out as follows. A vial containing an IP formulation was immersed in a water bath at the desired temperature for 15 min, removed from the water bath, then inverted repeatedly to determine the sol state or gel state based on the criteria of "flow" (= sol) and "no flow" (= gel) in 30 s, with a temperature increment of 1 °C per step. Measurements were repeated three times at each temperature.

The sol-to-gel transition behaviour of the IP formulations was also investigated by rheological measurements (temperature dependence and time course) using a dynamic rheometer (Thermo HAAKE RS600, Thermo Fisher Scientific, Waltham, MA, USA). A solvent trap was used for preventing vaporization of the solvent. The diameter of the parallel plate was 35 mm with a gap of 0.3 mm. The controlled stress and frequency were 4.0 dyn cm^{-2} and 1.0 rad s^{-1}, respectively. The heating rate was set at 0.5 °C min^{-1} for temperature dependence measurements. The storage modulus (G') and loss modulus (G'') of the formulations at 20–50 °C were observed and the gelation temperature (T_{gel}) was defined as the cross-over point of G' and G''. For investigation of mechanical properties after gelation for a longer time, the temperature was increased immediately from r.t. to 37 °C, and kept at 37 °C, while G' and G'' values were monitored for 15 h. To evaluate the reversibility (irreversibility) of the sol-to-gel transition of the samples, G' and G'' values were monitored upon temperature changes as follows. The sample was placed in the dynamic rheometer at 25 °C, and kept for 5 min. The temperature was then raised to 37 °C. After another 5 min, the temperature was decreased to 25 °C and the G' and G'' values were monitored for an additional 80 min

Determination of covalent gel fraction

To evaluate the covalent cross-linking formation in the hydrogel upon the temperature-responsive sol-to-gel transition, the amount of insoluble fraction against acetone (a good solvent for the copolymers and DPMP) was investigated for the ***S*(P1/D + PA40)** hydrogel. The IP formulation (300 μL) in a vial was heated to 37 °C and kept at 37 °C for 24 h. After that, an excess amount of acetone was added to dissolve the soluble polymers that were not covalently bonded, and then the supernatant was removed. This process was repeated 4 times. The obtained insoluble part was washed with pure water and acetone, and dried *in vacuo* before weighing. The insoluble fraction (%) was calculated by the following equation:

$$\text{Insoluble fraction } (\%) = W_{\text{ins}}/W_{\text{tot}} \times 100 \ (\%)$$

where W_{ins} is the weight of the insoluble part and W_{tot} is the total weight of tri-PCG-Acryl + DPMP.

Duration time of the gel state

In vitro duration times of gel states were investigated by immersion in PBS. The IP formulation (300 μL) was placed in a small vial (2.5 mL; diameter: 10 mm; height: 35 mm) and incubated at 37 °C for 1 min to prepare the hydrogel. The obtained hydrogel in the small vial without a cap was placed at the bottom of a larger (30 mL) sample tube with a flat bottom (diameter: 30 mm; height: 63 mm). PBS at 37 °C was added gently to the sample tube to immerse the hydrogel, and then the small vial was incubated at 37 °C. After removing the small vial from the sample tube and removing the supernatant, the sol or gel state of the sample in the small vial was determined by the test-tube inverting method. The weight of the remaining swelled hydrogel was also measured. The weight of the gel fraction (%) was calculated by the following equation:

$$\text{Weight of gel fraction } (\%) = (W_t/W_0) \times 100$$

where W_t is the weight of the gel fraction at each sampling time, and W_0 is the initial weight of the gel.

In vivo gelation, duration time of the gel state, and biocompatibility

In vivo gelation, the duration time of the gel state, and the biocompatibility of the IP formulations were investigated using

female SD rats. The IP formulation (25 wt%, 200 μL) was injected subcutaneously into the back of a rat using a syringe with a 25-gauge needle after anesthetizing the rat with isoflurane. The rats were sacrificed on certain days after injection, and the state of the hydrogel and tissue around the hydrogel was observed by the naked eye. Histological sections for the samples were developed by hematoxylin–eosin (HE) staining (Applied Medical Research Laboratory Co.). Moreover, a part of the residual hydrogel was removed from the injection sites for evaluation by rheological measurements. These animal experiments were accepted by the guidelines of animal experiments given at Kansai University. The ethical committee for animal experiments, Faculty of Chemistry, Materials and Bioengineering, Kansai University, approved the experiments.

Results and discussion

Synthesis of tri-PCG and tri-PCG-Acryl

Tri-PCGs were synthesized by ring-opening polymerization of CL and GL in the presence of PEG_{1500} as a macro-initiator using $Sn(Oct)_2$ as a catalyst. The MW of the PCGA segment and the total MW of tri-PCG-1 were 1900 and 5300 Da, respectively. Those of tri-PCG-2 were 1200 and 4000 Da, respectively. Detailed methods and characterization of the tri-PCGs are described in the ESI.†

Tri-PCG-Acryl was synthesized using tri-PCG-2 (Scheme 1). The ^{1}H-NMR spectra of tri-PCG-2 and tri-PCG-Acryl are shown in Fig. S1.† The degree of substitution of acryloyl groups per OH group was calculated to be 92% based on the ^{1}H-NMR spectrum. The results of SEC analysis before and after introduction of acryloyl groups are shown in Fig. S2.† Unimodal distributions were observed for tri-PCG-2 and tri-PCG-Acryl, and a slight increase in MW was observed in the SEC profiles upon the introduction of acryloyl groups. The obtained tri-PCG-1 and tri-PCG-Acryl were powders in the dry state at r.t. (Fig. S3†).

Sol-to-gel transition behaviour of the IP formulations

Before investigating covalent gelation, the temperature-responsive sol-to-gel transition behaviour of the copolymers, tri-PCG-1 and tri-PCG-Acryl, and their mixtures in PBS was investigated. The results are shown in the ESI (Table S3†). All of the samples with 25 wt% of total polymer concentration and any mixing ratios of tri-PCG-1/tri-PCG-Acryl showed sol-to-gel transitions at 32–34 °C. Therefore, subsequent experiments were carried out at the total copolymer concentration of 25 wt%, unless otherwise noted.

Next, we investigated the temperature-responsive sol-to-gel transition behaviour of the IP formulations and their reversibility (and irreversibility). Photographs of the sol-to-gel transitions for ***S*(P1 + PA$_{50}$)** and ***S*(P1/D + PA$_{50}$)** are shown in Fig. 1 as typical examples. ***S*(P1 + PA$_{50}$)** (1 : 1 mixture of tri-PCG-1 micelle solution and tri-PCG-Acryl micelle solution, without DPMP) showed gelation upon heating to 37 °C, and reverted to the sol state upon subsequent cooling to 4 °C: the sol-to-gel transition of ***S*(P1 + PA$_{50}$)** was reversible (Fig. 1(a)). ***S*(P1/D + PA$_{50}$)** prepared by mixing DPMP-loaded tri-PCG-1 micelle solution with tri-PCG-Acryl micelle solution was in the sol state just after mixing at r.t., and showed gelation upon heating to 37 °C. This sample was still in the gel state after cooling to 4 °C: the sol-to-gel transition of ***S*(P1/D + PA$_{50}$)** was irreversible (Fig. 1(b)). These results suggest that DPMP molecules in tri-PCG-1 micelles did not react with tri-PCG-Acryl in the other micelles upon mixing the solutions, but covalent bond formation between DPMP molecules and acryloyl groups of tri-PCG-Acryl molecules occurred only upon the temperature-responsive sol-to-gel transition. Photographs for controls (***S*(P1)**, ***S*(P1/D)** and ***S*(PA)**) and the other formulations having different tri-PCG-Acryl contents using similar procedures are shown in Fig. S7.† It was confirmed that irreversible gelation was observed only in the presence of both DPMP and tri-PCG-Acryl. We confirmed that ***S*(P1/D + PA$_{50}$)** was in the sol state for at least 2 h after mixing at r.t. This must be enough time for injection in the clinical setting. But ***S*(P1/D + PA$_{50}$)** gradually underwent gelation in 3 h at r.t. We found a solution for this matter, which will be reported in another paper.[40]

The sol-to-gel transition behaviour of the IP formulations was also investigated by rheological measurements. Typical results of the temperature-dependence of G' and G'' for the IP formulations (total copolymer concentration = 25 wt%) are shown in Fig. 2. Using these results, the sol-to-gel transition

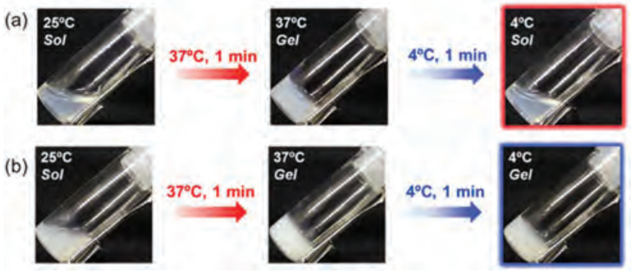

Fig. 1 Photographs of (a) ***S*(P1 + PA$_{50}$)** and (b) ***S*(P1/D + PA$_{50}$)** after preparation at 25 °C, subsequent heating at 37 °C for 1 min, and further cooling at 4 °C for 1 min. Total copolymer concentration = 25 wt%.

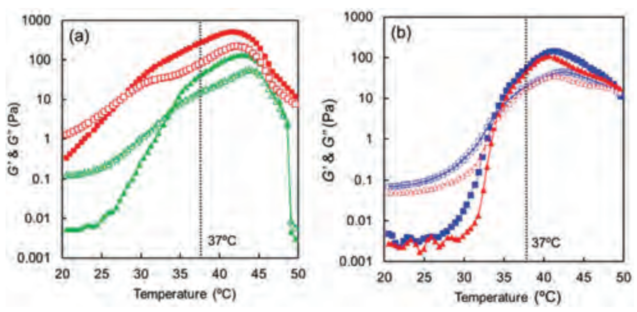

Fig. 2 Storage (G', closed symbols) and loss moduli (G'', open symbols) of (a) ***S*(P1/D + PA$_{50}$)** (■, □), ***S*(P1 + PA$_{50}$)** (▲, △) and (b) ***S*(P1 + PA$_{33}$)** (■, □), ***S*(P1 + PA$_{17}$)** (▲, △) as a function of temperature. Total copolymer concentration = 25 wt%.

This journal is © The Royal Society of Chemistry 2017

Table 1 Results of rheological studies

Code	T_{gel} (°C)	G' at 37 °C (Pa)	G'_{MAX} (Pa) ($T_{G'MAX}$[c] (°C))
**S(P1/D + PA$_{50}$)**[a]	27.8	233	510 (41.8)
**S(P1 + PA$_{50}$)**	33.8	39.1	136 (42.8)
**S(P1 + PA$_{33}$)**	34.3	46.1	143 (41.4)
**S(P1 + PA$_{17}$)**	34.3	36.0	108 (40.7)
**S(P1)**	34.3	32.4	74.2 (39.7)
**S(P1/D)**[b]	32.8	29.0	38.3 (40.2)
**S(PA)**	32.9	114	138 (37.7)

[a] Content of DPMP = 5.5 wt% for tri-PCG-1, [SH group] = [acryl group]. [b] Content of DPMP = 4.3 wt% for the total copolymer. [c] Temperature at which G' is maximum.

temperature, T_{gel}, can be estimated as the temperature at which G' overtakes G''. The T_{gel} values estimated from these measurements are summarized in Table 1. The results of rheological measurements for control samples are also shown in Fig. S8.† In Fig. 2(a), G' and G'' values for **S(P1/D + PA$_{50}$)** and **S(P1 + PA$_{50}$)** as functions of temperature are shown to compare the effects of DPMP. T_{gel} values for **S(P1/D + PA$_{50}$)** and **S(P1 + PA$_{50}$)** were 27.8 °C and 33.8 °C, respectively. These results suggest that the addition of DPMP (**D**) decreased T_{gel} values. In Fig. 2(b), G' and G'' values for formulations having different contents of tri-PCG-Acryl without DPMP, **S(P1 + PA$_{33}$)** and **S(P1 + PA$_{17}$)**, are shown. These samples showed almost the same T_{gel} values as **S(P1)**. These results indicate that the presence of tri-PCG-Acryl micelles in the formulation has almost no influence on the T_{gel} value in the range of less than 50%. The G' values at 37 °C and maximum values of G' (G'_{MAX}) at temperature $T_{G'MAX}$ of these formulations are also shown in Table 1. These G' values were relatively small (<510 Pa), but this does not matter much, because the G' values for covalent bond forming samples increased gradually after heating to 37 °C as discussed later.

The reversibility (or irreversibility) of the sol-to-gel transition of the IP formulations was also investigated by rheological studies. G' and G'' values for the IP formulations upon heating to 37 °C and subsequent cooling to 25 °C were recorded. The results for **S(P1/D + PA$_{50}$)** and **S(P1 + PA$_{50}$)** are shown in Fig. 3 as typical examples. The G' values for all IP formulations surpassed the G'' values immediately upon heating to 37 °C, indicating sol-to-gel transitions. After 5 min incubation at 37 °C, the samples were cooled to 25 °C. The G' values for **S(P1 + PA$_{50}$)** without DPMP decreased below the G'' values within 50 min after cooling to 25 °C, indicating gel-to-sol transitions (Fig. 3(b)). The sol-to-gel transitions for the IP formulations without DPMP were reversible. On the other hand, the G' values for **S(P1/D + PA$_{50}$)** remained higher than the G'' values for at least 90 min after cooling, maintaining the gel state (Fig. 3(a)); the sol-to-gel transitions for **S(P1/D + PA$_{50}$)** were irreversible. Other samples having different tri-PCG-Acryl contents also showed irreversible sol-to-gel transitions (Fig. S9†). These results indicated that covalent bond formation occurred in these samples upon the temperature-induced sol-to-gel transition and certain amounts of covalent cross-linking points were generated in the hydrogel network.

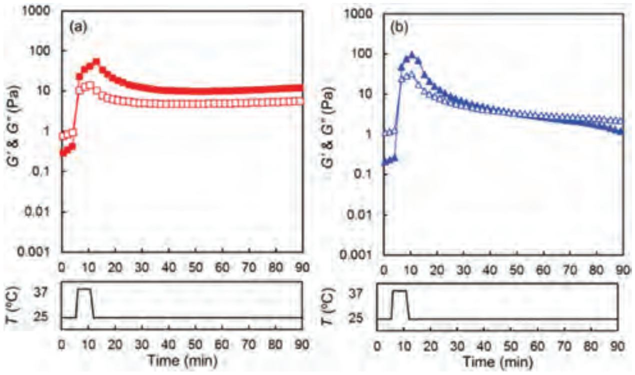

Fig. 3 Time course of the storage (G', closed symbols) and loss moduli (G'', open symbols) for (a) **S(P1/D + PA$_{50}$)**, (b) **S(P1 + PA$_{50}$)** after heating to 37 °C and subsequent cooling to 25 °C. Total copolymer concentration = 25 wt%. The temperature change schedule is shown at the bottom of each figure.

To confirm the formation of covalently cross-linked hydrogels in the system, we estimated the amount of the covalently cross-linked (insoluble) fraction in the hydrogel of **S(P1/D + PA$_{40}$)**. The insoluble fraction of **S(P1/D + PA$_{40}$)** against acetone and water after incubating at 37 °C for 24 hours was 84.8 ± 7.0 wt% in total weights of tri-PCG-Acryl + DPMP. The result indicated that a chemically cross-linked hydrogel was in fact formed for **S(P1/D + PA$_{40}$)** after physical gelation in the sol-to-gel-transition and further incubation at 37 °C. However, after just 1 min of heating at 37 °C, no insoluble fraction against acetone was found in **S(P1/D + PA$_{40}$)**. Therefore, the covalent bond formation reaction proceeded gradually. The progress of the thiol–ene reaction was also monitored by IR spectra measurements (see the ESI†).

We also investigated the time course of G' values of the IP formulations for a longer time (0–15 h) after heating to 37 °C and further maintaining at 37 °C. In Fig. 4, the results for **S(P1/D + PA$_{50}$)**, **S(P1/D + PA$_{36}$)**, **S(P1/D + PA$_{33}$)**, **S(P1/D + PA$_{16}$)** and **S(P1 + PA$_{50}$)** are shown as typical examples. The G' values after 15 h incubation at 37 °C for all formulations tested are summarized in Table 2. The G' value for **S(P1/D + PA$_{50}$)** was the

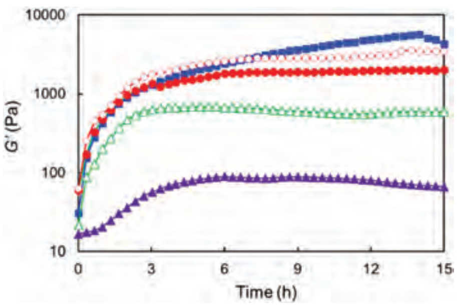

Fig. 4 Time course of the storage moduli (G') of **S(P1/D + PA$_{50}$)** (■), **S(P1/D + PA$_{36}$)** (○), **S(P1/D + PA$_{33}$)** (●), **S(P1/D + PA$_{16}$)** (△) and **S(P1 + PA$_{50}$)** (▲) after heating to 37 °C and further maintaining at 37 °C. Total copolymer concentration = 25 wt%.

Table 2 Storage moduli of the formulation after 15 h incubation at 37 °C

Code of formulation	G' after 15 h (Pa)
***S*(P1/D + PA$_{50}$)**	4235
***S*(P1/D + PA$_{40}$)**	4013
***S*(P1/D + PA$_{36}$)**	3477
***S*(P1/D + PA$_{33}$)**	2014
***S*(P1/D + PA$_{17}$)**	591
***S*(P1 + PA$_{50}$)**	530

highest, 4235 Pa, after 15 h among the formulations tested. G' values (after 15 h) are strongly dependent on the tri-PCG-Acryl contents in the formulations, and decreased with a decrease in tri-PCG-Acryl content. ***S*(P1/D + PA$_{17}$)** showed almost the same G' value as ***S*(P1 + PA$_{50}$)** without DPMP. The results suggest that 17 wt% of tri-PCG-Acryl could not form enough covalent cross-links (maybe below the percolation transition) to show high physical strength. These results indicate that the physical strength of the temperature-induced hydrogel could be easily controlled by changing the mixing ratio of tri-PCG-Acryl in the formulation. Moreover, the G' values observed in these thiol–ene covalent bond formation systems were much higher than those seen in our previous results with NH_2/OSu amide coupling systems,[35] which showed only 389 Pa after 400 min incubation at 37 °C (Fig. S12†). These results show that this system, where hydrophobic DPMP was entrapped in the micelle core, was more effective in providing physically strong hydrogels than our previous tri-PCG-OSu system with water-soluble polyamine in the aqueous phase.

Considering these results, we proposed a mechanism for the formation of covalent cross-links in ***S*(D/P1 + PA$_x$)** during the sol-to-gel transition as illustrated in Fig. 5. Below T_{gel}, the DPMP-loaded tri-PCG-1 micelles and tri-PCG-Acryl micelles exist separately in the mixed solution as flower-like micelles. The acryloyl groups on the tri-PCG-Acryl termini are located inside the hydrophobic micelle core, and are not in contact with the thiol groups of DPMP existing inside the tri-PCG-1 micelle core. With increasing temperature, dehydration of PEG segments and shrinking of the free volume of PEG occur to allow perturbation of the micelles. This phenomenon causes inter-micellar aggregation by hydrophobic interactions and formation of fibrous networks to provide a physically cross-linked hydrogel. During the aggregation process, the fusion of micelle cores occurred and then the thiol groups of DPMP in the tri-PCG-1 micelle core eventually reacted with acryloyl groups by the Michael-addition type thiol–ene reaction to form covalent cross-linking in the fibre-like micelle aggregates.

Duration time of the gel state and swelling behaviour

The duration time of the gel state for the formulations under highly wet conditions was investigated by soaking the formed hydrogels in PBS at 37 °C. In Fig. 6, the photographs for ***S*(P1/**

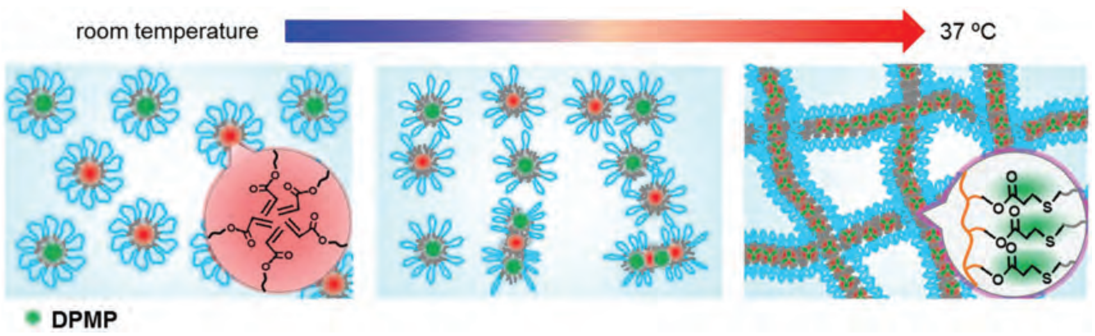

Fig. 5 Schematic illustration for the temperature-responsive irreversible sol-to-gel transition mechanism of ***S*(D/P1 + PA$_x$)** with increasing temperature from r.t. to body temperature.

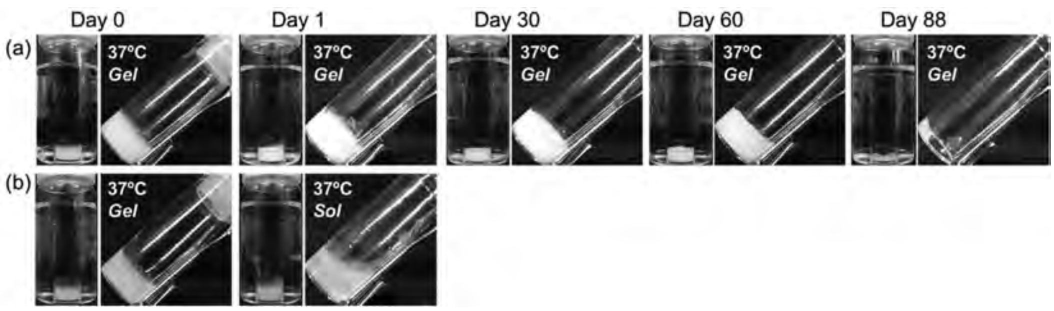

Fig. 6 Photographs of (a) ***S*(P1/D + PA$_{50}$)**, and (b) ***S*(P1 + PA$_{50}$)** after soaking in PBS for 0, 1, 30, 60, 88 days at 37 °C.

 This journal is © The Royal Society of Chemistry 2017

D + **PA$_{50}$**) and ***S*(P1** + **PA$_{50}$**) during the tests (days 1–88) are shown as typical examples. Fig. 7 shows the time course weight changes of the swelled hydrogels. ***S*(P1** + **PA$_{50}$**) became a sol state within 1 day during incubation in PBS at 37 °C (Fig. 6(b)). ***S*(P1/D)** and ***S*(P1)** were the same (data not shown). These results suggest that these physical gelation systems without chemical (covalent) cross-linking were in an equilibrium process, and the copolymers in the hydrogel dissociated and dissolved in PBS at the critical gelation concentration within 24 hours. On the other hand, ***S*(P1/D** + **PA$_{50}$**) maintained its gel state for 93 days, and finally disappeared after 94 days. Duration times of the gel state for ***S*(P1/D** + **PA$_{40}$**), ***S*(P1/D** + **PA$_{36}$**), ***S*(P1/D** + **PA$_{33}$**) and ***S*(P1/D** + **PA$_{17}$**) were 64, 37, 12 and 5 days, respectively (Fig. 8). These results indicated that the duration time of the gel state could be significantly extended by thiol–ene covalent bond formation more than our previous amide coupling (OSu/NH_2) system,[35] and could be easily controlled by changing the mixing ratio of tri-PCG-Acryl (17–50%).

After soaking in PBS, all of these IP formulations showed gradual swelling and subsequent rapid decrease in weights after reaching maximum swelling. These phenomena suggest gradual scission of the polymer chain and/or dissociation of cross-linking points due to degradation of PCGA segments. The maximum swelling ratios were in the range of 110–140%, and decreased with an increase in the mixing ratio of tri-PCG-Acryl. The gradual swelling and subsequent rapid decrease in weights must be due to the hydrolysis of the PCGA segments in tri-PCG-1 and tri-PCG-Acryl. To confirm this, we investigated the degradation behaviour of the tri-PCG-1 hydrogel (***S*(P1)**) soaked in PBS at 37 °C by monitoring the change in MW using SEC (Fig. S13†). The MW reduction of tri-PCG-1 in 30 days was confirmed, which supported the above hypothesis.

For further comparison of the relationships between the tri-PCG-Acryl content, the duration time of the gel state and the physical strength of the hydrogel, Fig. 8 summarizes the plots of G′ after 15 h (Table 2) and the duration time of the gel state (Fig. 7) *vs.* the content of tri-PCG-Acryl in ***S*(P1/D** + **PA$_x$**) systems. These two plots were similar and showed nearly sigmoidal curves with thresholds at 30–40%. This relationship suggested that the percolation thresholds in this system are in the range of 30–40% of tri-PCG-Acryl (with an equivalent amount of DPMP) and have a great influence on the physical strength and duration time of the gel state.

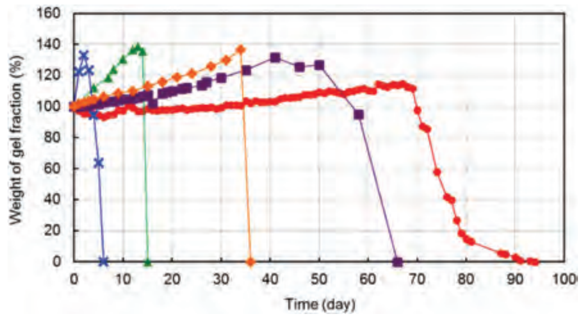

Fig. 7 Swelling and erosion profiles of *S*(P1/D + PA$_{50}$) (●), *S*(P1/D + PA$_{40}$) (■), *S*(P1/D + PA$_{36}$) (◆), *S*(P1/D + PA$_{33}$) (▲) and *S*(P1/D + PA$_{17}$) (☒) hydrogels soaked in PBS at 37 °C.

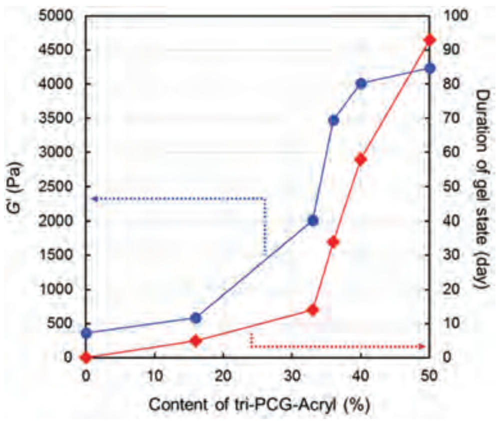

Fig. 8 Plots of *G*′ after 15 h and duration time of the gel state *vs.* content of tri-PCG-Acryl in *S*(P1/D + PA$_x$) systems. ●: *G*′ after heating to 37 °C and further maintaining at 37 °C for 15 h; ◆: duration time of the gel state soaked in PBS at 37 °C.

In vivo compatibility

To evaluate the *in vivo* biocompatibility, degradation, and duration time of the gel state, the state of the hydrogels after subcutaneous (s.c.) injection into SD rats was investigated for ***S*(P1/D** + **PA$_{40}$**), ***S*(P1/D** + **PA$_{36}$**), ***S*(P1/D** + **PA$_{33}$**), ***S*(P1/D** + **PA$_{17}$**) and ***S*(P1)**. Fig. 9 shows the photographs of the hydrogels of ***S*(P1/D** + **PA$_{40}$**) 1, 8, 30 and 68 days after s.c. injection as representative data. The hydrogel still existed in the gel state even after 68 days at the injected site, and no significant inflammation and angiogenesis were observed. The size of the hydrogel tended to decrease along with the implantation period. The remaining sample was confirmed to be a covalently cross-linked hydrogel by the fact that the sample (after 30 days) was insoluble against acetone as a good solvent for the copolymers (Fig. S14†). In Fig. 10, the photographs of the other samples, ***S*(P1)**, ***S*(P1/D** + **PA$_{36}$**), ***S*(P1/D** + **PA$_{33}$**), ***S*(P1/D** + **PA$_{17}$**) and ***D*(P1/D** + **PA$_{40}$**), on days 1 and 8 after s.c. injection are shown.

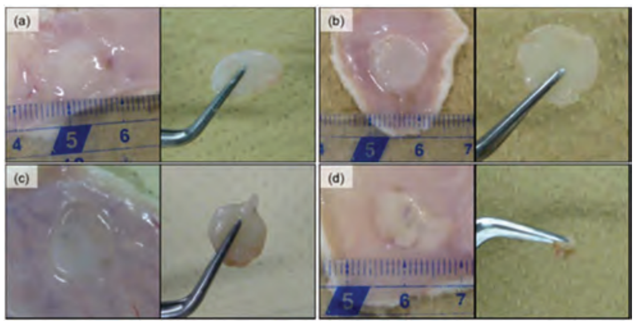

Fig. 9 Photographs of *S*(P1/D + PA$_{40}$) hydrogels in rats on days (a) 1, (b) 8, (c) 30 and (d) 68 after subcutaneous injection.

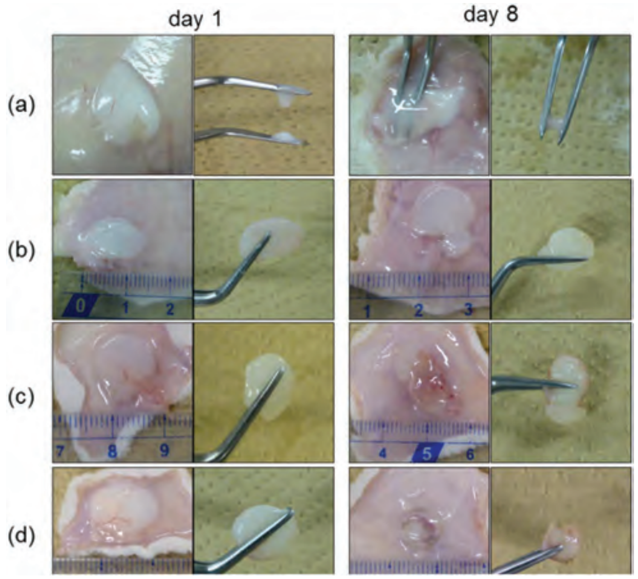

Fig. 10 Photographs of (a) ***S*(P1)**, (b) ***S*(P1/D + PA$_{36}$)**, (c) ***S*(P1/D + PA$_{33}$)**, and (d) ***S*(P1/D + PA$_{17}$)** hydrogels in rats on days 1 and 8 after subcutaneous injection.

Significant inflammation and angiogenesis were also not observed. As seen in Fig. 10(a), the ***S*(P1)** sample existed after 8 days, but was in the sol state with lower viscosity on days 1 and 8. All of the other samples, ***S*(P1/D + PA$_{36}$)**, ***S*(P1/D + PA$_{33}$)** and ***S*(P1/D + PA$_{17}$)**, existed in gel states after 8 days. The size of the ***S*(P1/D + PA$_{17}$)** hydrogel with a lower content of tri-PCG-Acryl (17%) was relatively small compared with those having a higher content of tri-PCG-Acryl, ***S*(P1/D + PA$_{36}$)** and ***S*(P1/D + PA$_{33}$)**.

To evaluate the state (sol or gel) and physical strength of the hydrogels after s.c. injection, the samples were recovered from the injected site and investigated by rheological measurements. Fig. 11 shows the time course G' values of these hydrogels recovered from s.c. spaces of rats. G' and G'' values for each sample are shown in Table 3. ***S*(P1)** showed G' values less than 10 Pa, which were lower than G'' on day 1, meaning the hydrogel reverted to the sol state about 1 day after s.c. injection. The samples of ***S*(P1/D + PA$_{33}$)** and ***S*(P1/D + PA$_{17}$)** could not be recovered from the injected site on days 40 and 20, respectively, likely due to degradation and reversion to the sol state. All of the samples that could be recovered from the injected sites were in the gel state ($G' > G''$). G' values of ***S*(P1/D + PA$_{40}$)** were maintained over 2900 Pa for 30 days, and became 160 Pa after 68 days. ***S*(P1/D + PA$_{36}$)** had G' values over 1900 for 14 days. There was an obvious tendency for the samples with higher content of tri-PCG-Acryl to show higher G' values for longer times.

Table 3 Storage and loss moduli of the IP formulation after *in vivo* injection

Code	Time after injection (day)	G' (Pa)	G'' (Pa)
***S*(P1/D + PA$_{40}$)**	1	3615	535
	8	3288	412
	30	2902	446
	68	160	28.9
***S*(P1/D + PA$_{36}$)**	1	3087	376
	8	2430	255
	14	1956	241
	30	749	137
	60	55	13.7
***S*(P1/D + PA$_{33}$)**	1	2410	359
	8	2450	375
	14	683	129
	30	26.4	4.5
***S*(P1/D + PA$_{17}$)**	1	127	26.3
	8	63.6	16
	14	111	38.4
***S*(P1)**	1	10	11.1
	8	0.002	0.01

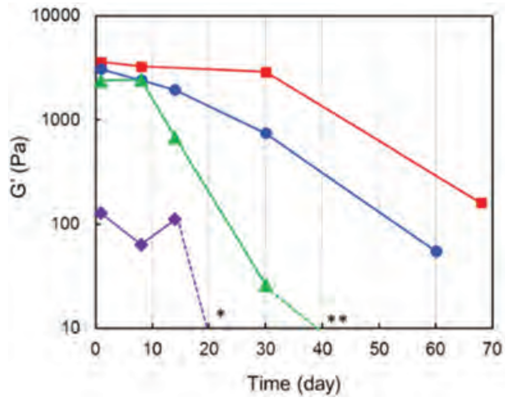

Fig. 11 Time course of the storage modulus (G') of ***S*(P1/D + PA$_{40}$)** (■), ***S*(P1/D + PA$_{36}$)** (●), ***S*(P1/D + PA$_{33}$)** (▲), and ***S*(P1/D + PA$_{17}$)** (◆) hydrogels in rats after subcutaneous injection. *,** Impossible to recover on days 20 and 40, respectively.

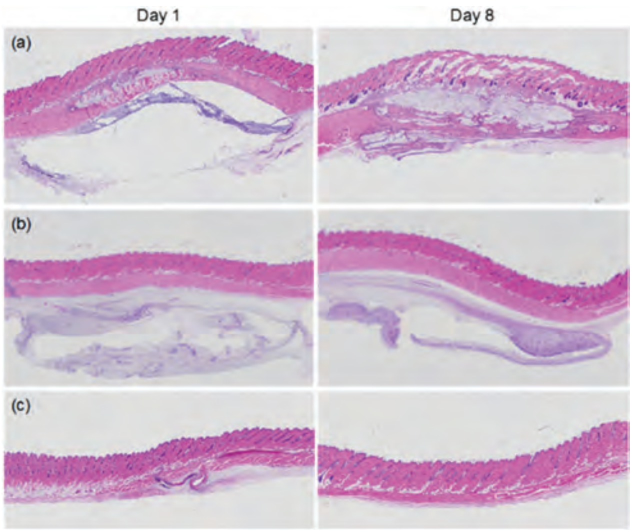

Fig. 12 Results of histological analysis for the samples after subcutaneous injection into rats on days 1 and 8 after injection. (a) ***S*(P1/D + PA$_{40}$)**, (b) ***S*(P1)** and (c) PBS.

This journal is © The Royal Society of Chemistry 2017

All of these results indicate that covalent bond formation in the ***S***(**P1**/**D** + $\mathbf{PA}_x$) systems occurred *in vivo*, and it was effective to achieve significantly longer duration times of the gel state in the s.c. space. Moreover, the *in vivo* duration time of the gel state can also be controlled by changing the content of tri-PCG-Acryl and DPMP, displaying good agreement with the *in vitro* results.

We carried out histological analysis after HE staining for ***S***(**P1**/**D** + $\mathbf{PA}_{40}$) and ***S***(**P1**) around the injected tissue, and the results are shown in Fig. 12. No immunological response was observed in any samples. Although slight nonspecific granulation and fibrosis were observed on day 8 for both ***S***(**P1**/**D** + $\mathbf{PA}_{40}$) and ***S***(**P1**), these were in the normal level in the wound healing process, and severe problems with the histocompatibility of the samples were not observed.

Conclusions

In this study, we successfully prepared a biodegradable IP system exhibiting a temperature-responsive irreversible sol-to-gel transition by covalent bond formation *via* bio-orthogonal Michael-addition type thiol–ene reaction by mixing solutions of tri-PCG/DPMP and tri-PCG-Acryl. This is a unique gelation system in that the covalent bond formation reaction is triggered by a temperature increase. The obtained hydrogel showed a significantly longer duration time of the gel state and higher physical strength *in vitro* and *in vivo* compared with previous non-covalent physical gelation systems and our temperature-triggered covalent gelation system using an amide coupling reaction of OSu and NH_2.[35] The physical strength and duration time of the gel state of the obtained hydrogels could be easily controlled by a simple method, *i.e.*, by just changing the mixing ratio of tri-PCG/DPMP and tri-PCG-Acryl solutions. The duration time of the gel state could be altered in the range of 1 day to 93 days *in vitro* (or 1 day to more than 60 days after s.c. implantation *in vivo*), which was much wider than in our previous report (1–12 days), and the G' value (after 15 h) of the hydrogel can be altered from 500 Pa to 4000 Pa.

This biodegradable IP system with a longer and controllable duration time of the gel state has great advantages as an implantable biomaterial. As mentioned in the Introduction, the sustained release of drugs from the hydrogel during the desired period can be expected. The IP hydrogel systems with a wide variety of degradation rates and moduli can also provide suitable scaffolds for the regeneration of different tissues. Particularly, the modulus of the hydrogel can also be controlled. It might affect the cell fate in a 3D culture system or the patient compliance during *in vivo* injection. So, this system will provide some new information on these matters. Moreover, the differentiation behaviour of stem cells strongly depends on the moduli of the scaffold.[41] This system may have the potential as scaffolds for differentiation of stem cells to the specific directions. As an anti-adhesive material, a very long duration time (more than 2 weeks) may not be needed. But, there has been no report of a systematic study on the ideal duration period for anti-adhesive materials. By controlling the degradation period, the information on the ideal duration for anti-adhesive materials can be obtained. Therefore, this developed IP system is an excellent candidate for drug releasing depots, scaffolds for regenerative medicine, and adhesion prevention materials.

Conflict of interest

The authors declare no competing financial interest.

Acknowledgements

This work was financially supported in part by the Private University Research Branding Project: Matching Fund Subsidy from the Ministry of Education, Culture, Sports, Science and Technology (MEXT) Japan (2016–2020), a Grant-in-Aid for Scientific Research (16H01854) from the Japan Society for the Promotion of Science (JSPS), a Grant-in-Aid for JSPS Fellows (16J02102), and the Kansai University Outlay Support for Establishing Research Centers, 2016. The authors thank SC Organic Chemical Co. Ltd for providing DPMP.

Notes and references

1 M. H. Park, M. K. Joo, B. G. Choi and B. Jeong, *Acc. Chem. Res.*, 2012, **45**, 424.
2 H. J. Moon, D. Y. Ko, M. H. Park, M. K. Joo and B. Jeong, *Chem. Soc. Rev.*, 2012, **41**, 4860.
3 K. Nagahama, A. Takahashi and Y. Ohya, *React. Funct. Polym.*, 2013, **73**, 979.
4 M. K. Nguyen and D. S. Lee, *Macromol. Biosci.*, 2010, **10**, 563.
5 L. Yu and J. Ding, *Chem. Soc. Rev.*, 2008, **37**, 1473.
6 B. Yeon, M. H. Park, H. J. Moon, S. J. Kim, Y. W. Cheon and B. Jeong, *Biomacromolecules*, 2013, **14**, 3256.
7 K. Nagahama, T. Ouchi and Y. Ohya, *Adv. Funct. Mater.*, 2008, **18**, 1220.
8 N. Oyama, H. Minami, D. Kawano, M. Miyazaki, T. Maeda, K. Toma, A. Hotta and K. Nagahama, *Biomater. Sci.*, 2014, **2**, 1057.
9 S. Choi, M. Baudys and S. W. Kim, *Pharm. Res.*, 2004, **21**, 827.
10 K. Manokruang and D. S. Lee, *Macromol. Biosci.*, 2013, **13**, 1195.
11 K. Li, L. Yu, X. Liu, C. Chen, Q. Chen and J. Ding, *Biomaterials*, 2013, **34**, 2834.
12 Z. Zhang, J. Ni, L. Chen, L. Yu, J. Xu and J. Ding, *Biomaterials*, 2011, **32**, 4725.
13 L. Yu, H. Hu, L. Chen, X. Bao, Y. Li, L. Chen, G. Xu, X. Ye and J. Ding, *Biomater. Sci.*, 2014, **2**, 1100.
14 J. H. Hong, J. W. Choe, G. Y. Kwon, D. Y. Cho, D. S. Sohn, S. W. Kim, Y. C. Woo, C. J. Lee and H. Kang, *J. Surg. Res.*, 2011, **166**, 206.

15 P. Kan, X. Z. Lin, M. F. Hsieh and K. Y. Chang, *J. Biomed. Mater. Res., Part B*, 2005, **75**, 185.

16 X. Chen, L. Huang, H. J. Sun, S. Z. D. Cheng, M. Zhu and G. Yang, *Macromol. Rapid Commun.*, 2014, **35**, 579.

17 L. Weng, N. Rostambeigi, N. D. Zantek, P. Rostamzadeh, M. Bravo, J. Carey and J. Golzarian, *Acta Biomater.*, 2013, **9**, 8182.

18 L. Yu, W. Xu, W. Shen, L. Cao, Y. Liu, Z. Li and J. Ding, *Acta Biomater.*, 2014, **10**, 1251.

19 C. Chen, L. Chen, L. Cao, W. Shen, L. Yu and J. Ding, *RSC Adv.*, 2014, **4**, 8789.

20 M. J. Hwang, J. M. Suh, Y. H. Bae, S. W. Kim and B. Jeong, *Biomacromolecules*, 2005, **6**, 885.

21 S. J. Bae, J. M. Suh, Y. S. Sohn, Y. H. Bae, S. W. Kim and B. Jeong, *Macromolecules*, 2005, **38**, 5260.

22 S. J. Bae, M. K. Joo, Y. Jeong, S. W. Kim, W. K. Lee, Y. S. Sohn and B. Jeong, *Macromolecules*, 2006, **39**, 4873.

23 C. B. Liu, C. Y. Gong, M. J. Huang, J. W. Wang, Y. F. Pan, Y. D. Zhang, G. Z. Li, M. L. Gou, K. Wang, M. J. Tu, Y. Q. Wei and Z. Y. Aian, *J. Biomed. Mater. Res., Part B*, 2008, **84**, 164.

24 Z. Jiang, Y. You, Q. Gu, J. Hao and X. Deng, *Macromol. Rapid Commun.*, 2008, **29**, 1264.

25 Y. Yoshida, A. Takahashi, A. Kuzuya and Y. Ohya, *Polym. J.*, 2014, **46**, 632.

26 E. A. Phelps, N. O. Enemchukwu, V. F. Fiore, J. C. Sy, N. Murthy, T. A. Sulchek, T. H. Barker and A. J. Garcia, *Adv. Mater.*, 2012, **24**, 64.

27 C. A. DeForest, B. D. Polizzotti and K. S. Anseth, *Nat. Mater.*, 2009, **8**, 659.

28 F. Yu, X. Cao, Y. Li, L. Zeng, B. Yuan and X. Chen, *Polym. Chem.*, 2014, **5**, 1082.

29 F. Wang, Z. Li, M. Khan, K. Tamama, P. Kuppusamy, W. R. Wagner, C. K. Sen and J. Guan, *Acta Biomater.*, 2010, **6**, 1978.

30 M. Kurakazu, T. Katashima, M. Chijiishi, K. Nishi, Y. Akagi, T. Matsunaga, M. Shibayama, U. Chung and T. Sakai, *Macromolecules*, 2010, **43**, 3935.

31 C. B. Liu, C. Y. Gong, M. J. Huang, J. W. Wang, Y. F. Pan, Y. D. Zhang, G. Z. Li, M. L. Gou, K. Wang, M. J. Tu, Y. Q. Wei and Z. Y. Aian, *J. Biomed. Mater. Res., Part B*, 2008, **84**, 165.

32 T. Fujiwara, T. Mukose, T. Yamaoka, H. Yamane, S. Sakurai and Y. Kimura, *Macromol. Biosci.*, 2001, **1**, 204.

33 T. Mukose, T. Fujiwara, J. Nakano, I. Taniguchi, M. Miyamoto, Y. Kimura, I. Teraoka and C. W. Lee, *Macromol. Biosci.*, 2004, **4**, 361.

34 K. Nagahama, K. Fujiura, S. Enami, T. Ouchi and Y. Ohya, *J. Polym. Sci., Part A: Polym. Chem.*, 2008, **46**, 6317.

35 Y. Yoshida, K. Kawahara, K. Inamoto, S. Mitsumune, S. Ichikawa, A. Kuzuya and Y. Ohya, *ACS Biomater. Sci. Eng.*, 2017, **3**, 56.

36 D. P. Nair, M. Podgorski, S. Chatani, T. Gong, W. Xi, C. R. Fenoli and C. N. Bowman, *Chem. Mater.*, 2014, **26**, 724.

37 C. E. Hoyle, T. Y. Lee and T. Roper, *J. Polym. Sci., Part A: Polym. Chem.*, 2004, **42**, 5301.

38 B. Lowe, *Polym. Chem.*, 2010, **1**, 17.

39 C. E. Hoyle and C. N. Bowman, *Angew. Chem., Int. Ed.*, 2010, **49**, 1540.

40 Y. Yoshida, K. Takata, H. Takai, K. Kawahara, A. Kuzuya, Y. Ohya, *et al.*, *J. Biomater. Sci., Polym. Ed.*, in press.

41 A. J. Engler, S. Sen, H. L. Sweeney and D. E. Discher, *Cell*, 2006, **126**, 677.

This journal is © The Royal Society of Chemistry 2017

STEM CELLS TRANSLATIONAL MEDICINE

TISSUE-SPECIFIC PROGENITOR AND STEM CELLS

Cardiac Adipose-Derived Stem Cells Exhibit High Differentiation Potential to Cardiovascular Cells in C57BL/6 Mice

HIROKI NAGATA,[a,b] MASAAKI II,[a] EIKO KOHBAYASHI,[b] MASAAKI HOSHIGA,[c] TOSHIAKI HANAFUSA,[b] MICHIO ASAHI[a]

Key Words. Cardiac • Differentiation • Adipose stem cells • Stem cell transplantation

Departments of [a]Pharmacology, [b]Internal Medicine (I), and [c]Internal Medicine (III), Faculty of Medicine, Osaka Medical College, Osaka, Japan

Correspondence: Masaaki Ii, M.D., Ph.D., Department of Pharmacology, Faculty of Medicine, Osaka Medical College, 2-7, Daigaku-machi, Takatsuki, Osaka 569-8686, Japan. Telephone: 81-72-683-1221; E-Mail: masaii@osaka-med.ac.jp

Received April 25, 2015; accepted for publication October 15, 2015; published Online First on December 18, 2015.

©AlphaMed Press

1066-5099/2015/$20.00/0

http://dx.doi.org/10.5966/sctm.2015-0083

ABSTRACT

Adipose-derived stem cells (AdSCs) have recently been shown to differentiate into cardiovascular lineage cells. However, little is known about the fat tissue origin-dependent differences in AdSC function and differentiation potential. AdSC-rich cells were isolated from subcutaneous, visceral, cardiac (CA), and subscapular adipose tissue from mice and their characteristics analyzed. After four different AdSC types were cultured with specific differentiation medium, immunocytochemical analysis was performed for the assessment of differentiation into cardiovascular cells. We then examined the in vitro differentiation capacity and therapeutic potential of AdSCs in ischemic myocardium using a mouse myocardial infarction model. The cell density and proliferation activity of CA-derived AdSCs were significantly increased compared with the other adipose tissue-derived AdSCs. Immunocytochemistry showed that CA-derived AdSCs had the highest appearance rates of markers for endothelial cells, vascular smooth muscle cells, and cardiomyocytes among the AdSCs. Systemic transfusion of CA-derived AdSCs exhibited the highest cardiac functional recovery after myocardial infarction and the high frequency of the recruitment to ischemic myocardium. Moreover, long-term follow-up of the recruited CA-derived AdSCs frequently expressed cardiovascular cell markers compared with the other adipose tissue-derived AdSCs. Cardiac adipose tissue could be an ideal source for isolation of therapeutically effective AdSCs for cardiac regeneration in ischemic heart diseases. STEM CELLS TRANSLATIONAL MEDICINE *2016;5:141–151*

SIGNIFICANCE

The present study found that cardiac adipose-derived stem cells have a high potential to differentiate into cardiovascular lineage cells (i.e., cardiomyocytes, endothelial cells, and vascular smooth muscle cells) compared with stem cells derived from other adipose tissue such as subcutaneous, visceral, and subscapular adipose tissue. Notably, only a small number of supracardiac adipose-derived stem cells that were systemically transplanted sufficiently improved cardiac functional recovery after myocardial infarction, differentiating into cardiovascular cells in the ischemic myocardium. These findings suggest a new autologous stem cell therapy for patients with myocardial ischemia, especially those with secondary myocardial ischemia after cardiovascular open chest surgery.

INTRODUCTION

Heart failure resulting from ischemic heart disease is one of the leading causes of hospitalization and reduced quality of life for patients worldwide [1]. Although advances in the treatment of patients with myocardial infarction (MI), such as the development of devices for catheterization, including drug-eluting stents, have significantly improved the outcomes, a need for novel therapies remains to reduce mortality further and prevent the deleterious consequences that result from myocardial damage. At present, the only effective treatment for advanced heart failure that can fully restore cardiac function is heart transplantation. However, only a limited number of patients will be able to receive the therapy owing to the difficulty of finding appropriate donors and/or legal restrictions in each country. Recently, numerous investigators have evaluated the application of stem/progenitor cells for the treatment of ischemic heart diseases, including MI, in both animal experiments and human clinical trials, and favorable outcomes have been demonstrated. The major stem/progenitor cell sources for human autologous cell transplantation therapy have been reported to be peripheral blood [2–4] and bone marrow [4–7] in ongoing clinical trials for ischemic

 ©AlphaMed Press 2016

heart diseases. However, each of the currently used adult stem cell reservoirs has practical limitations that obstruct their widespread use in treating human disease owing to the requirement of invasive harvesting procedures or ex vivo manipulation/ expansion techniques.

Adipose tissue consists of mature adipocytes and a mononuclear cell fraction termed stromal vascular fraction (SVF). The SVF is a diverse mixture of cells, including endothelial cells (ECs), vascular smooth muscle cells (VSMCs), blood cells, and mesenchymal stem cells (MSCs), that is identical to adipose tissue-derived stem cells (AdSCs). AdSCs have phenotypic and functional properties (i.e., multilineage differentiation potential) similar to those of bone marrow-derived MSCs. Of particular relevance, AdSCs have been reported to differentiate, not only into adipocytes [8], osteoblasts [8, 9], chondrocytes [10, 11], pancreatic β-cells [12], hepatocytes [13], neural cells [14, 15], and myocytes [8], but also into cardiovascular lineage cells, such as cardiomyocytes (CMs) [16], ECs [9, 17], and VSMCs [18]. Most importantly for their clinical application, AdSC-enriched SVFs can be isolated in large quantities by minimally invasive liposuction, with a significantly higher yield of progenitor cells per volume compared with bone marrow. The SVF of adipose tissue has the potential to improve cardiac function after MI by several mechanisms: delivery of cardiovascular cell replacement, salvage of host cardiomyocytes through antiapoptotic mechanisms, or stimulation of angiogenesis. Similar to bone marrow-derived MSCs, AdSC-enriched SVFs secrete a number of paracrine factors that are proangiogenic [19], anti-inflammatory [20, 21], and chemoattractive for stem/ progenitor cells [19] and demonstrate their beneficial effects on jeopardized myocardium by ischemic insult. Early clinical trials of autologous AdSC-enriched SVF transplantation therapy for MI patients are now ongoing in Europe and have shown significant improvement in cardiac function [22]. However, the favorable effect of transplanted SVF has been attributed to the paracrine effect rather than a direct contribution of AdSCs to tissue regeneration via transdifferentiation into cardiovascular lineage cells, as we reported previously [19]. To use AdSCs as a tool for myocardial tissue regeneration therapy, the frequency of AdSC transdifferentiation into cardiovascular cells should be increased either by manipulation of the inducible culture method or selection of appropriate adipose tissue to isolate AdSCs that include a cell population with a high differentiation potential for cardiovascular cells.

In clinical settings, autologous AdSC-enriched SVF transplantation has already been performed in mammoplasty after mastectomy to treat breast cancer [23, 24] and in breast implants in plastic surgery [25], with favorable outcomes. Because of the simple surgical procedure, subcutaneous adipose tissue collected by liposuction has been frequently used for AdSC isolation in most cases. However, no investigation has proved that, of the different organ-derived adipose tissues, subcutaneous adipose tissue is the best source of AdSCs for the treatment of cardiovascular diseases. We therefore tested the hypothesis that AdSCs derived from cardiac adipose tissue might exhibit high transdifferentiation potential to cardiovascular lineage cells compared with a variety of organ-derived adipose tissues. In the present study, mouse AdSCs were isolated from four different adipose tissues of subcutaneous, visceral, subscapular, and cardiac fat tissues and their stem/progenitor characteristics as a source of cardiovascular tissue regeneration in vitro and in vivo were examined.

MATERIALS AND METHODS

Adipose Tissue Harvesting and AdSC Isolation

The institutional animal care and use committee of Osaka Medical College approved all the following research protocols (approval ID, 22030), including surgical procedures and animal care. C57BL/ 6N (Shimizu Laboratory Supplies, Kyoto, Japan, http://www.shimizu-ls.co.jp) and B6.129S7-Gt(ROSA)26Sor/J (The Jackson Laboratory, Bar Harbor, ME, http://www.jax.com) male mice (aged 16–20 weeks) were sacrificed under anesthesia with pentobarbital (200 mg/kg i.p.). Adipose tissue was harvested from inguinal, abdominal, supracardiac (para-aortic root), and subscapular regions and used in all experiments as subcutaneous white adipose tissue (SC), visceral white adipose tissue (VL), cardiac brown adipose tissue (CA), and subscapular brown adipose tissue (SS), respectively.

AdSCs were isolated from each adipose tissue as previously described with minor modifications [26]. In brief, adipose tissue was washed in phosphate-buffered saline (PBS) and minced, followed by digestion in 5 ml of type I collagenase (1 mg/ml in 1% bovine serum albumin [BSA]/Hanks' balanced saline solution; Life Technologies Japan, Tokyo, Japan, http://www.lifetechnologies.com) for 40 minutes at 37°C using a gentleMACS Dissociator (Miltenyi Biotec K.K., Tokyo, Japan, http://www.miltenyibiotec.com) according to the manufacturer's instructions. The digested tissue was filtered through a 40-μm cell strainer (BD Falcon, Tokyo, Japan, http://www.bdbiosciences.com) and centrifuged at 450*g* for 10 minutes. The supernatant containing adipocytes and debris was discarded. Pelleted cells were suspended with 5 mmol/l EDTA/PBS and layered over an equal volume of 1.083 g/ml Histopaque 1083 solution (Sigma-Aldrich Japan K.K., Tokyo, Japan, http://www.sigmaaldrich.com). After centrifugation at 900*g* for 30 minutes, mononuclear cells (MNCs) were collected from the gradient interface, and the number of trypan blue-unstained cells sized 5–30 μm was measured by a conventional cytometer (LUNA; Logos Biosystems, Inc., Annandale, VA). The MNCs were used as a freshly isolated AdSC-containing SVF for the experiments. Because the number of MNCs varies depending on the tissue volume, the density of MNCs in each adipose tissue was calculated by dividing the absolute number of MNCs by the weight of the tissues, and the AdSC-rich cellularity was assessed.

AdSC Culture for Differentiation to Cardiovascular Cells

Fleshly isolated AdSCs were cultured in 10% fetal bovine serum (FBS)/Dulbecco's modified Eagle's medium (DMEM)-F12 containing antibiotics on plastic dishes at a density of 10^4/cm^2 under conditions of 5% CO_2 and 37°C. After 7 days in culture, adherent cells (AdSCs) were harvested by trypsinization for 5 minutes at 37°C and pipetting. For expansion, the cells were further cultured in MesenPRO RS medium (Life Technologies Japan) at a density of 5×10^3 per cm^2 under 5% O_2 and 37°C conditions for 5 days. The adherent AdSCs were then cultured for cardiovascular differentiation under specific culture conditions, as previously described, with minor modifications. In brief, the adherent AdSCs were cultured under conditions of 5% CO_2 and 37°C in (a) 10% FBS/DMEM supplemented with transforming growth factor-β (2 ng/ml) for vascular smooth muscle cell differentiation [18, 27]; (b) 2% FBS/DMEM supplemented with EGM-2 BulletKit containing human fibroblast growth factor, human vascular endothelial growth factor, human insulin-like growth factor, ascorbic acid, human epidermal growth factor, heparin, and insulin transferrin

for endothelial differentiation [17, 28]; and (c) 10% FBS/DMEM-F12 supplemented with phorbol myristate acetate (2 nmol/l) for 24 hours, followed by MethoCult medium (StemCell Technologies Inc., Vancouver, BC, Canada, http://www.stemcell.com) for cardiomyocyte differentiation for 7 days [16, 29]. The cells were fixed with 2% paraformaldehyde (PFA)/PBS for 10 minutes at room temperature (RT), followed by PBS washing, and examined under a fluorescence microscope (model BZ-8000; Keyence, Osaka, Japan, http://www.keyence.com) after immunofluorescent staining.

Cell Proliferation Assay

The adherent AdSCs (5×10^4 cells per well) were seeded on 8-well chamber glass slides (Nalgene Nunc, Rochester, NY, http://www.thermoscientific.com) cultured in MesenPRO RS medium (Life Technologies Japan) in the presence of 5-bromo-2′-deoxyuridine (BrdU; 10 μmol/l; Sigma-Aldrich Japan K.K.) for 24 hours at 37°C under a 5% O_2 condition. After immunocytostaining with anti-BrdU antibody (1:100; BD Pharmingen, San Diego, CA, http://www.bdbiosciences.com) as described below, the BrdU-positive cells in each chamber were counted at five different high power fields (HPFs; $\times$200). Proliferation activity was evaluated using the BrdU labeling index calculated as a BrdU-positive percentage to the total cell number.

Fluorescent Immunocytochemistry for AdSC Differentiation Assay

The adherent cells were fixed with 2% PFA/PBS for 10 minutes at RT, followed by PBS washing, and permeabilized by incubation with 0.1% Triton X-100/PBS solution for 5 minutes at RT. The samples were blocked in antibody dilution buffer, 2% BSA/PBS, for 1 hour at RT. After removal of the blocking solution, primary antibodies/markers were added: anti-CD31 (1:100; Abcam, Cambridge, MA, http://www.abcam.com) and fluorescein-labeled griffonia simplicifolia lectin 1, isolectin B4 (Vector Laboratories, Burlingame, CA, http://www.vectorlabs.com) for ECs; anti-SM22 antibody (1:100; Abcam) and anti-calponin (1:200; Abcam) for VSMCs; and GATA4 (Santa Cruz Biotechnology, Santa Cruz, CA, http://www.scbt.com) and cardiac troponin T (Thermo Fisher Scientific, Fremont, CA, http://www.thermofisher.com) for CMs in antibody dilution buffer at 4°C overnight. After washing with PBS, the cells were incubated with secondary antibodies prepared at 1:500 in antibody dilution buffer: Alexa Fluor 488 donkey anti-goat IgG, Alexa Fluor 488 goat anti-rabbit IgG, and Alexa Fluor 488 goat anti-rat IgG (Jackson ImmunoResearch Laboratories, Inc., West Grove, PA, http://www.jacksonimmuno.com) for 30 minutes at RT. After the secondary antibodies were removed and the cells had been washed with PBS, nuclear counter staining was performed by incubation with 4′,6-diamidino-2-phenylindole (DAPI) solution (1 μg/ml in PBS; Sigma-Aldrich Japan K.K.) for 10 minutes at RT. The sample slides were covered by a coverslip with mounting medium (ImmunoBioScience, Mukilteo, WA, http://www.immunobioscience.com), followed by sealing with nail varnish before evaluation under a fluorescence microscope (model BZ8000; Keyence). The antigen (marker for differentiation)-positive cells in each chamber were counted in five different HPFs ($\times$200), and differentiation activity was evaluated by comparing the antigen-positive cell percentage to the total DAPI-positive cell number.

Fluorescence-Activated Cell Sorting Analysis

Fleshly isolated AdSCs were incubated with 0.5% BSA in PBS containing fluorescence (allophycocyanin [APC]/fluorescein

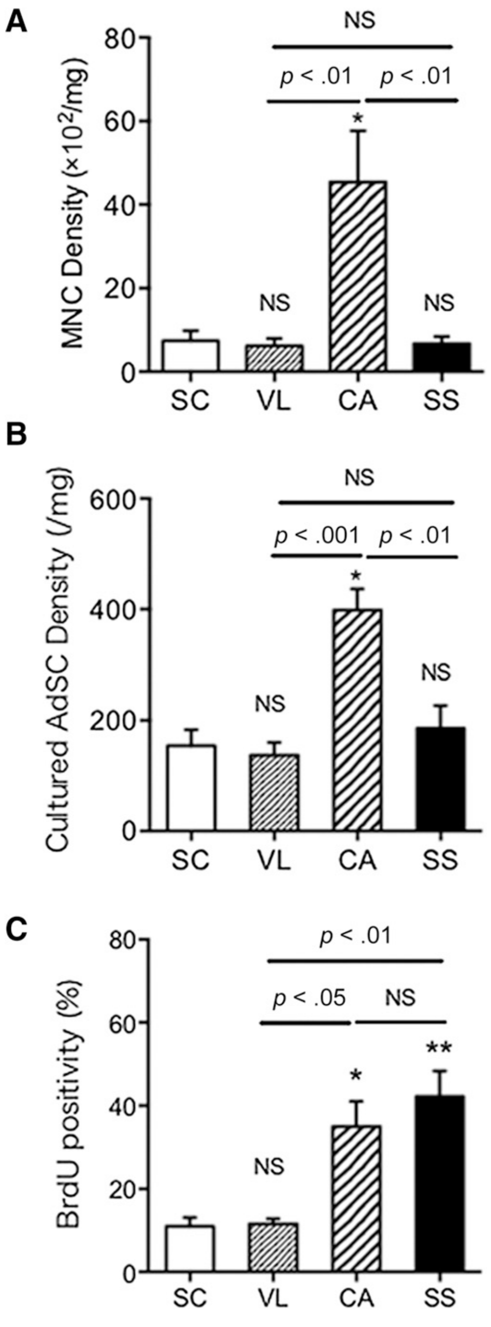

Figure 1. Comparison of cell density, proliferation, and surface markers among AdSCs from four different adipose tissues. **(A, B):** MNCs were isolated from four different adipose tissues (SC, VL, CA, SS), followed by 7 days of culture to obtain adherent AdSCs. The densities in freshly isolated MNCs **(A)** and cultured AdSCs **(B)** from each source were measured and compared among the AdSCs from the four different adipose tissues. **(C):** The positivity of BrdU in AdSCs was assessed by immunocytochemistry. *, $p < .05$; **, $p < .01$; NS, not significant vs. SC. All experiments were performed in triplicate and statistically analyzed. Abbreviations: AdSCs, adipose-derived stem cells; BrdU, 5-bromo-2′-deoxyuridine; CA, cardiac brown adipose tissue; MNCs, mononuclear cells; NS, not significant; SC, subcutaneous white adipose tissue; SS, subscapular brown adipose tissue; VL, visceral white adipose tissue.

isothiocyanate [FITC]/phycoerythrin [PE])-conjugated monoclonal antibodies against CD90 (Abcam), CD44 (eBioscience, San Diego, CA, http://www.ebioscience.com), Sca-1, platelet-derived growth factor receptor-β (PDGFR-β; BioLegend, San Diego,

 ©AlphaMed Press 2016

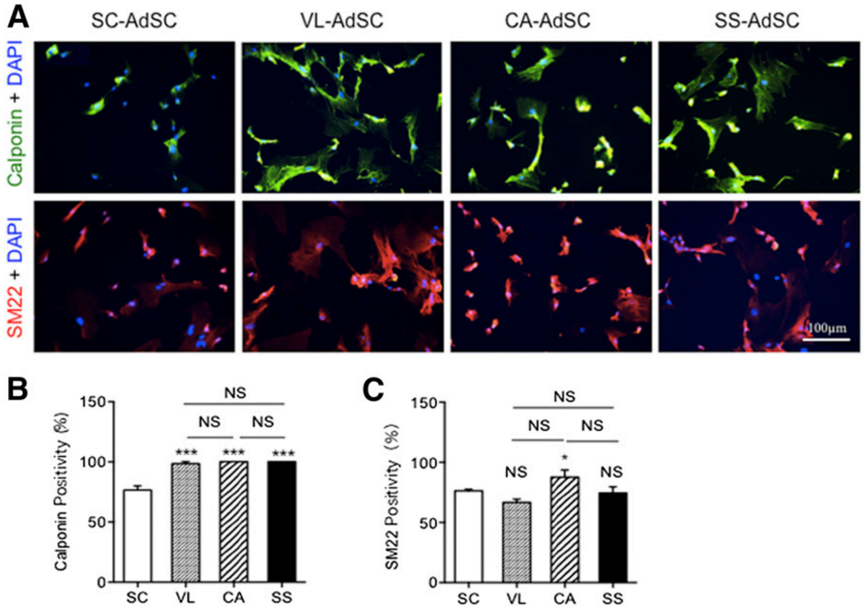

Figure 2. Smooth muscle cell differentiation in AdSCs from four different adipose tissues by fluorescent immunocytochemistry. **(A):** To assess which source for AdSCs could differentiate to smooth muscle cells, the cells were stained with anti-calponin (green) and anti-SM22 (red) antibodies. Nuclei were stained with DAPI (blue). **(B, C):** The rate of calponin-positive **(B)** and SM22-positive **(C)** cells was compared among AdSCs from four different adipose tissues. $*$, $p < .05$; $***$, $p < .001$; NS, not significant vs. SC. All experiments were performed in triplicate and statistically analyzed. Abbreviations: AdSCs, adipose-derived stem cells; CA, cardiac brown adipose tissue; DAPI, 4′,6-diamidino-2-phenylindole; NS, not significant; SC, subcutaneous white adipose tissue; SS, subscapular brown adipose tissue; VL, visceral white adipose tissue.

CA, http://www.biolegend.com), c-kit, CD45, CD34, CD31, and CD105 (BD Pharmingen) (1:100 for each antibody) for 30 minutes on ice. The cells stained with APC/FITC/PE-conjugated rat anti-mouse IgG 2cκ, IgG2a, and IgG2bκ (BioLegend) were used as negative controls. The cells were washed with PBS and fixed with 0.5% PFA/PBS for 15 minutes at RT. The samples were analyzed using a fluorescence-activated cell sorting (FACS) system (Cell Analyzer EC800, Sony, Tokyo, Japan) according to the manufacturer's instructions. The analyses were run in triplicate, and the representative data are presented in the results section. The density of MNCs in each adipose tissue was also calculated by dividing the number of MNCs by the weight of tissues and considered as AdSC density.

Surgical Procedure and AdSC Transfusion Study

Male mice (C57BL6/N, 12–16 weeks old) were anesthetized with an intraperitoneal injection of 400 mg/kg 2,2,2-tribromoethanol (Avertin; Sigma-Aldrich Japan K.K.). MI was induced by ligating the left anterior descending (LAD) coronary artery at a distal site to achieve a 100% survival rate after surgery, and AdSC transfusion was performed as described previously [30]. In brief, the mice were splenectomized to prevent homing of the transfused AdSCs to the spleen. Seven days later, the MI induction procedure was performed, as described previously [31].

For assessment of SC-, VL-, CA-, and SS-derived AdSC recruitment to ischemic myocardium, 10^4 of DiI-labeled freshly isolated MNCs were injected to wild-type (WT) mice via a tail vein 3 days after MI surgery. The mice were sacrificed 7 days after cell injection, and the hearts were harvested for histological analysis. Transfused AdSCs were visualized in red and examined in the sectioned heart samples using fluorescent microscopy.

For assessment of the therapeutic effect of AdSCs on MI and long-term follow-up of recruited AdSCs to the ischemic myocardium, SC- or CA-derived AdSCs (5×10^4) of genetically labeled with *lacZ* transgene isolated from wild-type and B6.129S7-Gt(ROSA)26Sor/J mice (The Jackson Laboratories), respectively, were injected into WT mice via a tail vein 3 days after MI surgery. Cardiac function was sequentially evaluated by echocardiography (Nemio 30; Toshiba Medical Systems, Tochigi, Japan, http://www.toshiba-medical.co.jp) with the following parameters in the left ventricle (LV): changes in ejection fraction (ΔEF), fractional shortening (ΔFS), left ventricular end-systolic dimension (ΔLVD), and left ventricular end-diastolic dimension (ΔLVDd) before, 1 day, and 2, 4, or 6 weeks after MI induction. The mice were sacrificed 6 weeks after MI surgery, and the hearts were harvested for histological analysis.

Morphometric Evaluation of Capillary Density and Infarct Size

The vascularity of the ischemic myocardium was assessed by in situ fluorescent staining using the endothelial cell-specific marker FITC-conjugated BS1-lectin (Vector Laboratories) 6 weeks after MI, as described previously [32]. In brief, after anesthesia, BS1-lectin (0.1 mg per mouse) was injected by direct cardiac puncture systemically. Ten minutes later, the mice were sacrificed, and the hearts were removed and perfused with PBS, followed by 4% PFA/PBS through the right carotid artery retrogradely. The hearts were fixed for 6 hours in 4% PFA/PBS followed by overnight incubation in 20% sucrose/PBS. The tissues were embedded in O.C.T. compound (Sakura FineTek, Tokyo, Japan, http://www.sakura.com) and sectioned at 0.5 mm just below the LAD ligation level at a 5-μm thickness, as described previously [31].

For capillary density measurement, the capillaries were recognized under a fluorescence microscope as tubular structures positive for FITC-BS1-lectin in green. They were evaluated by morphometric examination of three randomly selected fields in the bilateral ischemic

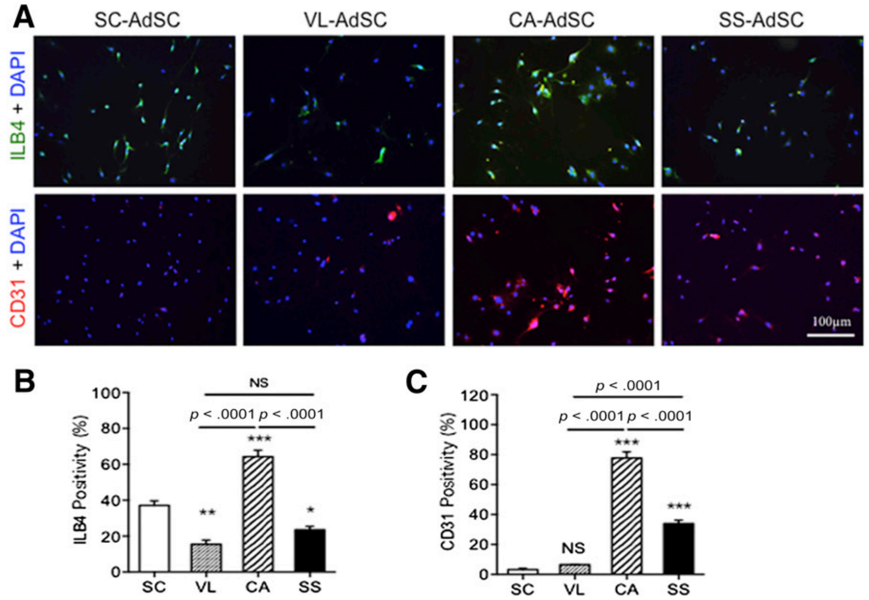

Figure 3. Endothelial differentiation in AdSCs from four different adipose tissues by fluorescent immunocytochemistry. **(A):** To assess which source of AdSCs could differentiate into endothelial cells, the cells were stained with anti-ILB4 (green) and anti-CD31 (red) antibodies. Nuclei were stained with DAPI (blue). **(B, C):** The rate of ILB4-positive **(B)** and CD31-positive **(C)** cells was compared among the AdSCs from four different adipose tissues. $*$, $p < .05$; $**$, $p < .01$; $***$, $p < .001$; NS, not significant vs. SC. All experiments were performed in triplicate and statistically analyzed. Abbreviations: AdSCs, adipose-derived stem cells; CA, cardiac brown adipose tissue; DAPI, 4′,6-diamidino-2-phenylindole; ILB4, isolectin-B4; NS, not significant; SC, subcutaneous white adipose tissue; SS, subscapular brown adipose tissue; VL, visceral white adipose tissue.

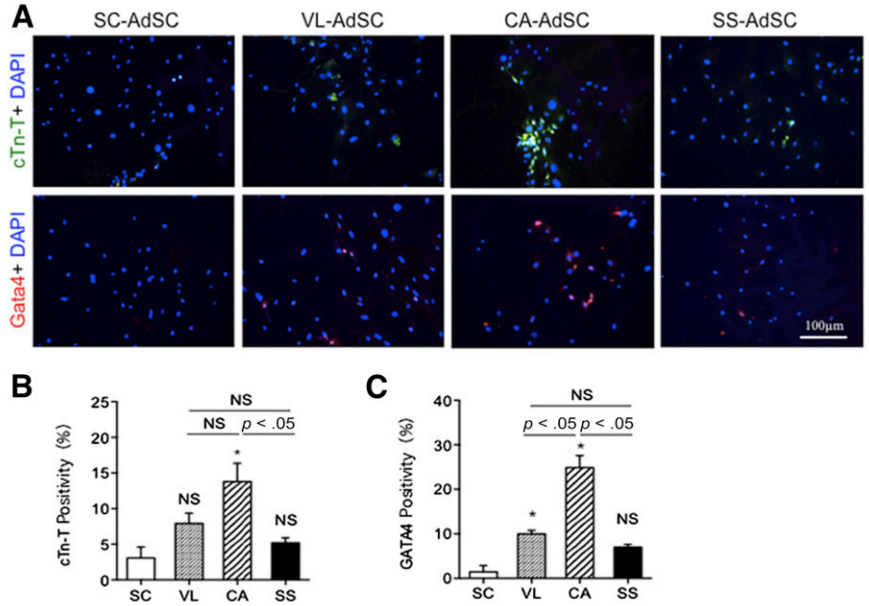

Figure 4. Cardiomyocyte differentiation in AdSCs from four different adipose tissues by fluorescent immunocytochemistry. **(A):** To assess which source for AdSCs could differentiate into cardiomyocytes, the cells were stained with anti-cTnT (green) and anti-GATA4 (red) antibodies. Nuclei were stained with DAPI (blue). **(B, C):** The rate of cTnT-positive **(B)** and GATA4-positive **(C)** cells was compared among AdSCs from four different adipose tissues. $*$, $p < .05$; NS, not significant vs. SC. All experiments were performed in triplicate and statistically analyzed. Abbreviations: AdSCs, adipose-derived stem cells; CA, cardiac brown adipose tissue; cTn-T, cardiac troponin T; DAPI, 4′,6-diamidino-2-phenylindole; NS, not significant; SC, subcutaneous white adipose tissue; SS, subscapular brown adipose tissue; VL, visceral white adipose tissue.

border zone from segments of the LV myocardium survived following LAD occlusion. To assess the severity of myocardial fibrosis/damage, Masson trichrome staining was performed with frozen sections in each tissue block. The stained sections were measured and calculated for the average ratio of fibrosis area (blue) to the entire LV area (percentage of fibrosis area), average ratio of scarred perimeter to the entire LV circumference (percentage of scar length), and the average ratio of the reduced LV wall thickness in the scarred area to the intact LV wall thickness from three different sites in each wall (percentage of LV wall thinning) using NIH ImageJ, version 1.42q, software (NIH, Bethesda, MD, http://www.imagej.nih.gov) and Adobe Photoshop CS4 (Adobe Systems, San Jose, CA, http://www.adobe.com) software.

 ©AlphaMed Press 2016

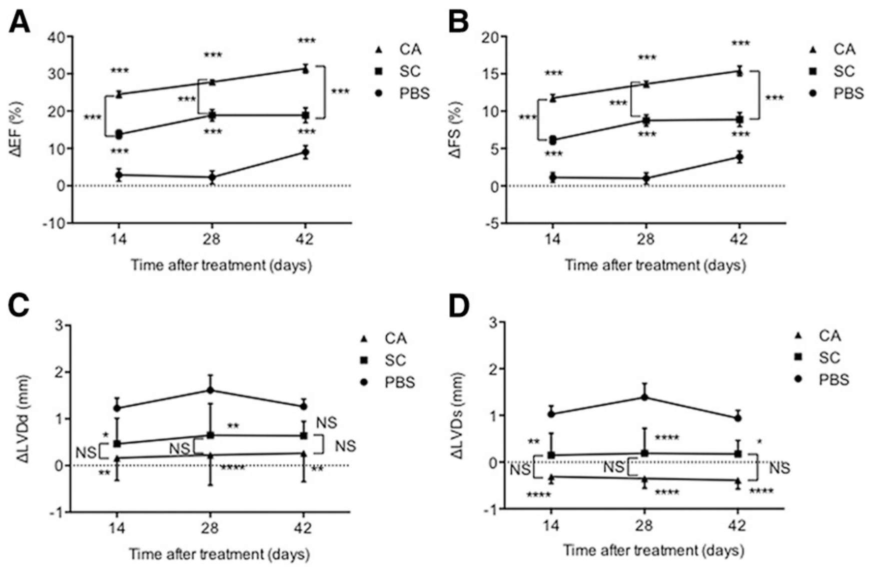

Figure 5. Echocardiographic assessment of cardiac function after myocardial infarction with AdSC transfusion. The cardiac functional parameters in mice were assessed by echocardiography. The EF **(A)**, FS **(B)**, LVDd **(C)**, and LVD **(D)** before and after treatment (14, 28, and 42 days) were measured, and the changes in each parameter (ΔEF, ΔFS, ΔLVDd, and ΔLVD) were statistically analyzed. *, $p < .05$; **, $p < .01$; ***, $p < .001$; ****, $p < .0001$; NS, not significant vs. PBS. Abbreviations: Δ, change; AdSCs, adipose-derived stem cells; CA, cardiac brown adipose tissue; EF, ejection fraction; FS, fractional shortening; LVD, left ventricular end-systolic dimension; LVDd, left ventricular end-diastolic dimension; NS, not significant; PBS, phosphate-buffered saline; SC, subcutaneous white adipose tissue.

Fluorescent Immunohistochemistry

The hearts of the MI-induced mice were harvested at a predetermined time point after surgery and prepared for frozen tissue sectioning after fixation with 4% PFA/PBS. Double fluorescent immunostaining was performed with an antibody against β-galactosidase (β-gal; 1:500; MP Biomedicals, Santa Ana, CA, http://www.mpbio.com) to detect *LacZ* gene expressing exogenously infused AdSCs and FITC-isolectin-B4 (ILB4; 1:100; Vector Laboratories) for the detection of endothelial cells and with an antibody against vascular smooth muscle (SM) α-actin (1:500; Abcam, Tokyo, Japan) for detection of VSMCs or α-sarcomeric actinin (1:200; Abcam, Tokyo, Japan)/cardiac troponin T (1:200; Thermo Fisher Scientific K.K., Yokohama, Japan) for the detection of cardiomyocytes. Normal mouse IgG or rabbit IgG were served as negative controls. Nuclei were counterstained with DAPI (Sigma-Aldrich Japan K.K.), and the sections were mounted in aqueous mounting medium. The images were examined under a fluorescent microscope (BZ8000; Keyence, Osaka, Japan). The number of β-gal/ILB4-positive capillaries, β-gal/SM α-actin-positive VSMCs, and β-gal/α-sarcomeric actinin or cardiac troponin-positive cardiomyocytes were counted in bilateral peri-infarct areas in HPF ($\times$200) and averaged for the assessment of AdSC cardiovascular transdifferentiation frequency in ischemic myocardium.

Statistical Analysis

All values are presented as the mean $\pm$ SEM. Statistical analyses were performed with commercially available software (GraphPad Prism; MDF, Co., Inc., Tokyo, Japan, http://www.mdf-soft.com/english). A comparison between two groups was tested using the Mann-Whitney *U* test, and those among multiple groups were tested for significance via analysis of variance followed by post hoc testing with a Tukey procedure; $p < .05$ was considered statistically significant.

RESULTS

Cardiac Adipose Tissue Contains Increased Numbers of AdSCs With High Proliferation Activity

The MNCs were isolated from four different adipose tissues followed by 7 days of culture to obtain adherent AdSCs. We assessed the cell density by counting the number of MNCs/AdSCs with tissue weights in each adipose tissue (supplemental online Table 4). The cell density of the freshly isolated MNCs was significantly higher in the CA adipose tissue than that in the SC, VL, and SS adipose tissues (Fig. 1A). As expected, the number/density in unit tissue weight of adherent AdSCs derived from CA adipose tissue was also significantly higher than that in SC, VL, and SS adipose tissues (Fig. 1B). We next evaluated the proliferation activity of adherent AdSCs using BrdU incorporation assay. The positivity of BrdU in the CA and SS adipose tissue (supplemental online Fig. 2) was significantly greater than that in the SC and VL adipose tissue. No significant difference was seen between the SC and VL adipose tissue or between the CA and SS adipose tissue (Fig. 1C). These findings suggest that AdSCs are enriched in cardiac adipose tissue exhibiting high proliferation activity.

Immunophenotypic Characterization of AdSCs Isolated From Four Different Adipose Tissues

We assessed the expression of cell surface markers for stem cell (Sca-1 and c-Kit), endothelial (CD31), vascular smooth muscle cell (PDGFR-β), hematopoietic (CD45 and CD34), and mesenchymal (CD90, CD44, and CD105) lineages in freshly isolated MNCs from four different adipose tissues harvested from three mice per each adipose tissue and the 7-day-cultured AdSCs by FACS analysis. Most of all MNCs from all four adipose tissues expressed CD45 but not CD105, CD34, c-Kit, or PDGFR-β, regardless of the adipose tissue origin. A relatively greater number of CD44- and Sca-1-positive cells were observed in VL and SC, respectively. Only CA-derived AdSCs included fewer numbers of CD31-positive cells compared with

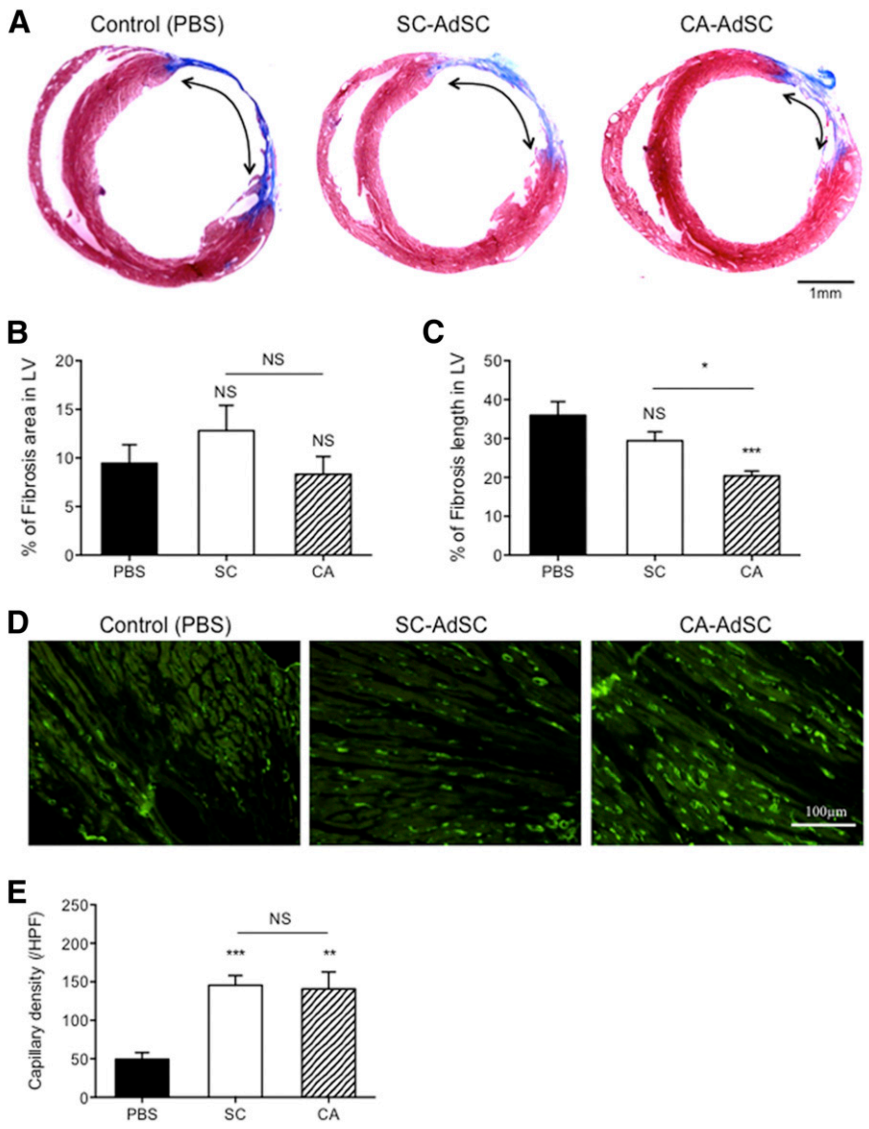

Figure 6. Cardiac morphometric analysis after myocardial infarction with AdSC transfusion. **(A):** Mouse cardiac cross-sections of control group (PBS), SC-AdSC-treated group, and CA-AdSC-treated group through infarcted myocardium 42 days after surgery were assessed by Masson's trichrome staining. The percentage of the mouse LV fibrotic area **(B)** and LV fibrosis length **(C)** were measured. **(D, E):** Mouse cardiac cross-sections were stained with an anti-BS-1 lectin antibody and the endothelial cell marker (green), and the capillary densities were compared among the PBS, SC-AdSC, and CA-AdSC groups. $*$, $p < .05$; $**$, $p < .01$; $***$, $p < .001$; NS, not significant vs. PBS. Abbreviations: AdSCs, adipose-derived stem cells; CA, cardiac brown adipose tissue and CA-AdSC group; HPF, high power field; LV, left ventricle; NS, not significant; PBS, phosphate-buffered saline; SC, subcutaneous white adipose tissue and SC-AdSC group.

the other three adipose tissues, suggesting that vascularity is low in CA compared with the other tissues (supplemental online Table 1; supplemental online Fig. 3). In cultured AdSCs, only VL-derived AdSCs had a low percentage of Sca-1 and a high percentage of CD45, exhibiting characteristics unlike those of MSCs. The other three AdSCs demonstrated a similar expression pattern (high rates of CD44 and Sca-1). However, unlike in human AdSCs, CD90- or CD105-positive cells were few or none, respectively (supplemental online Table 2; supplemental online Fig. 3).

Cardiac AdSCs Exhibit High Differentiation Capacity for Cardiovascular Cells

We assessed the transdifferentiation capacity of AdSCs toward cardiovascular lineage cells using markers of ILB4/CD31 for ECs, markers of calponin/SM22 for VSMCs, and markers of cardiac troponin T (cTnT)/GATA4 for CMs. Although no significant difference was found in the percentages of SM22- and calponin-positive cells (Fig. 2), which was attributed to VSMC differentiation from AdSCs among the four adipose tissues, the cardiac AdSCs exhibited a significant increase in the percentages of ILB4- and CD31-positive cells (Fig. 3) and cTnT- and GATA4-positive cells (Fig. 4), attributed to the endothelial and cardiac differentiation, respectively.

Cardiac AdSC Transplantation Exhibited Therapeutic Efficacy for Myocardial Infarction

First, we evaluated the recruitment potential of freshly isolated DiI-labeled AdSC-rich MNCs (AdSCs, 10^4 per mouse, i.v.) to ischemic myocardium (supplemental online Fig. 4A). The frequencies of SC-, VL-, and CA-derived AdSC recruitment were significantly greater than the frequency of SS-derived AdSC recruitment (supplemental

 ©AlphaMed Press 2016

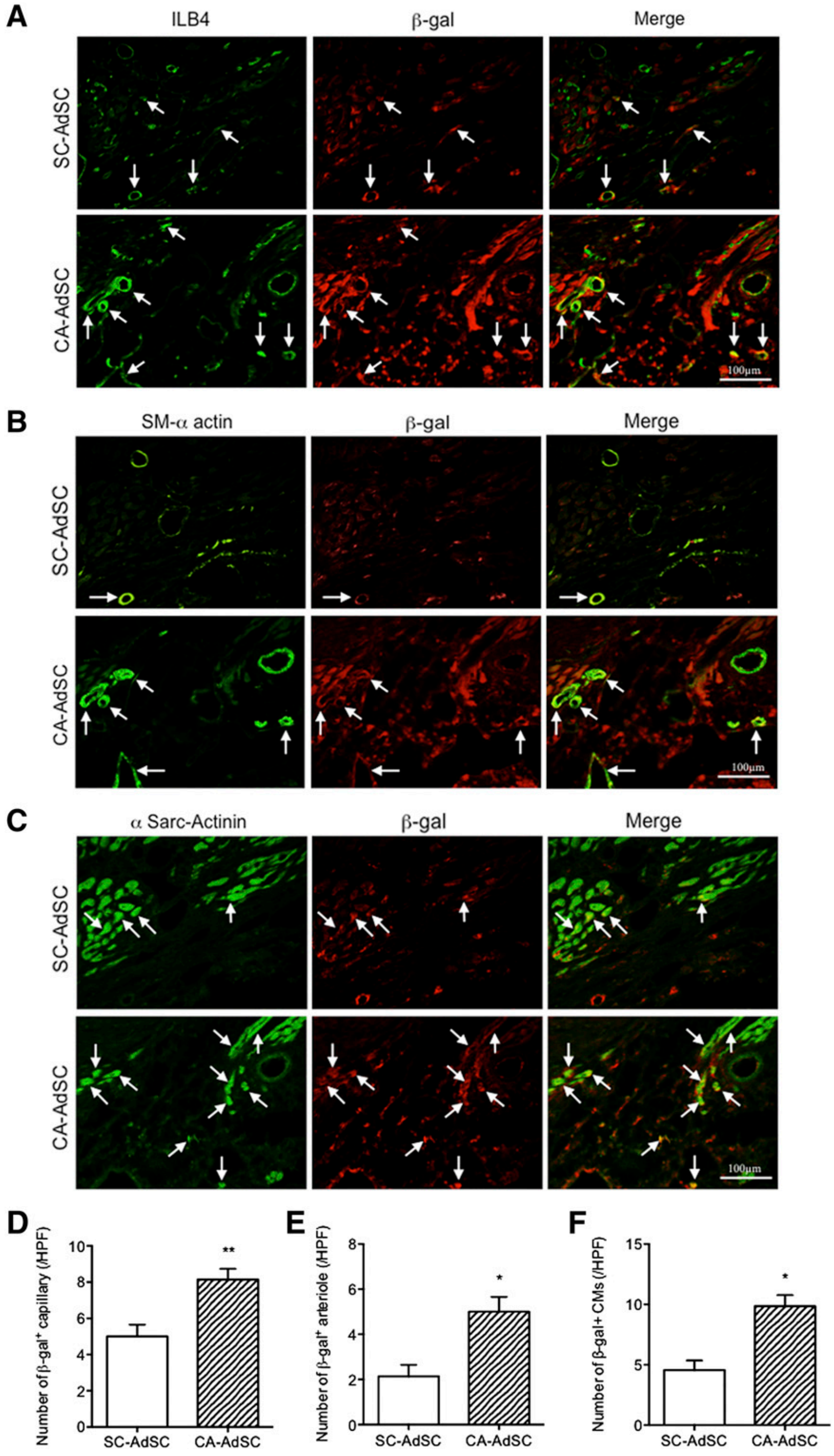

Figure 7. Immunohistological assessment of ischemic myocardium with recruited β-gal expressing AdSCs. Mouse cardiac cross-sections of SC-AdSC-treated group and CA-AdSC-treated group through infarcted myocardium 42 days after surgery were stained with β-gal costained with ILB4, an endothelial cell marker (green) **(A)**, SM-α-actin, a smooth muscle cell marker (green) **(B)**, and a cardiac myocyte marker (green) **(C)**. Arrows indicate the positive cells, where ILB4 **(A)**, SM-α actin **(B)**, and α-Sarc-actinin **(C)** was costained with β-gal. The double-positive cells for β-gal and ILB4, β-gal and SM-α actin, and β-gal and α-Sarc-actinin were quantified and expressed as the number of β-gal$^+$ capillary **(D)**, number of β-gal$^+$ arteriole **(E)**, and number of β-gal$^+$ CMs **(F)**, respectively. $*$, $p < .05$; $**$, $p < .01$ vs. SC-AdSCs. Abbreviations: α-Sarc-Actinin, α-sarcomeric actinin; AdSCs, adipose-derived stem cells; β-gal, β-galactosidase; CA, cardiac brown adipose tissue; HPF, high power field; ILB4, isolectin-B4; SC, subcutaneous white adipose tissue; SM-α actin, smooth muscle α-actin.

online Fig. 4B). Double immunofluorescent staining demonstrated that both recruited SC- and CA-AdSCs differentiated into ILB4-/SM-α-actin-positive vascular lineage cells (supplemental online Fig. 4C, 4D), and only CA-AdSCs could differentiate into cTnT-positive cardiomyocytes by day 7 after cell transfusion (supplemental online Fig. 4E). The quantitative analysis for the cardiovascular differentiation (supplemental online Fig. 4F) also allowed us to focus on CA- and SC-AdSCs, excluding VL- and SS-AdSCs in the next series of experiments. Next, we compared the therapeutic efficacy of CA-AdSCs with that of SC-AdSCs, both are easy to obtain compared with VL- and SS-AdSCs and commonly used for autologous AdSC transplantation in clinical settings. Consistent with the results in previous studies, SC-AdSC transplantation significantly improved cardiac functions, which was assessed by the changes in the echocardiographic parameters, ΔEF, ΔFS, ΔLVDd, and ΔLVDs. Notably, further significant cardiac functional recovery by CA-AdSC transplantation was observed compared with that by SC-AdSC transplantation (Fig. 5). The histological analysis also revealed significant reductions in the infarct area, assessed by the fibrosis length in the cross-sectional LV area treated with CA-AdSC transplantation compared with PBS (control) and SC-AdSC transplantation (Fig. 6A–6C). The immunofluorescent staining (Fig. 6D) exhibited a significant increase in capillary density in the ischemic border zone of the LV in both the SC- and CA-AdSC groups compared with the control group; however, no significant difference was found in the SC- and CA-AdSC groups (Fig. 6E).

Recruited Cardiac AdSCs Differentiated Into Cardiovascular Cells in Ischemic Myocardium

Finally, we examined the infarct heart samples transplanted with SC- versus CA-AdSCs isolated from mice constitutively expressing the *lacZ* gene in whole cells to assess whether the transplanted AdSCs contributed to cardiac tissue regeneration. The cardiovascular cell differentiation of the transplanted AdSCs in the ischemic myocardium was evaluated using double immunofluorescent staining for β-gal, a transcript of the *lacZ* gene, and ILB4 for ECs (Fig. 7A), SM-α-actin for VSMCs (Fig. 7B), and α-sarcomeric actinin for CM (Fig. 7C). The numbers of the ILB4/β-gal, SM-α-actin/β-gal, and α-sarcomeric actinin/β-gal double-positive cells were significantly greater in the CA-AdSC group than in the SC-AdSC group (Fig. 7D–7F), suggesting that the recruited CA-AdSCs frequently differentiate into cardiovascular cells in ischemic myocardium compared with the recruited SC-AdSCs. Because we did not perform sex-mismatched fluorescent in situ hybridization analysis, we could not distinguish cell fusion and transdifferentiation of the recruited AdSCs scientifically; however, a recent report did not find that the recruited human adipose-derived MNCs and AdSCs fused with host mouse cells [26].

Discussion

In the present study, we have demonstrated that both MNCs and AdSCs isolated from cardiac adipose tissue (CA-AdSCs) have a prominent potential for differentiation into cardiovascular cells, in particular, ECs and CMs, compared with those isolated from adipose tissue in other organs. ECs are a major component of the capillaries for angiogenesis, and CMs are essential in the myocardium for cardiac functionality. These cellular differentiation potentials of AdSCs are therefore important for cardiac tissue regeneration. Our in vivo data have indicated the superior therapeutic efficacy of CA-AdSCs in myocardial infarction, contributing to cardiovascular regeneration by transdifferentiation, but, probably, not by cell fusion [26], into ECs, VSMCs, and CMs in ischemic myocardium compared with SC-AdSCs. In recent ongoing clinical studies, freshly isolated MNCs (SVF) from subcutaneous adipose tissue have been frequently used as adipose-derived regenerative cells, exhibiting favorable outcomes in ischemic cardiovascular diseases. Nevertheless, both freshly isolated MNCs (SVF) and cultured AdSCs have been shown to have a similar therapeutic potential in acute myocardial infarction [26].

In humans, mesenchymal stem cells originate from a variety of organs (i.e., bone marrow, adipose tissue, placenta, amnion, amniotic fluid, and cord blood). AdSCs have been characterized with positive cell surface markers of CD90, CD44, CD29, and CD105 and negative cell surface markers of CD45 and CD31 [33]. Regarding CD34 expression in human AdSCs, it has been reported to vary, depending on the isolation or culture method [34]. In addition to the marker expression pattern in human AdSCs, Sca-1 has been described as an additional marker in mouse AdSCs [35, 36]. Consistent with previous reports, our data have indicated an increased percentage of Sca-1-positive cells in mouse AdSCs after culturing. However, we detected only a reduced percentage of CD90-positive ($<$10%) and a few CD105-positive ($<$1%) cells even in culture conditions (supplemental online Tables 1, 2). The discrepancy between the previous reports and our data might have been because of the following reasons: (a) CD90 expression generally increases after successive passages [35] and is high when AdSCs are isolated from lymph node-containing adipose tissue [36]; (b) CD105 expression can be affected by trypsin, depending on the type and/or activity [37] in trypsinized AdSCs for FACS analysis; and (c) the variability of antibody specificity/function to recognize cell surface antigen in each report.

Regardless of the variation in cell surface antigen expression to identify or characterize AdSCs, SC-AdSCs, specifically, have been shown to have a therapeutic effect on cardiac functional recovery after myocardial infarction in experimental animal models. Previous studies have demonstrated a favorable effect of SC-AdSCs, along with an indirect paracrine mechanism [19, 38, 39] by which angiogenesis/neovascularization including bone marrow-derived endothelial progenitor cell recruitment to ischemic myocardium [19] is promoted, on improved cardiac functional recovery, rather than a direct contribution by the transplanted SC-AdSCs to tissue regeneration. In contrast, our study has demonstrated a therapeutic effect of CA-AdSCs on MI via a direct contribution to the ischemic myocardium and transdifferentiation to, at least in part, CMs, ECs, and VSMCs, in addition to the paracrine effect (Fig. 7). Although a very recent similar study [40] also demonstrated the superior therapeutic effect of pericardiac AdSCs on cardiac functional recovery and tissue regeneration compared with that of subcutaneous AdSCs in a rat MI model, only a few transplanted or intramuscularly injected pericardiac AdSCs (5×10^5 at the center of infarcted wall) were engrafted in the ischemic myocardium 28 days after surgery. This suggests that the transplanted cardiac AdSCs could not survive in acute ischemic and inflammatory conditions without differentiation into cardiovascular cells. In order to overcome the problems in the previous study, we systemically injected CA-AdSCs via a tail vein in mice 3 days after MI induction. In this setting, the systemically infused cells were recruited to the myocardial ischemic border zone, where ischemia and inflammation is not severe. These AdSCs could survive even 28 days after surgery.

Most of the previous mouse studies applied SC-AdSCs at doses of 5×10^5 to 5×10^6 in the coronary artery ligation-induced

 ©AlphaMed Press 2016

MI model in a cardiac intramuscular fashion (supplemental online Table 3). In contrast, our approach of only 10^4 of CA-AdSCs with intravenous infusion significantly improved cardiac functional recovery after MI, which might be attributed to (a) prevention of the injected AdSCs homing to the spleen and (b) altered postinfarction myocardial inflammation by the splenectomy. This is a study limitation and a potential bias of our study. Another distinct point of our study was the source of AdSCs from supracardiac brown adipose tissue (supplemental online Fig. 1) but not from pericardial white adipose tissue [41]. Although the differences and similarity of white and brown adipose tissue-derived stem cells has been investigated [42] (supplemental online Table 5), not only the difference in adipose tissue type (white vs. brown) but also the origin of fat tissue (i.e., cardiac origin) might be a critical factor in determining the therapeutic effect on MI and cell fate in ischemic myocardium.

Conclusion

CA-AdSCs tend to differentiate into cardiovascular lineage cells (i.e., cardiomyocytes, endothelial cells, and vascular smooth muscle cells) compared with other (SC, VL, and SS) adipose tissue-derived stem cells. A small number of systemically transplanted CA-AdSCs sufficiently improved cardiac functional recovery after MI by differentiating into cardiovascular cells in ischemic myocardium. Because supracardiac brown adipose tissue also exists below the thymus in a region free of major vessels in human [43], the strategy for cardiac regeneration with CA-AdSCs might give rise to a new autologous stem cell therapy for patients with myocardial ischemia. Cardiac adipose tissue can be easily harvested during open chest coronary bypass graft surgery or other cardiovascular procedures, and patients will be able to use their own AdSCs with the cell bank system after cell culture expansion to treat future recurrent ischemic heart disease.

Author Contributions

H.N.: collection and/or assembly of data, manuscript writing; M.I.: conception and design, collection and/or assembly of data, data analysis and interpretation, manuscript writing; E.K.: collection and/or assembly of data; M.H. and T.H.: financial support; M.A.: manuscript writing, final approval of manuscript.

Disclosure of Potential Conflicts of Interest

The authors indicated no potential conflicts of interest.

References

1 Guilbert JJ. The world health report 2002—Reducing risks, promoting healthy life. Educ Health (Abingdon) 2003;16:230.

2 Oguz E, Ayik F, Ozturk P et al. Long-term results of autologous stem cell transplantation in the treatment of patients with congestive heart failure. Transplant Proc 2011;43:931–934.

3 Losordo DW, Schatz RA, White CJ et al. Intramyocardial transplantation of autologous CD34+ stem cells for intractable angina: A phase I/IIa double-blind, randomized controlled trial. Circulation 2007;115:3165–3172.

4 Leistner DM, Fischer-Rasokat U, Honold J et al. Transplantation of progenitor cells and regeneration enhancement in acute myocardial infarction (TOPCARE-AMI): Final 5-year results suggest long-term safety and efficacy. Clin Res Cardiol 2011;100:925–934.

5 Wollert KC, Meyer GP, Lotz J et al. Intracoronary autologous bone-marrow cell transfer after myocardial infarction: The BOOST randomised controlled clinical trial. Lancet 2004;364:141–148.

6 Amado LC, Saliaris AP, Schuleri KH et al. Cardiac repair with intramyocardial injection of allogeneic mesenchymal stem cells after myocardial infarction. Proc Natl Acad Sci USA 2005;102:11474–11479.

7 Döbert N, Britten M, Assmus B et al. Transplantation of progenitor cells after reperfused acute myocardial infarction: Evaluation of perfusion and myocardial viability with FDG-PET and thallium SPECT. Eur J Nucl Med Mol Imaging 2004;31:1146–1151.

8 Rodriguez AM, Pisani D, Dechesne CA et al. Transplantation of a multipotent cell population from human adipose tissue induces dystrophin expression in the immunocompetent mdx mouse. J Exp Med 2005;201:1397–1405.

9 Shoji T, Ii M, Mifune Y et al. Local transplantation of human multipotent adipose-derived stem cells accelerates fracture healing via enhanced osteogenesis and angiogenesis. Lab Invest 2010;90:637–649.

10 Huang JI, Beanes SR, Zhu M et al. Rat extramedullary adipose tissue as a source of osteochondrogenic progenitor cells. Plast Reconstr Surg 2002;109:1033–1042.

11 Lin Y, Luo E, Chen X et al. Molecular and cellular characterization during chondrogenic differentiation of adipose tissue-derived stromal cells in vitro and cartilage formation in vivo. J Cell Mol Med 2005;9:929–939.

12 Timper K, Seboek D, Eberhardt M et al. Human adipose tissue-derived mesenchymal stem cells differentiate into insulin, somatostatin, and glucagon expressing cells. Biochem Biophys Res Commun 2006;341:1135–1140.

13 Aurich H, Sgodda M, Kaltwasser P et al. Hepatocyte differentiation of mesenchymal stem cells from human adipose tissue in vitro promotes hepatic integration in vivo. Gut 2009;58:570–581.

14 Jang S, Cho HH, Cho YB et al. Functional neural differentiation of human adipose tissue-derived stem cells using bFGF and forskolin. BMC Cell Biol 2010;11:25.

15 Liqing Y, Jia G, Jiqing C et al. Directed differentiation of motor neuron cell-like cells from human adipose-derived stem cells in vitro. Neuroreport 2011;22:370–373.

16 Planat-Bénard V, Menard C, André M et al. Spontaneous cardiomyocyte differentiation from adipose tissue stroma cells. Circ Res 2004;94:223–229.

17 Fischer LJ, McIlhenny S, Tulenko T et al. Endothelial differentiation of adipose-derived stem cells: Effects of endothelial cell growth supplement and shear force. J Surg Res 2009;152:157–166.

18 Harris LJ, Abdollahi H, Zhang P et al. Differentiation of adult stem cells into smooth muscle for vascular tissue engineering. J Surg Res 2011;168:306–314.

19 Ii M, Horii M, Yokoyama A et al. Synergistic effect of adipose-derived stem cell therapy and bone marrow progenitor recruitment in ischemic heart. Lab Invest 2011;91:539–552.

20 Gonzalez-Rey E, Anderson P, González MA et al. Human adult stem cells derived from adipose tissue protect against experimental colitis and sepsis. Gut 2009;58:929–939.

21 Gonzalez-Rey E, Gonzalez MA, Varela N et al. Human adipose-derived mesenchymal stem cells reduce inflammatory and T cell responses and induce regulatory T cells in vitro in rheumatoid arthritis. Ann Rheum Dis 2010;69:241–248.

22 Perin EC, Sanz-Ruiz R, Sanchez PL et al. Adipose-derived regenerative cells in patients with ischemic cardiomyopathy: The PRECISE trial. Am Heart J 2014;168:88.e92–95.e82.

23 Perez-Cano R, Vranckx JJ, Lasso JM et al. Prospective trial of adipose-derived regenerative cell (ADRC)-enriched fat grafting for partial mastectomy defects: The RESTORE-2 trial. Eur J Surg Oncol 2012;38:382–389.

24 Kølle SF, Fischer-Nielsen A, Mathiasen AB et al. Enrichment of autologous fat grafts with ex-vivo expanded adipose tissue-derived stem cells for graft survival: A randomised placebo-controlled trial. Lancet 2013;382:1113–1120.

25 Kamakura T, Ito K. Autologous cell-enriched fat grafting for breast augmentation. Aesthetic Plast Surg 2011;35:1022–1030.

26 Bai X, Yan Y, Song YH et al. Both cultured and freshly isolated adipose tissue-derived stem cells enhance cardiac function after acute myocardial infarction. Eur Heart J 2010;31:489–501.

27 Jeon ES, Moon HJ, Lee MJ et al. Sphingosylphosphorylcholine induces differentiation of human mesenchymal stem cells into smooth-muscle-like cells through a TGF-beta-dependent mechanism. J Cell Sci 2006;119:4994–5005.

28 Konno M, Hamazaki TS, Fukuda S et al. Efficiently differentiating vascular endothelial cells from adipose tissue-derived mesenchymal stem cells in serum-free culture. Biochem Biophys Res Commun 2010;400:461–465.

29 Park E, Patel AN. PKC-delta induces cardiomyogenic gene expression in human adipose-derived stem cells. Biochem Biophys Res Commun 2010;393:582–586.

30 Ii M, Takenaka H, Asai J et al. Endothelial progenitor thrombospondin-1 mediates diabetes-induced delay in reendothelialization following arterial injury. Circ Res 2006;98:697–704.

31 Ii M, Nishimura H, Iwakura A et al. Endothelial progenitor cells are rapidly recruited to myocardium and mediate protective effect of ischemic preconditioning via "imported" nitric oxide synthase activity. Circulation 2005;111:1114–1120.

32 Ii M, Nishimura H, Kusano KF et al. Neuronal nitric oxide synthase mediates statin-induced restoration of vasa nervorum and reversal of diabetic neuropathy. Circulation 2005;112:93–102.

33 Zuk PA, Zhu M, Ashjian P et al. Human adipose tissue is a source of multipotent stem cells. Mol Biol Cell 2002;13:4279–4295.

34 Yoshimura K, Shigeura T, Matsumoto D et al. Characterization of freshly isolated and cultured cells derived from the fatty and fluid portions of liposuction aspirates. J Cell Physiol 2006;208:64–76.

35 Taha MF, Hedayati V. Isolation, identification and multipotential differentiation of mouse adipose tissue-derived stem cells. Tissue Cell 2010;42:211–216.

36 Yamamoto N, Akamatsu H, Hasegawa S et al. Isolation of multipotent stem cells from mouse adipose tissue. J Dermatol Sci 2007;48:43–52.

37 Tabatabaei M, Mosaffa N, Nikoo S et al. Isolation and partial characterization of human amniotic epithelial cells: The effect of trypsin. Avicenna J Med Biotechnol 2014;6: 10–20.

38 Rehman J, Traktuev D, Li J et al. Secretion of angiogenic and antiapoptotic factors by human adipose stromal cells. Circulation 2004;109:1292–1298.

39 Salgado AJ, Reis RL, Sousa NJ et al. Adipose tissue derived stem cells secretome: Soluble factors and their roles in regenerative medicine. Curr Stem Cell Res Ther 2010;5: 103–110.

40 Wang X, Zhang H, Nie L et al. Myogenic differentiation and reparative activity of stromal cells derived from pericardial adipose in comparison to subcutaneous origin. Stem Cell Res Ther 2014;5:92.

41 Hamdi H, Planat-Benard V, Bel A et al. Epicardial adipose stem cell sheets results in greater post-infarction survival than intramyocardial injections. Cardiovasc Res 2011; 91:483–491.

42 Silva FJ, Holt DJ, Vargas V et al. Metabolically active human brown adipose tissue derived stem cells. STEM CELLS 2014;32: 572–581.

43 Cheung L, Gertow J, Werngren O et al. Human mediastinal adipose tissue displays certain characteristics of brown fat. Nutr Diabetes 2013;3:e66.

See www.StemCellsTM.com for supporting information available online.

 ©AlphaMed Press 2016

CXC-Chemokine Receptor 4 Antagonist AMD3100 Promotes Cardiac Functional Recovery After Ischemia/Reperfusion Injury via Endothelial Nitric Oxide Synthase–Dependent Mechanism

Kentaro Jujo, MD, PhD; Masaaki Ii, MD, PhD; Haruki Sekiguchi, MD, PhD; Ekaterina Klyachko, BS; Sol Misener, BS; Toshikazu Tanaka, MD; Jörn Tongers, MD, PhD; Jérôme Roncalli, MD, PhD; Marie-Ange Renault, PhD; Tina Thorne, BS; Aiko Ito, BS; Trevor Clarke, BS; Christine Kamide, BS; Yukio Tsurumi, MD, PhD; Nobuhisa Hagiwara, MD, PhD; Gangjian Qin, MD, PhD; Michio Asahi, MD, PhD; Douglas W. Losordo, MD

Background**—**CXC-chemokine receptor 4 (CXCR4) regulates the retention of stem/progenitor cells in the bone marrow (BM), and the CXCR4 antagonist AMD3100 improves recovery from coronary ligation injury by mobilizing stem/progenitor cells from the BM to the peripheral blood. Thus, we investigated whether AMD3100 also improves recovery from ischemia/reperfusion injury, which more closely mimics myocardial infarction in patients, because blood flow is only temporarily obstructed.

Methods and Results**—**Mice were treated with single subcutaneous injections of AMD3100 (5 mg/kg) or saline after ischemia/reperfusion injury. Three days later, histological measurements of the ratio of infarct area to area at risk were smaller in AMD3100-treated mice than in mice administered saline, and echocardiographic measurements of left ventricular function were greater in the AMD3100-treated mice at week 4. $CXCR4^+$ cells were mobilized for just 1 day in both groups, but the mobilization of $sca1^+/flk1^+$ cells endured for 7 days in AMD3100-treated mice compared with just 1 day in the saline-treated mice. AMD3100 upregulated BM levels of endothelial nitric oxide synthase (eNOS) and 2 targets of eNOS signaling, matrix metalloproteinase-9 and soluble Kit ligand. Furthermore, the loss of BM eNOS expression abolished the benefit of AMD3100 on $sca1^+/flk1^+$ cell mobilization without altering the mobilization of $CXCR4^+$ cells, and the cardioprotective effects of AMD3100 were retained in eNOS-knockout mice that had been transplanted with BM from wild-type mice but not in wild-type mice with eNOS-knockout BM.

Conclusions**—**AMD3100 prolongs BM progenitor mobilization and improves recovery from ischemia/reperfusion injury, and these benefits appear to occur through a previously unidentified link between AMD3100 and BM eNOS expression. **(*Circulation.* 2013;127:63-73.)**

Key Words: ischemia ■ myocardium ■ nitric oxide synthase ■ pharmaceutical preparations ■ reperfusion

Downloaded from http://ahajournals.org by on April 30, 2021

In response to ischemic myocardial injury, stem/progenitor cells are mobilized from the bone marrow (BM) to the peripheral blood (PB) and become incorporated into the injured tissue, where a subset of the mobilized cells, including endothelial progenitor cells (EPCs), contribute to cardiac recovery by enhancing vessel growth.[1–3] Before mobilization, progenitor cells are sequestered in the BM by interactions between CXC chemokine receptor 4 (CXCR4) and stromal-cell–derived factor 1 (SDF-1).[4,5] Mobilization is triggered when this interaction is disrupted, and SDF-1 expression in the ischemic tissue contributes to the recruitment and incorporation of mobilized EPCs.[6] SDF-1 also induces the migration of EPCs in vitro,[7] and SDF-1–CXCR4 signaling appears to influence EPC proliferation and survival.[8,9] Thus, the SDF-1/CXCR4 axis is a key regulator of the activity of stem/progenitor cells, including EPCs, particularly the release from BM and the retention/recruitment of progenitors in/to ischemic tissue.

Editorial see p 13
Clinical Perspective on p 73

CXCR4 also facilitates cellular entry of the human immunodeficiency virus, which prompted the development of

Received February 10, 2012; accepted October 26, 2012

From the Feinberg Cardiovascular Research Institute, Feinberg School of Medicine, Northwestern University, Chicago, IL (K.J., E.K., S.M., T.T., J.T., J.R., M.-A.R., T.T., A.I., T.C., C.K., G.Q., D.W.L.); Department of Cardiology, Tokyo Women's Medical University, Tokyo, Japan (K.J., H.S., Y.T., N.H.); Department of Pharmacology, Faculty of Medicine, Osaka Medical College, Osaka, Japan (M.I., M.A.); and Department of Cardiology and Angiology, Hannover Medical School, Hannover, Germany (J.T.).

The online-only Data Supplement is available with this article at http://circ.ahajournals.org/lookup/suppl/doi:10.1161/CIRCULATIONAHA.112.099242/-/DC1.

Correspondence: Masaaki Ii, MD, PhD, Department of Pharmacology, Faculty of Medicine, Osaka Medical College, 2–7, Daigaku-machi, Takatsuki, Osaka, 569–8686, Japan. E-mail masa0331@mac.com

© 2012 American Heart Association, Inc.

Circulation **is available at http://circ.ahajournals.org** **DOI: 10.1161/CIRCULATIONAHA.112.099242**

AMD3100, a pharmacological CXCR4 antagonist.[10–12] In early pharmacokinetic studies, a single intravenous dose of AMD3100 unexpectedly increased circulating white blood cell counts in healthy volunteers,[13] and subsequent reports indicate that AMD3100 rapidly mobilizes hematopoietic progenitor cells in both humans and mice by reversibly blocking the SDF-1–CXCR4 interaction.[13–16] Previously, we have shown that a single dose of AMD3100 after surgical ligation of the coronary artery[17] increases the mobilization of BM progenitor cells (BMPCs), which leads to a greater BMPC accumulation in infarcted tissue and to improvements in vascularity and myocardial performance; furthermore, the effect of AMD3100 on BMPC mobilization endured for >1 week. This is somewhat surprising because the half-life of AMD3100 in serum is just 2 to 3 hours and consequently the short-term activity of AMD3100 as a CXCR4 antagonist should dissipate within a day of administration. Here, we investigated whether AMD3100 also improves myocardial recovery after ischemia/reperfusion (IR) injury, which more closely resembles the clinical presentation of acute myocardial infarction, because blood flow is obstructed temporarily rather than permanently. We also compared the time course and signaling pathways involved in BMPC mobilization and those associated with mobilization of CXCR4$^+$ mononuclear cells (MNCs).

Methods

Injury Model and Treatment

All mice were obtained from The Jackson Laboratories. BM transplantation surgery and IR injury were performed as described previously[18–20] and as summarized in the online-only Data Supplement. Mice received a single subcutaneous injection of AMD3100 (5 mg/kg, 125 μg in 100 μL; Sigma-Aldrich) or an equal volume of saline immediately after surgery was complete. Area at risk (AAR) and infarct area were measured as described in the online-only Data Supplement. AAR was presented as a percentage of the area of the entire left ventricle (LV); the infarct area, as a percentage of the AAR.

Physiological Assessments of LV Function

Echocardiographic measurements were performed with a commercially available high-resolution echocardiographic system (VEVO 770, VisualSonics Inc). End-systolic and end-diastolic LV areas on the short-axis view were traced at the midpapillary muscle level according to the instruction of the echocardiographic program, and the following calculation was used for area fractional shortening (FS%): (diastolic LV area−systolic LV area)/diastolic LV area.

PB Cell Counts

Detailed information is provided in the online-only Data Supplement.

Histological and Immunofluorescent Assessments

Detailed information is provided in the online-only Data Supplement.

In Vitro Assessments With Cultured, BM-Derived EPCs

Detailed information is provided in the online-only Data Supplement.

Luciferase Reporter Assay

Detailed information is provided in the online-only Data Supplement.

Quantitative Real-Time Reverse Transcription–Polymerase Chain Reaction

Detailed information, including primer and probe sequences (Table I), is provided in the online-only Data Supplement.

Statistical Analysis

All values were expressed as mean±SEM. Comparisons among samples from different mice at single or multiple time points were evaluated by unpaired *t* test (bar graph). Comparisons among consecutive samples from identified mouse in a single group were evaluated by 1-way ANOVA with the Bonferroni post hoc test (line graph). Comparisons among consecutive samples from identified mouse in multiple groups were evaluated by 2-way ANOVA with the Bonferroni post hoc test (line graph; the factors are the groups and time). We applied the Bonferroni adjustment to both comparisons of the groups within each time point and comparisons of each time point and baseline within each group only when the data were collected from identical mouse at multiple time points. A 2-sided value of $P<0.05$ was considered statistically significant.

Results

The in vivo experiments for histological and echocardiographic analyses were performed separately. Therefore, the difference in sample sizes for each group is due to different series of experiments.

AMD3100 Treatment Reduces Infarct Size and Improves Cardiac Performance After IR Injury

IR injury was induced by surgically occluding the left anterior descending artery for 60 minutes, and AMD3100 (125 μg in 100 μL) or an equal volume of saline was injected subcutaneously immediately after surgery was complete. Infarct size and the AAR for infarction were evaluated 3 days after IR injury by briefly reoccluding the left anterior descending artery, perfusing the hearts with microspheres, and then staining sections of heart tissue with triphenyltetrazolium chloride (Figure 1A). Viable tissue was stained deep red; the infarcted region remained colorless; and the AAR was identified by the absence of microspheres. The size of the AAR was similar in both treatment groups (Figure 1B), but the infarcted regions were significantly smaller in AMD3100-treated mice than in mice administered saline (Figure 1C). AMD3100 treatment was also associated with significantly less apoptosis on day 3 (Figure 1D) and with significantly less fibrosis on day 28 (Figure 1E) after IR injury.

Cardiac function was measured before IR injury and 7, 14, and 28 days afterward via echocardiographic assessments of LV fractional shortening, LV systolic area, and LV diastolic area. Fractional shortening was significantly greater and LV systolic area was significantly smaller in mice administered AMD3100 than in saline-treated mice on days 14 and 28 after IR injury, whereas LV diastolic area did not differ significantly between groups at any time point (Figure 1F–1H and Tables II and III in the online-only Data Supplement). Collectively, the results from these

Downloaded from http://ahajournals.org by on April 30, 2021

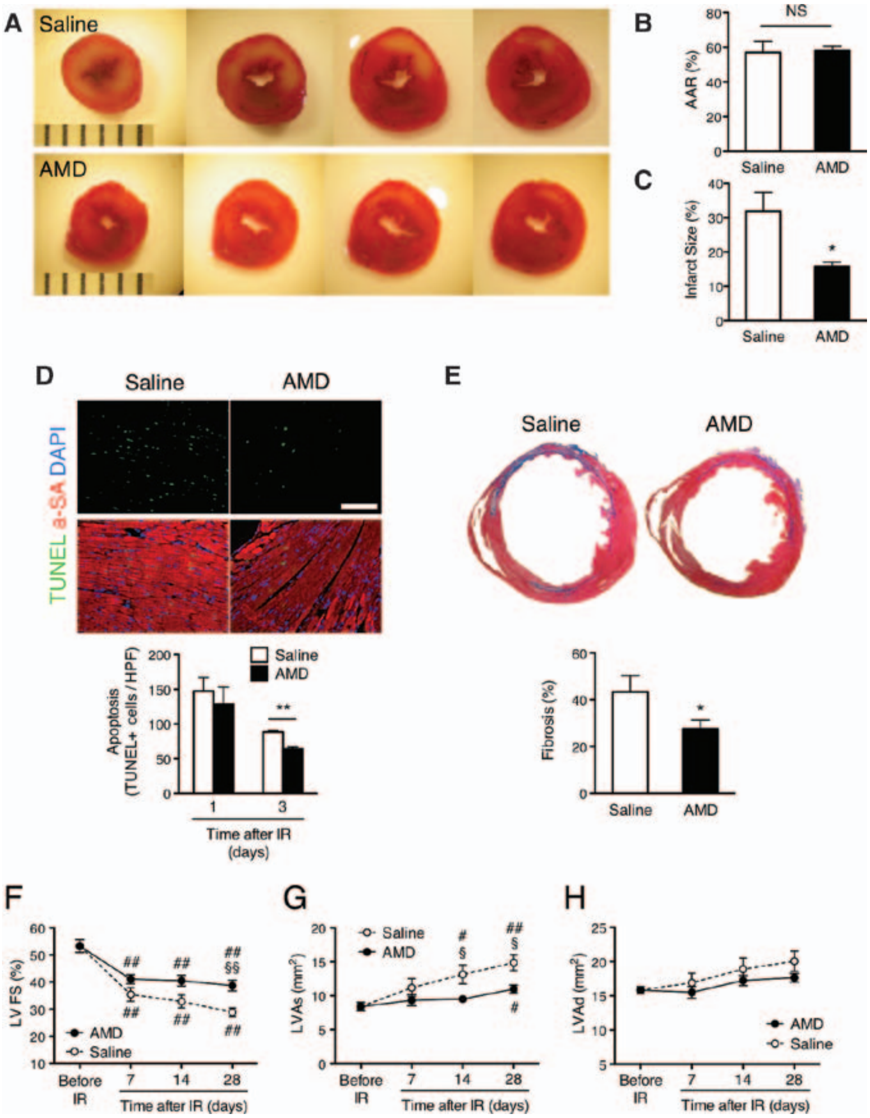

Figure 1. AMD3100 treatment improves cardiac function and reduces infarct size after ischemia/reperfusion (IR) injury. Mice were treated with saline alone or with 100 µL saline containing 125 µg AMD3100 after surgically induced IR injury. **A** through **C**, Area at risk (AAR) and infarct size were evaluated 3 days after IR injury via in vivo microsphere perfusion and triphenyltetrazolium chloride staining. **A**, Viable tissue stained deep red, and the infarcted region is colorless. Scale=1-mm increments. **B**, The AAR was identified by the absence of microspheres and is presented as a percentage of the total left ventricular (LV) area. **C**, Infarct size was normalized to the size of the AAR and presented as a percentage. **D**, Apoptosis was evaluated in terminal deoxynucleotidyl transferase dUTP nick-end labeling (TUNEL)–stained sections of heart tissue from mice euthanized 1 and 3 days after IR injury. Scale bar=100 µm. **E**, Fibrosis was evaluated in Masson trichrome–stained heart sections from mice euthanized 28 days after IR injury, quantified as the ratio of the length of fibrosis (blue) to the LV circumference, and presented as a percentage. **F** through **H**, Echocardiographic assessments of (**F**) LV fractional shortening (FS), (**G**) LV systolic area (LVAs), and (**H**) LV diastolic area (LVAd) were performed before IR injury and 7 to 28 days afterward; heart rates were maintained at 400 to 500 bpm via isoflurane inhalation. #*P*<0.05 and ##*P*<0.01 vs before injection; **B** and **C**, n=3 per treatment group; **D**, n=3 to 5 per treatment group; **E**, n=6 to 9 per treatment group; **F** through **H**, n=10 per treatment group at each time point. a-SA indicates α-sarcomeric actin; and HPF, high-power field. **P*<0.05, ***P*<0.01, §Bonferroni-adjusted *P*<0.05 and §§Bonferroni-adjusted *P*<0.01 vs saline.

histological and echocardiographic assessments suggest that a single injection of AMD3100 after IR injury improves cardiac performance by enhancing the preservation and/or recovery of functional myocardial tissue.

AMD3100 Preferentially Enhances the Mobilization of BM Cells After IR Injury

Because AMD3100 is a CXCR4 antagonist and has been shown to enhance the mobilization of stem/progenitor cells from BM to PB after permanent ligation of the coronary artery,[17] we investigated whether AMD3100 treatment enhanced the mobilization of MNCs, CXCR4$^+$ MNCs, and sca1$^+$/flk1$^+$ MNCs in both uninjured mice and mice with IR injury. PB levels of the 3 types of cells were measured via fluorescence-activated cell sorting, and fluorescence-activated cell sorting measurements of sca1$^+$/flk1$^+$ MNC levels were corroborated via the EPC culture assay. Tie2$^+$ BM progenitor mobilization was also evaluated by monitoring green fluorescent protein (GFP) expression in the BM of wild-type (WT) mice transplanted with BM from Tie2-GFP transgenic mice, which express GFP from the endothelium-specific Tie2 promoter.

In the absence of injury, fluorescence-activated cell sorting analyses indicated that PB levels of MNCs, including sca1$^+$/flk1$^+$ subpopulation, tended to increase (Figure 2A and C) and CXCR4$^+$ MNCs significantly increased after AMD3100 treatment (Figure 2B). The time course of mobilization in the 3 cell types was almost similar: Cell counts tended to peak within 3 hours after AMD3100 administration and returned to near pretreatment levels by 24 hours (Figure 2A–2C). After IR injury, AMD3100 treatment did not alter PB MNC levels (Figure 2D), but PB CXCR4$^+$ MNC counts (Figure 2E) and sca1$^+$/flk1$^+$ MNC counts (Figure 2F) were significantly higher 1 and 3 days, respectively, after injury in AMD3100-treated mice than in mice administered saline. The enhanced mobilization of CXCR4$^+$ MNCs diminished by day 3, whereas PB sca1$^+$/flk1$^+$ MNC counts remained significantly higher in the AMD3100-treated mice than in the saline-treatment group through day 7. When evaluated via culture assay, PB EPC levels (ie, the number of cells stained positively for both lectin and acetylated low-density lipoprotein) were significantly higher in the AMD3100-treated mice than in the saline-treated mice on days 3 and 7 after IR injury (Figure I in the

Downloaded from http://ahajournals.org by on April 30, 2021

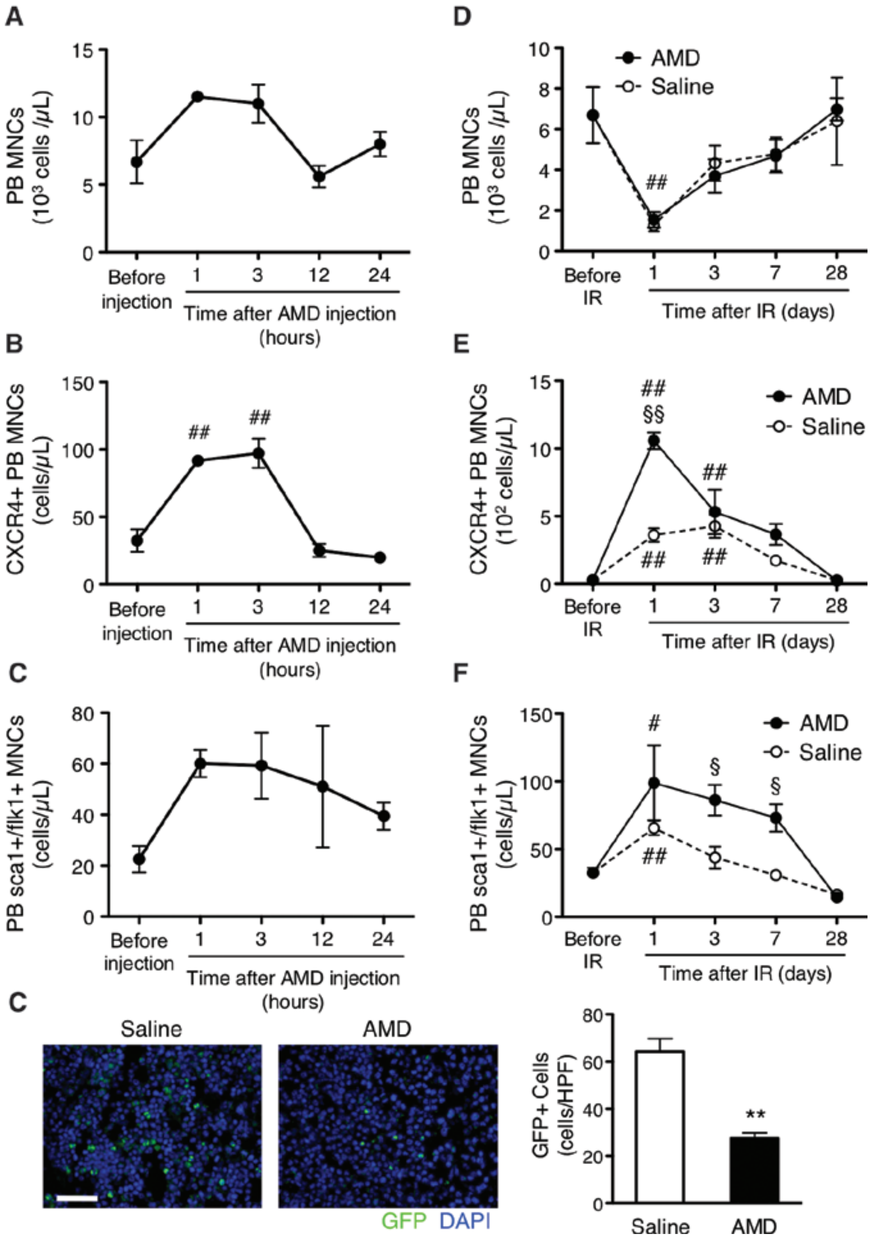

Figure 2. AMD3100 enhances the mobilization of circulating CXC-chemokine receptor 4–positive (CXCR4⁺) mononuclear cells (MNCs) and Sca1⁺/Flk1⁺ cells after ischemia/reperfusion (IR) injury. **A** through **C**, Peripheral blood (PB) levels of (**A**) MNCs, (**B**) CXCR4⁺ MNCs, and (**C**) sca1⁺/flk1⁺ cells were determined in uninjured mice before injection of AMD3100 (125 µg in100 µL saline) and from 1 to 24 hours afterward. **D** through **F**, PB levels of (**D**) MNCs, (**E**) CXCR4⁺ MNCs, and (**F**) sca1⁺/flk1⁺ cells were determined in mice before IR injury and treatment with AMD3100 or saline and from 1 to 28 days afterward. MNC levels were measured with a Hema-Vet hematology system, and the levels of CXCR4⁺ MNCs and sca1⁺/flk1⁺ cells were measured via fluorescence-activated cell sorter analyses of MNCs labeled with fluorescent CXCR4 antibodies (CXCR4⁺ MNCs) or double-labeled with fluorescent Sca1 antibodies and Flk1 antibodies. **G**, IR injury was surgically induced in wild-type mice that had been transplanted with bone marrow (BM) from mice with Tie2-regulated green fluorescent protein (GFP) expression. Mice were treated with AMD3100 or saline after injury, and the number of GFP⁺ BM cells was determined 5 days later. Scale bar=100 µm. **A** through **C**, n=3; **D** through **F**, n=3 to 5 per treatment group at each time point; **G**, n=8 per treatment group. The SEMs are too small to be visible graphically in **A**, hour 1; **B**, hours 1 and 24; **E**, before IR, day 7 (saline), and day 28; and **F**, day 7 (saline) and day 28. #Bonferroni-adjusted $P<0.05$ and ##Bonferroni-adjusted $P<0.01$ vs before injection/IR; §Bonferroni-adjusted $P<0.05$, §§Bonferroni-adjusted $P<0.01$ and $^{**}P<0.01$ vs saline.

Downloaded from http://ahajournals.org by on April 30, 2021

online-only Data Supplement), and in mice with Tie2-GFP BM, GFP-expressing cells were significantly less common in BM from the AMD3100-treated group than in BM from saline-treated animals on day 5 after IR injury (Figure 2G). Thus, AMD3100 appears to rapidly but briefly enhance the mobilization of CXCR4⁺ MNCs after IR injury, which is consistent with the role of AMD3100 as a CXCR4 antagonist. However, the effect of AMD3100 on sca1⁺/flk1⁺ MNC mobilization is delayed, more durable, and consequently likely mediated by a different mechanism.

AMD3100 Increases the Contribution of BM-Derived Progenitors to Vascular Growth After IR Injury

To determine whether the enhanced BMPC mobilization observed in mice treated with AMD3100 after IR injury was accompanied by improved vascularity in the AAR, the functional vasculature of injured mice was stained via in vivo perfusion with BS1-lectin before the mice were killed. Experiments were performed in WT mice transplanted with BM from enhanced GFP–expressing mice to enable identification of BM-derived cells in the vasculature. Compared with observations in saline-treated mice, the AAR of AMD3100-treated mice contained significantly more GFP⁺ cells on day 3 after injury (Figure 3A and 2B) and significantly more GFP⁺ cells, GFP-lectin double-positive cells (Figure 3C and 3D), and lectin⁺ vessel density (Figure 3C and 3E) on day 28. AMD3100 treatment also kept elevated SDF-1 expression levels in AAR through day 3 to 7 after IR injury in mice with WT BM (Figure II in the online-only Data Supplement), which likely contributed to the enhanced incorporation of BM-derived cells, perhaps including EPCs.

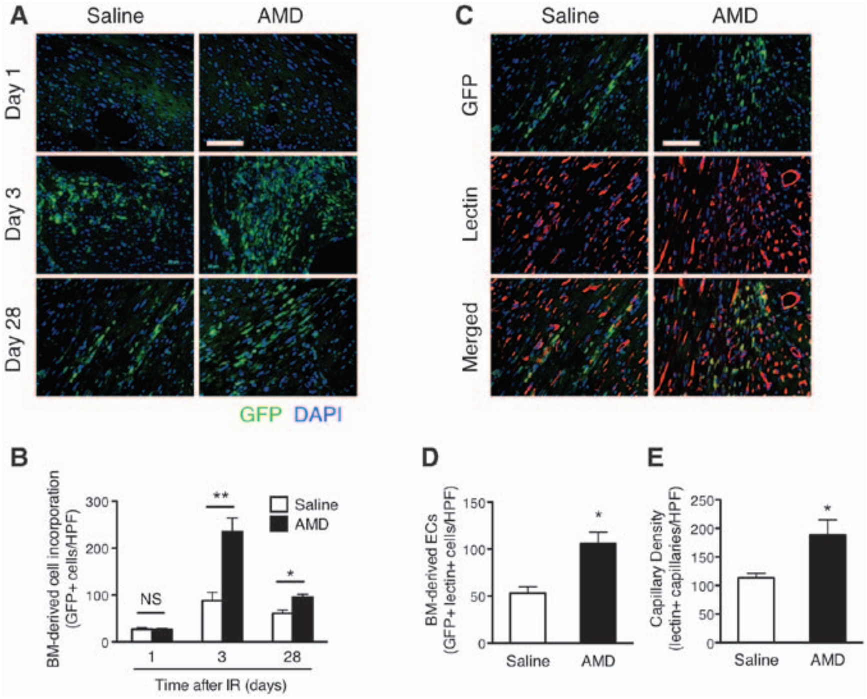

Figure 3. AMD3100 increases capillary density and the number of bone marrow (BM)–derived endothelial cells in the myocardium after ischemia/reperfusion (IR) injury. AMD3100 or saline was subcutaneously injected after IR injury in wild-type mice that had been transplanted with BM from green fluorescent protein (GFP)–expressing mice. **A** and **B**, BM-derived (ie, GFP-expressing) cells (green) were identified in the area at risk (AAR) and quantified on days 1, 3, and 28 after injury. Scale bar=100 μm. **C** through **E**, On day 28 after IR injury, mice were perfused with BS1-lectin before death, and sections from the AAR were stained with fluorescent anti-lectin antibodies. **C**, BM-derived endothelial cells (ECs) were quantified as the number of cells positive for both GFP expression and lectin staining. Scale bar=100 μm. **D**, BM-derived cells (green, GFP fluorescence) and functional vascular structures (red, lectin fluorescence) were identified in the AAR, and (**E**) capillary density was quantified as the number of lectin$^+$ vascular structures. **B**, n=3 to 6 per treatment group at each time point; **D** and **E**, n=3 to 4 per treatment group. **P*<0.05 and ***P*<0.01 vs saline.

AMD3100 Increases BM Endothelial Nitric Oxide Synthase Expression and the Number of Endothelial Nitric Oxide Synthase–Expressing BM-Derived Cells in the AAR After IR Injury

Endothelial nitric oxide synthase (eNOS) is a key regulator of endothelial cell growth and migration, vascular remodeling, and angiogenesis[21–23] and has recently been shown to have an important role in the activity of stem and progenitor cells. We investigated whether the enhanced functional recovery and BMPC mobilization associated with AMD3100 administration after IR injury are accompanied by increases in eNOS activity.

From day 1 through day 7 after IR injury, eNOS-expressing cells were significantly more common in the BM of AMD3100-treated mice than in the BM of mice administered saline (Figure 4A). AMD3100 treatment was also associated with higher BM protein levels of matrix metalloproteinase-9 and soluble Kit ligand,[24] 2 downstream components of the eNOS pathway, from day 1 and 3, respectively, through day 7 (Figure 4B and 4C) and with higher PB levels of nitrate and nitrite (ie, the final metabolites of nitric oxide) on day 3 (Figure 4D). In mice transplanted with BM from transgenic GFP-expressing mice, the number of cells in the AAR that expressed eNOS, GFP, or both eNOS and GFP was significantly higher in AMD3100-treated mice than in saline-treated mice on day 3 after injury (Figure 4E–4H). Thus, AMD3100 administration after IR injury appears to increase eNOS activity in both the BM and the ischemic region.

The direct influence of AMD3100 on eNOS activity was investigated by determining whether AMD3100 treatment altered eNOS mRNA expression and nitrate/nitrite production in cultured BMPCs or luciferase activity in murine endothelial cells transfected with a gene coding for luciferase expression from the eNOS promoter. AMD3100 treatment was associated with higher levels of both eNOS expression and nitrate/nitrite production in BMPCs (Figure IIIA and IIIB in the online-only Data Supplement), and AMD3100 dose-dependently increased luciferase activity in transfected endothelial cells (Figure IIIC in the online-only Data Supplement). Collectively, these observations suggest that the benefits associated with AMD3100 administration are accompanied by increases in eNOS activity.

The Benefit of AMD3100 Treatment After IR Injury Is Dependent on eNOS Expression in the BM but Not in the Ischemic Region

To determine whether eNOS expression contributes to the benefits associated with AMD3100 administration after IR injury and, if so, whether that contribution comes from BM cells or from cells already present in the ischemic tissue, we evaluated the influence of AMD3100 on myocardial recovery in eNOS-knockout mice that had been transplanted with BM from WT mice (eNOS-KO/WT_{BM}) and in WT mice transplanted with BM from eNOS-KO mice (WT/eNOS-KO_{BM}). An identical set of assessments was performed in WT mice transplanted with WT BM (WT/WT_{BM}).

In eNOS-KO/WT_{BM} mice, LV fractional shortening on days 14 and 28 after IR injury was significantly greater with AMD3100 treatment than with saline treatment, but the functional benefit of AMD3100 treatment was not observed in WT/eNOS-KO_{BM} mice at any time point (Figure 5A and 5B and Table II in the online-only Data Supplement). Similarly, AMD3100 treatment in eNOS-KO/WT_{BM} mice but not in WT/eNOS-KO_{BM} mice was associated with greater numbers of eNOS$^+$ BM cells, elevated BM matrix metalloproteinase-9 and soluble Kit ligand protein expression, higher PB sca1$^+$/flk1$^+$ MNC counts, less cardiac apoptosis, and smaller infarcts on day 3 after injury (Figure 5D–5I) and with less cardiac

Downloaded from http://ahajournals.org by on April 30, 2021

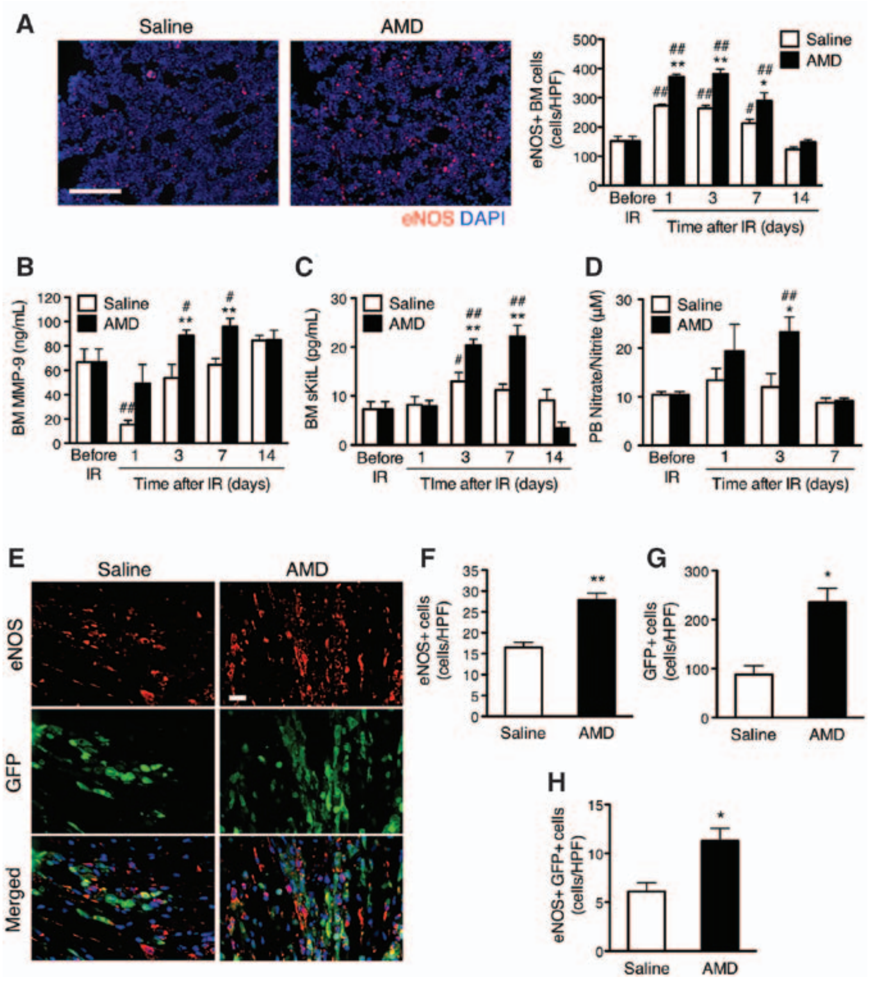

Figure 4. AMD3100 increases bone marrow (BM) endothelial nitric oxide synthase (eNOS) expression and the number of eNOS-expressing BM-derived cells in the area at risk (AAR) after ischemia/reperfusion (IR) injury. AMD3100 or saline was subcutaneously injected after IR injury in (**A**–**D**) wild-type (WT) mice and (**E**–**H**) WT mice that had been transplanted with BM from green fluorescent protein (GFP)–expressing mice. **A** through **C**, BM was harvested from mice euthanized before IR injury and from 1 to 14 days afterward. **A**, BM cells were labeled with fluorescent anti-eNOS antibodies (red), and $eNOS^{+}$ cells were quantified. Scale bar=100 µm. **B** and **C**, BM plasma levels of (**B**) matrix metalloproteinase-9 (MMP-9) and (**C**) soluble Kit ligand (sKitL) protein were determined via ELISA. **D**, Peripheral blood (PB) levels of nitrate and nitrite were determined via colorimetric assay before IR injury and 1 to 14 days afterward. **E**, eNOS (red) and GFP (green) expression was evaluated in sections from the AAR of mice euthanized 3 days after IR injury. Scale bar=20 µm. Cells positive for the expression of (**F**) eNOS, (**G**) GFP, or (**H**) both eNOS and GFP were quantified. **A** through **D**, n=4 to 8 per treatment group at each time point; **F** through **H**, n=3 to 5 per treatment group. HPF indicates high-power field. #$P<0.05$ and ##$P<0.01$ vs before injection; *$P<0.05$ and **$P<0.01$ vs saline.

fibrosis and greater capillary density on day 28 (Figure 5J and 5K). The results associated with AMD3100 treatment in WT/WT_{BM} mice matched those observed in eNOS-KO/WT_{BM} mice (Figure 5C–5K), and the only treatment-related effect observed in all 3 chimeric mouse lines was the enhanced mobilization of $CXCR4^{+}$ MNCs, which occurred on day 1 after injury and diminished by day 3 (Figure 5L). Thus, the benefits associated with AMD3100 administration after IR injury require eNOS expression in the BM but not in the ischemic region, and eNOS appears to have a role in the mobilization of $sca1^{+}/flk1^{+}$ MNCs but not $CXCR4^{+}$ MNCs.

The Expression of eNOS by PB EPCs Contributes to Myocardial Recovery but Is Not Required for EPC Incorporation

Because BM eNOS expression is required for both the beneficial effects of AMD3100 treatment after IR injury and the mobilization of progenitors from BM to PB, we investigated whether eNOS expression in circulating BMPCs contributes to myocardial recovery. BMPCs were isolated from WT mice and eNOS-KO mice, cultured for 4 days, and then intravenously injected into WT mice 24 hours after IR injury; a third group of mice were injected with saline. Fourteen and 28 days after IR injury, LV fractional shortening was significantly greater in mice administered WT BMPCs than in mice administered saline, but the difference between treatment with eNOS-KO BMPCs and saline administration did not reach statistical significance (Figure 6A and Table II in the online-only Data Supplement).

To determine whether the expression of eNOS by circulating BMPCs contributes to myocardial recovery by increasing the incorporation and whether AMD3100 enhances the incorporation of circulating BMPCs, WT and eNOS-KO EPCs were labeled with DiI and intravenously injected into WT mice 24 hours after IR injury and treatment with AMD3100 or saline. Mice were euthanized 3 days after IR injury for histological analyses. Neither the type of cell injected (ie, eNOS-KO or WT) nor the treatment administered (ie, AMD3100 or saline) significantly influenced the incorporation of injected cells (Figure 6B). Nevertheless, apoptotic cells were significantly less common and infarct sizes were significantly smaller in mice administered WT BMPCs than in mice administered eNOS-KO BMPCs, regardless of treatment group, and measurements in mice administered eNOS-KO BMPCs did not differ significantly from those in saline-treated mice (Figure 6C and 6D).

Collectively, these observations suggest that the expression of eNOS by circulating BMPCs does not have a role in their recruitment and incorporation but does contribute to cardiac protection. Therefore, the greater number of BM-derived endothelial cells observed in the AAR of AMD3100-treated mice (Figure 3C) appears to evolve primarily through

Downloaded from http://ahajournals.org by on April 30, 2021

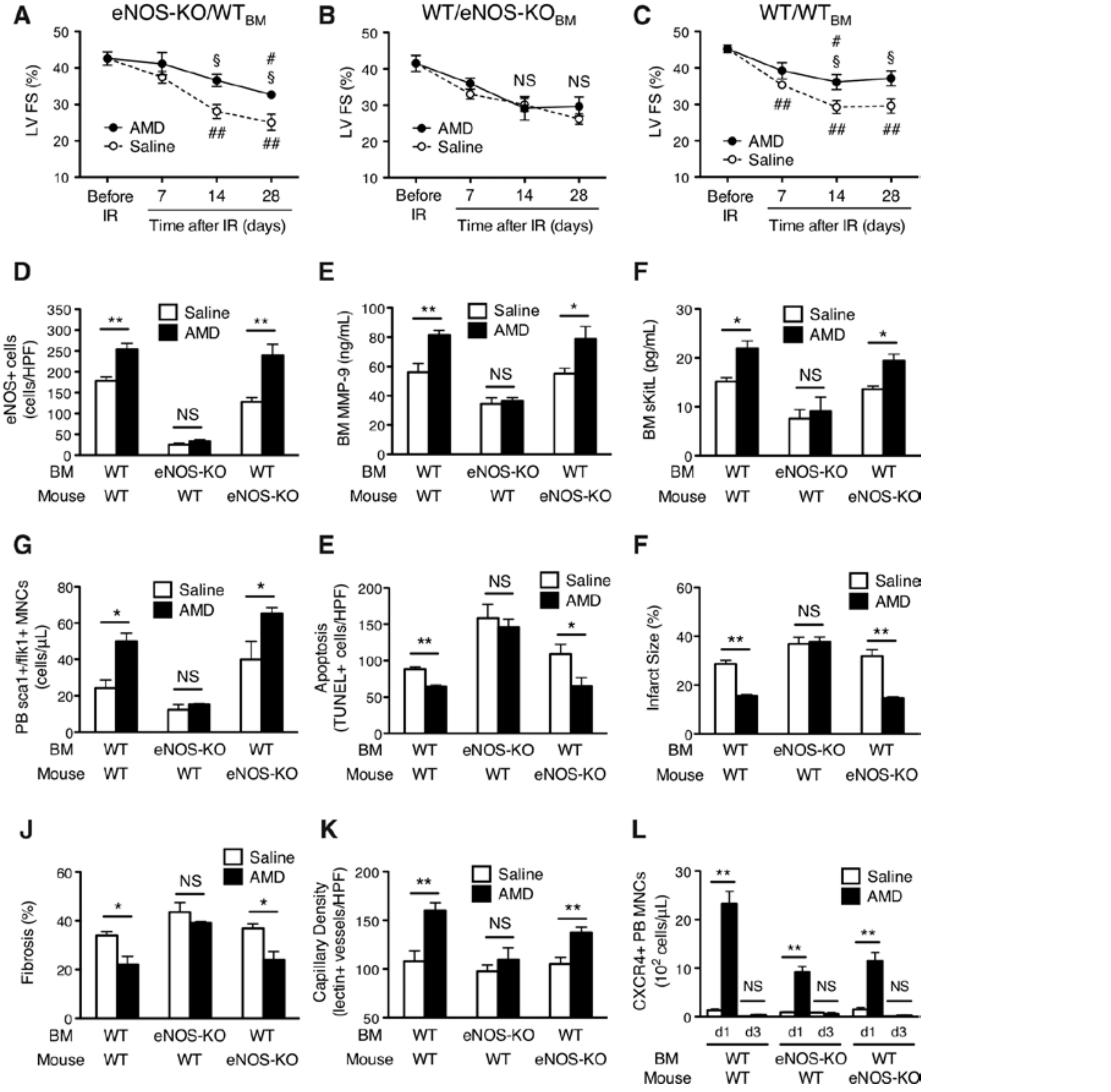

Figure 5. The benefit of AMD3100 treatment after ischemia/reperfusion (IR) injury is dependent on endothelial nitric oxide synthase (eNOS) expression in the bone marrow (BM) but not in the ischemic tissue. AMD3100 or saline was subcutaneously injected after IR injury in eNOS-knockout mice that had been transplanted with BM from wild-type (WT) mice (eNOS-KO/WT$_{BM}$), in WT mice transplanted with BM from eNOS-KO mice (WT/eNOS-KO$_{BM}$), and in WT mice transplanted with WT BM (WT/WT$_{BM}$). **A** through **C**, Echocardiographic assessments of left ventricular fractional shortening (LV FS) were performed before IR injury and 7 to 28 days afterward. **D** through **F**, BM was harvested 3 days after IR injury. **D**, BM cells were labeled with fluorescent anti-eNOS antibodies, and positively stained cells were quantified. **E** and **F**, BM plasma levels of (**E**) matrix metalloproteinase-9 (MMP-9) and (**F**) soluble Kit ligand (sKitL) protein were determined via ELISA. **G**, Three days after IR injury, mononuclear cells (MNCs) were isolated from the peripheral blood (PB) and labeled with fluorescent anti-Sca1 and anti-Flk1 antibodies, and the number of MNCs positive for both Sca1 and Flk1 expression was determined via fluorescence-activated cell sorting (FACS) analysis. **H**, Sections from the area at risk (AAR) of mice euthanized on day 3 after IR injury were stained with terminal deoxynucleotidyl transferase dUTP nick-end labeling (TUNEL), and apoptosis was quantified as the number of positively stained cells. **I**, Infarct size and AAR were assessed via in vivo microsphere perfusion and triphenyltetrazolium chloride staining in mice euthanized on day 3 after IR injury; the ratio of the area of the infarct to the AAR was presented as a percentage. **J**, Fibrosis on day 28 after IR injury was assessed in Masson trichrome–stained heart sections, quantified as the ratio of the length of fibrosis to the left ventricular circumference, and presented as a percentage. **K**, Capillary density was assessed in mice that had been perfused with BS1-lectin before death on day 28 after IR injury and quantified as the number of lectin$^+$ vascular structures. **L**, MNCs were harvested from the PB on day 1 (d1) and day 3 (d3) after IR injury and labeled with fluorescent anti-CXCR4 antibodies, and the number of CXCR4$^+$ MNCs was determined via FACS. **A** through **C**, n=4 to 8 per treatment group at each time point; **D**, n=3 to 4 per treatment group; **E**, n=4 to 8 per treatment group; **F**, n=5 to 9 per treatment group; **G** through **J**, n=3 to 6 per treatment group; **K**, n=3 per treatment group; **L**, n=3 to 6 per treatment group at each time point. The SEM is too small to be visible graphically for **C**, day 7 (saline). HPF indicates high-power field. ##Bonferroni-adjusted *P*<0.01 vs before injection; §Bonferroni-adjusted *P*<0.05, **P*<0.05, and ***P*<0.01 vs saline.

enhanced BMPC mobilization, which subsequently increases the number of the progenitors available in the circulation, rather than by directly influencing the recruitment and incorporation of circulating cells.

Discussion

In the present study, we have shown that AMD3100, a CXCR4 antagonist, had a beneficial effect on cardiac IR injury that closely mimics coronary intervention in acute myocardial

Downloaded from http://ahajournals.org by on April 30, 2021

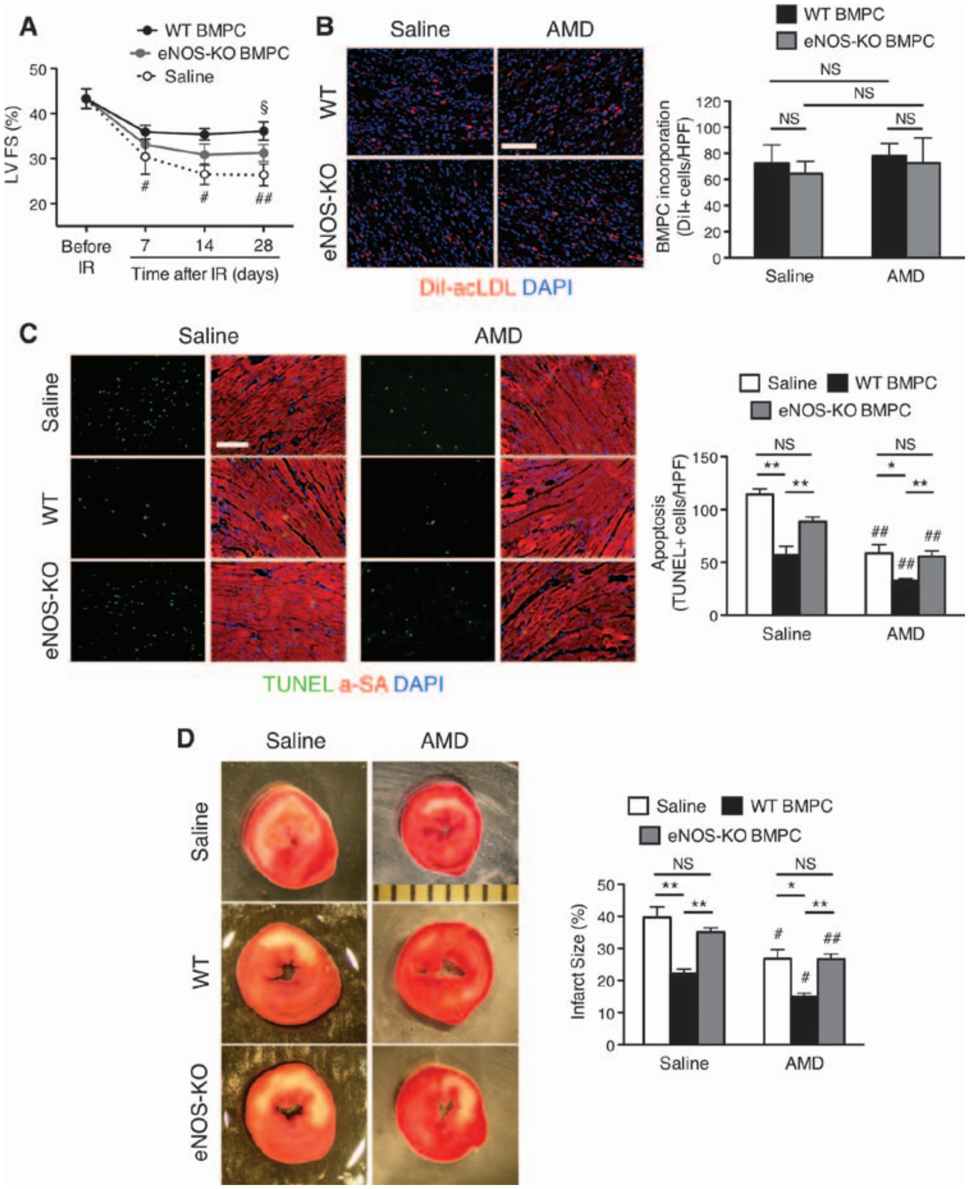

Figure 6. The expression of endothelial nitric oxide synthase (eNOS) by circulating bone marrow progenitor cells (BMPCs) contributes to myocardial recovery. **A**, BMPCs from wild-type (WT) mice, BMPCs from eNOS-knockout (KO) mice, or saline was intravenously injected into WT mice 24 hours after ischemia/reperfusion (IR) injury. Echocardiographic assessments of left ventricular fractional shortening (LV FS) were performed before IR injury and 7 to 28 days afterward. **B** through **D**, DiI-labeled WT BMPCs, DiI-labeled eNOS-KO BMPCs, or saline was intravenously injected into WT mice 24 hours after IR injury, along with treatment with subcutaneous injections of AMD3100 or saline. Mice were euthanized 2 days later (ie, 3 days after IR injury). §Bonferroni-adjusted $P<0.05$ vs saline; #$P<0.05$ and ##$P<0.01$ vs before injection. **B**, Incorporation of the injected cells was evaluated by quantifying the number of DiI-positive cells in the area at risk (AAR). Scale bar=100 µm. **C**, Sections from the AAR were stained with terminal deoxynucleotidyl transferase dUTP nick-end labeling (TUNEL; green), stained with fluorescent anti–α-sarcomeric actin (a-SA) antibodies (red), and counterstained with DAPI (blue). Scale bar=100 µm. Apoptosis was quantified as the number of TUNEL$^+$ cells. **D**, Infarct size and AAR were assessed via in vivo microsphere perfusion and triphenyltetrazolium chloride staining; viable tissue appears deep red and the infarcted region is colorless. The ratio of the area of the infarct to the AAR was presented as a percentage. *$P<0.05$ and **$P<0.01$, $^{\#}P<0.05$ and $^{\#\#}P<0.01$ vs the same intravenous injection (ie, saline, WT BMPCs, or eNOS-KO BMPCs) in animals treated with subcutaneous injections of saline. **A**, n=4 to 5 per treatment group at each time point; **B**, n=3 to 5 per group; **C**, n=3 to 7 per group; **D**, n=4 to 7 per group. acLDL indicates acetylated low-density lipoprotein; and HPF, high-power field.

infarction patients. AMD3100 enhanced the mobilization of BM-derived progenitor cells, including EPCs, that were incorporated into AAR and exerted cardioprotective effects by antiapoptosis and anti-inflammation in the acute phase and revascularizing effects in the remote phase, resulting in minimization of scar size and preservation of cardiac functions.

AMD3100 has been approved by the US Food and Drug Administration for use as a stem cell mobilizing agent[25]; however, few reports have evaluated the time course and subpopulations of cells mobilized by AMD3100 administration after IR injury. Our results indicate that rapid accumulation of circulating MNCs, specifically inflammatory cells, into AAR accounts for the decreased number of MNCs in PB on day 1

Downloaded from http://ahajournals.org by on April 30, 2021

after IR injury. On the other hand, IR injury could be a trigger to mobilize progenitors (ie, $CXCR4^+$ or $sca\text{-}1^+/flk1^+$ MNCs, including EPCs) from BM, resulting in the increased number of progenitors in PB. AMD3100 increased the mobilization of $CXCR4^+$ cells on day 1 after IR injury by disrupting SDF-1–CXCR4 binding in BM, but this enhancement dissipated before the expression of SDF-1, a ligand of CXCR4, was upregulated in ischemic myocardium on day 3 after IR injury. The delay in SDF-1 upregulation may explain why the rapid mobilization of $CXCR4^+$ cells did not result in a dramatic increase in BM-derived cell recruitment to the AAR on day 1 after IR injury. The AMD3100-induced delayed enhancement of $sca1^+/flk1^+$ MNC mobilization coincided with elevated BM levels of eNOS and 2 eNOS-targeted proteins, matrix metalloproteinase-9 and soluble Kit ligand, that have been linked to progenitor cell mobilization,[24,26] and the enhanced mobilization of progenitors required BM eNOS expression.

eNOS is known to protect cardiomyocytes against apoptosis,[27,28] and AMD3100 treatment led to increases in the number of eNOS-expressing cells, to declines in the number of apoptotic cells, and to a reduction in infarct sizes in AAR on day 3 after IR injury. Similarly, systemically injected eNOS-expressing WT BMPCs but not eNOS-KO BMPCs were associated with less apoptosis and smaller infarct sizes in both AMD3100-treated and saline-treated mice on day 3 after injury, even though the number of recruited cells in both cell types in the AAR was similar. Additionally, in IR-injured mice without AMD3100 treatment, eNOS expression in BM was upregulated by myocardial transient ischemia alone from day 1 through 7; therefore, AMD3100 was assumed to give an extra elevation of eNOS production in BM cells over ischemic insult.

In the mechanistic aspect, it was reported that eNOS-expressing cells enhanced vascular endothelial growth factor (VEGF) protein production.[29] As known broadly, VEGF upregulates eNOS expression, suggesting that initial upregulation of eNOS expression was sustained up to day 7 after IR injury with AMD3100 treatment via an autocrine mechanism involving the VEGF-eNOS signaling pathway. We previously showed that VEGF played a crucial role in mobilizing progenitors from BM into PB in a mouse coronary ligation model. However, in the present study, we confirmed a direct effect of AMD3100 on eNOS upregulation by reporter assay (Figure IIIC in the online-only Data Supplement). In addition, eNOS mRNA expression was upregulated >2-fold in the AMD3100-treated BMPCs compared with control BM PCs, whereas VEGF mRNA expression was limited to a <2-fold increase in the AMD3100-treated group (Figure IV in the online-only Data Supplement). Thus, AMD3100 could directly enhance eNOS production in the recruited cultured BMPCs in ischemic myocardium, suggesting that AMD3100 might exhibit a cardioprotective effect via the recruited BMPC-derived eNOS production, at least in part, in the WT and eNOS-KO BMPC injection experiment. In addition, on the basis of the evidence that eNOS promoted angiogenesis and reduced apoptosis by inhibition of transforming growth factor-β1 signaling,[30] imported BMPC-derived eNOS may contribute to enhance capillary density after IR injury via a similar mechanism. Thus, the cardioprotective effects of AMD3100-induced eNOS activity are a crucial component of the response to IR injury, but these effects appear to evolve primarily from BM-derived progenitors rather than from cells already present in the AAR.

In terms of the contribution of the mobilized BM-derived progenitors by AMD3100 to ischemic myocardium, although there is no direct evidence that circulating $CXCR4^+$ cells incorporated into the AAR in the acute phase after IR, the peak number of mobilized $CXCR4^+$ cells was striking (10 times more than that of mobilized $sca1^+/flk1^+$ cells); therefore, $CXCR4^+$ cells may play a role in the AAR. Referring to the reports in which CXCR4-expressing cells were shown to enhance incorporation into ischemic area and to improve cardiac function after myocardial infarction[31,32] and the fact that the plasma half-life of AMD3100 is very short (3.5 hours) in circulation, mobilized $CXCR4^+$ cells might not be affected by its recruitment to SDF-1–releasing sites of ischemia at day 1 to 3 after IR injury. Even though the eNOS KO- or WT-cultured BMPC infusion study did not show significant differences in cultured BMPC recruitment to sites of ischemia, endogenous progenitors, namely $CXCR4^+$ cells and $sca1^+/flk1^+$ cells mobilized by AMD3100, might recruit to ischemic myocardium, exhibiting cardioprotective effects, because SDF-1 and VEGF are released from ischemic myocardium and recruit circulating progenitors expressing receptors of CXCR4[31] and flk1.[33]

The findings presented here indicate that AMD3100 improves the recovery of cardiac function after IR injury and that the beneficial effect of AMD3100 on ischemic heart with IR injury requires eNOS expression in BM but not in myocardium. AMD3100 sustains the mobilization of $sca1^+/flk1^+$ MNCs rather than $CXCR4^+$ MNCs, resulting in the increased number of recruited BM-derived eNOS-expressing cells, and contributes to limiting infarct size, reducing cardiac apoptosis, and increasing vascularity in ischemic myocardium. Collectively, single treatment with AMD3100 may give rise to a novel supportive therapy in percutaneous transluminal coronary angioplasty and stenting for acute coronary syndrome via a cardioprotection/proangiogenesis-dependent mechanism.

Study Limitations

In this study, we have not definitively identified the mobilized BM-derived cells by AMD3100, including $sca1^+/flk1^+$ MNCs and $CXCR4^+$ cells, providing direct evidence for the incorporation into vascular structure, however, we and others have previously demonstrated that not the majority but a certain extent of these BM-derived cells were exactly incorporated into neovasculature in ischemic tissue.[31,32,34–36] In addition, because we have focused on the therapeutic effect of AMD3100 with its eNOS-dependent BMPC mobilization in PB and the recruitment to sites of IR injury, we have not tried to identify what type of cells, so-called EPCs, defined by multiple cell surface markers are incorporated into the neovasculature. The precise definition of human/mouse EPCs remains unclear and would be quite difficult to ascertain.

Acknowledgments

We thank Kari Krueger for administrative assistance and W. Kevin Meisner, PhD, ELS, for editorial support. The work presented in this article was performed at the Feinberg Cardiovascular Research

Downloaded from http://ahajournals.org by on April 30, 2021

Institute, Northwestern University Feinberg School of Medicine, Chicago, IL.

Sources of Funding

This work was supported in part by the US National Institutes of Health (grants HL053354-14, HL057516-12, HL080137-05, HL095874-04, HL093439-03, and HL113541-01).

Disclosures

Dr Losordo is an employee of Baxter Healthcare. The other authors report no conflicts.

References

1. Murasawa S, Kawamoto A, Horii M, Nakamori S, Asahara T. Niche-dependent translineage commitment of endothelial progenitor cells, not cell fusion in general, into myocardial lineage cells. *Arterioscler Thromb Vasc Biol*. 2005;25:1388–1394.
2. Shintani S, Murohara T, Ikeda H, Ueno T, Honma T, Katoh A, Sasaki K, Shimada T, Oike Y, Imaizumi T. Mobilization of endothelial progenitor cells in patients with acute myocardial infarction. *Circulation*. 2001;103:2776–2779.
3. Brenner W, Aicher A, Eckey T, Massoudi S, Zuhayra M, Koehl U, Heeschen C, Kampen WU, Zeiher AM, Dimmeler S, Henze E. 111In-labeled CD34+ hematopoietic progenitor cells in a rat myocardial infarction model. *J Nucl Med*. 2004;45:512–518.
4. Mohle R, Bautz F, Rafii S, Moore MA, Brugger W, Kanz L. The chemokine receptor CXCR-4 is expressed on CD34+ hematopoietic progenitors and leukemic cells and mediates transendothelial migration induced by stromal cell-derived factor-1. *Blood*. 1998;91:4523–4530.
5. Peled A, Grabovsky V, Habler L, Sandbank J, Arenzana-Seisdedos F, Petit I, Ben-Hur H, Lapidot T, Alon R. The chemokine SDF-1 stimulates integrin-mediated arrest of CD34(+) cells on vascular endothelium under shear flow. *J Clin Invest*. 1999;104:1199–1211.
6. Yamaguchi J, Kusano KF, Masuo O, Kawamoto A, Silver M, Murasawa S, Bosch-Marce M, Masuda H, Losordo DW, Isner JM, Asahara T. Stromal cell-derived factor-1 effects on ex vivo expanded endothelial progenitor cell recruitment for ischemic neovascularization. *Circulation*. 2003;107:1322–1328.
7. Aiuti A, Webb IJ, Bleul C, Springer T, Gutierrez-Ramos JC. The chemokine SDF-1 is a chemoattractant for human CD34+ hematopoietic progenitor cells and provides a new mechanism to explain the mobilization of CD34+ progenitors to peripheral blood. *J Exp Med*. 1997;185:111–120.
8. Wang JF, Liu ZY, Groopman JE. The alpha-chemokine receptor CXCR4 is expressed on the megakaryocytic lineage from progenitor to platelets and modulates migration and adhesion. *Blood*. 1998;92:756–764.
9. Lataillade JJ, Clay D, Dupuy C, Rigal S, Jasmin C, Bourin P, Le Bousse-Kerdiles MC. Chemokine SDF-1 enhances circulating CD34(+) cell proliferation in synergy with cytokines: possible role in progenitor survival. *Blood*. 2000;95:756–768.
10. Este JA, Cabrera C, De Clercq E, Struyf S, Van Damme J, Bridger G, Skerlj RT, Abrams MJ, Henson G, Gutierrez A, Clotet B, Schols D. Activity of different bicyclam derivatives against human immunodeficiency virus depends on their interaction with the CXCR4 chemokine receptor. *Mol Pharmacol*. 1999;55:67–73.
11. Gerlach LO, Skerlj RT, Bridger GJ, Schwartz TW. Molecular interactions of cyclam and bicyclam non-peptide antagonists with the CXCR4 chemokine receptor. *J Biol Chem*. 2001;276:14153–14160.
12. Hatse S, Princen K, Bridger G, De Clercq E, Schols D. Chemokine receptor inhibition by AMD3100 is strictly confined to CXCR4. *FEBS Lett*. 2002;527:255–262.
13. Hendrix CW, Flexner C, MacFarland RT, Giandomenico C, Fuchs EJ, Redpath E, Bridger G, Henson GW. Pharmacokinetics and safety of AMD-3100, a novel antagonist of the CXCR-4 chemokine receptor, in human volunteers. *Antimicrob Agents Chemother*. 2000;44:1667–1673.
14. Broxmeyer HE, Orschell CM, Clapp DW, Hangoc G, Cooper S, Plett PA, Liles WC, Li X, Graham-Evans B, Campbell TB, Calandra G, Bridger G, Dale DC, Srour EF. Rapid mobilization of murine and human hematopoietic stem and progenitor cells with AMD3100, a CXCR4 antagonist. *J Exp Med*. 2005;201:1307–1318.
15. Liles WC, Broxmeyer HE, Rodger E, Wood B, Hubel K, Cooper S, Hangoc G, Bridger GJ, Henson GW, Calandra G, Dale DC. Mobilization of hematopoietic progenitor cells in healthy volunteers by AMD3100, a CXCR4 antagonist. *Blood*. 2003;102:2728–2730.
16. Devine SM, Flomenberg N, Vesole DH, Liesveld J, Weisdorf D, Badel K, Calandra G, DiPersio JF. Rapid mobilization of CD34+ cells following administration of the CXCR4 antagonist AMD3100 to patients with multiple myeloma and non-Hodgkin's lymphoma. *J Clin Oncol*. 2004;22:1095–1102.
17. Jujo K, Hamada H, Iwakura A, Thorne T, Sekiguchi H, Clarke T, Ito A, Misener S, Tanaka T, Klyachko E, Kobayashi K, Tongers J, Roncalli J, Tsurumi Y, Hagiwara N, Losordo DW. CXCR4 blockade augments bone marrow progenitor cell recruitment to the neovasculature and reduces mortality after myocardial infarction. *Proc Natl Acad Sci USA*. 2010;107:11008–11013.
18. Asahara T, Masuda H, Takahashi T, Kalka C, Pastore C, Silver M, Kearne M, Magner M, Isner JM. Bone marrow origin of endothelial progenitor cells responsible for postnatal vasculogenesis in physiological and pathological neovascularization. *Circ Res*. 1999;85:221–228.
19. Asahara T, Takahashi T, Masuda H, Kalka C, Chen D, Iwaguro H, Inai Y, Silver M, Isner JM. VEGF contributes to postnatal neovascularization by mobilizing bone marrow-derived endothelial progenitor cells. *EMBO J*. 1999;18:3964–3972.
20. Ii M, Nishimura H, Iwakura A, Wecker A, Eaton E, Asahara T, Losordo DW. Endothelial progenitor cells are rapidly recruited to myocardium and mediate protective effect of ischemic preconditioning via "imported" nitric oxide synthase activity. *Circulation*. 2005;111:1114–1120.
21. Pipili-Synetos E, Sakkoula E, Maragoudakis ME. Nitric oxide is involved in the regulation of angiogenesis. *Br J Pharmacol*. 1993;108:855–857.
22. Ziche M, Morbidelli L, Masini E, Granger HJ, Gepetti P, Ledda F. Nitric oxide promotes DNA synthesis and cyclic GMP formation in endothelial cells from post capillary venules. *Biochem Biophys Res Commun*. 1993;192:1198–1203.
23. Ziche M, Morbidelli L, Masini E, Amerini S, Granger HJ, Maggi CA, Geppetti P, Ledda F. Nitric oxide mediates angiogenesis *in vivo* and endothelial cell growth and migration *in vitro* promoted by substance P. *J Clin Invest*. 1994;94:2036–2044.
24. Aicher A, Heeschen C, Mildner-Rihm C, Urbich C, Ihling C, Technau-Ihling K, Zeiher AM, Dimmeler S. Essential role of endothelial nitric oxide synthase for mobilization of stem and progenitor cells. *Nat Med*. 2003;9:1370–1376.
25. Steinberg M, Silva M. Plerixafor: a chemokine receptor-4 antagonist for mobilization of hematopoietic stem cells for transplantation after high-dose chemotherapy for non-Hodgkin's lymphoma or multiple myeloma. *Clin Ther*. 2010;32:821–843.
26. Heissig B, Hattori K, Dias S, Friedrich M, Ferris B, Hackett NR, Crystal RG, Besmer P, Lyden D, Moore MA, Werb Z, Rafii S. Recruitment of stem and progenitor cells from the bone marrow niche requires MMP-9 mediated release of Kit-ligand. *Cell*. 2002;109:625–637.
27. Jones SP, Girod WG, Palazzo AJ, Granger DN, Grisham MB, Jourd'Heuil D, Huang PL, Lefer DJ. Myocardial ischemia-reperfusion injury is exacerbated in absence of endothelial cell nitric oxide synthase. *Am J Physiol*. 1999;276:H1567–H1573.
28. Razavi HM, Hamilton JA, Feng Q. Modulation of apoptosis by nitric oxide: implications in myocardial ischemia and heart failure. *Pharmacol Ther*. 2005;106:147–162.
29. Mees B, Recalde A, Loinard C, Tempel D, Godinho M, Vilar J, van Haperen R, Levy B, de Crom R, Silvestre JS. Endothelial nitric oxide synthase overexpression restores the efficiency of bone marrow mononuclear cell-based therapy. *Am J Pathol*. 2011;178:55–60.
30. Chen LL, Yin H, Huang J. Inhibition of TGF-beta1 signaling by eNOS gene transfer improves ventricular remodeling after myocardial infarction through angiogenesis and reduction of apoptosis. *Cardiovasc Pathol*. 2007;16:221–230.
31. Zhang D, Fan GC, Zhou X, Zhao T, Pasha Z, Xu M, Zhu Y, Ashraf M, Wang Y. Over-expression of CXCR4 on mesenchymal stem cells augments myoangiogenesis in the infarcted myocardium. *J Mol Cell Cardiol*. 2008;44:281–292.
32. Morimoto H, Takahashi M, Shiba Y, Izawa A, Ise H, Hongo M, Hatake K, Motoyoshi K, Ikeda U. Bone marrow-derived CXCR4+ cells mobilized by macrophage colony-stimulating factor participate in the reduction of infarct area and improvement of cardiac remodeling after myocardial infarction in mice. *Am J Pathol*. 2007;171:755–766.
33. Li J, Brown LF, Hibberd MG, Grossman JD, Morgan JP, Simons M. VEGF, flk-1, and flt-1 expression in a rat myocardial infarction model of angiogenesis. *Am J Physiol*. 1996;270:H1803–1811.

Downloaded from http://ahajournals.org by on April 30, 2021

34. Cho HJ, Lee N, Lee JY, Choi YJ, Ii M, Wecker A, Jeong JO, Curry C, Qin G, Yoon YS. Role of host tissues for sustained humoral effects after endothelial progenitor cell transplantation into the ischemic heart. *J Exp Med*. 2007;204:3257–3269.
35. Kawamoto A, Gwon HC, Iwaguro H, Yamaguchi JI, Uchida S, Masuda H, Silver M, Ma H, Kearney M, Isner JM, Asahara T. Therapeutic potential of ex vivo expanded endothelial progenitor cells for myocardial ischemia. *Circulation*. 2001;103:634–637.
36. Chavakis E, Aicher A, Heeschen C, Sasaki K, Kaiser R, El Makhfi N, Urbich C, Peters T, Scharffetter-Kochanek K, Zeiher AM, Chavakis T, Dimmeler S. Role of beta2-integrins for homing and neovascularization capacity of endothelial progenitor cells. *J Exp Med*. 2005;201:63–72.

CLINICAL PERSPECTIVE

Numerous clinical trials with cell therapy focusing on cardiac functional recovery after cardiovascular diseases, including myocardial infarction, have been performed over the past decade. Despite a certain extent of favorable outcome by the evolutional compared with conventional therapies, the trials have been required to overcome ethical, technical, and medical expense issues that may hinder the development of a novel therapeutic strategy. We have shown here that single administration of the CXC-chemokine receptor 4 antagonist AMD3100 exhibited sufficient therapeutic effect on cardiac functional recovery via mobilizing bone marrow–derived endogenous progenitor cells, including endothelial progenitor cells, by an endothelial nitric oxide synthase–dependent mechanism in a mouse ischemia/reperfusion injury model. The easy-to-handle, low-invasiveness, and inexpensive therapy with AMD3100 that we proposed in the present study can avoid the above-described hurdles to be cleared in clinical trials for cell therapy. AMD3100 treatment may not be able to restore cardiac function after myocardial infarction completely but could be a potent supplemental option after the established coronary recanalization/reperfusion technique with percutaneous transluminal coronary angioplasty balloons and stents. Clinical trials of autologous stem/progenitor cell therapy are ongoing, and positive outcomes have emerged, specifically in nonoption patients suffering from severe cardiovascular disease. Our data suggest that AMD3100, an endogenous stem/progenitor cell mobilizer, has the potential to be a simple but promising additional therapy, taking the place of stem/progenitor cell transplantation therapy for ischemic heart diseases.

Downloaded from http://ahajournals.org by on April 30, 2021
