# Supplementary material for: Cellular therapy for myocardial ischemia using a temperature-responsive biodegradable injectable polymer system with adipose-derived stem cells
Source: Sci Technol Adv Mater. 2021 Aug 6;22(1):627–42. doi: 10.1080/14686996.2021.1938212 (PMC8354160; doi:10.1080/14686996.2021.1938212)
Supplement: Supplemental Material [file TSTA_A_1938212_SM1513.pdf]

Supporting information

Cellular therapy for myocardial ischemia using a temperature-responsive biodegradable injectable polymer system with adipose-derived stem cells

Yuta Yoshizaki[1†], Hiroki Takai[2], Nozomi Mayumi[2], Soichiro Fujiwara[2], Akinori Kuzuya[2,3], Yuichi Ohya*[2,3]

[1] Organization for Research and Development of Innovative Science and Technology (ORDIST), Kansai University, 3–3–35 Yamate, Suita, Osaka 564–8680, Japan

[2] Faculty of Chemistry, Materials, Bioengineering, Kansai University, 3–3–35 Yamate, Suita, Osaka 564–8680, Japan

[3] Kansai University Medical Polymer Research Center (KUMP-RC), Kansai University, 3–3–35 Yamate, Suita, Osaka 564–8680, Japan

INDEX

Scheme S1. Synthesis of PCGA-*b*-PEG-*b*-PCGA (tri-PCG) by ring-opening polymerization.

Scheme S2. Synthesis of acryl capped-PCGA-PEG-PCGA (tri-PCG-Acryl).

Figure S1. Flow cytometric analysis for AdSCs isolated from C57BL/6N mice.

Figure S2. Illustration of a vertical flat plate-type culture vessel.

Figure S3. Results of rheological measurements for IP(A16) and IP(A25).

Figure S4. Proliferation of AdSCs cultured in IP hydrogels with medium ADSC-1 (containing 5% FBS) at 37°C for 7days.

Figure S5. Proliferation of AdSCs cultured on TCPS in medium ADSC-1 (containing 5% FBS) at 37°C for 4 days.

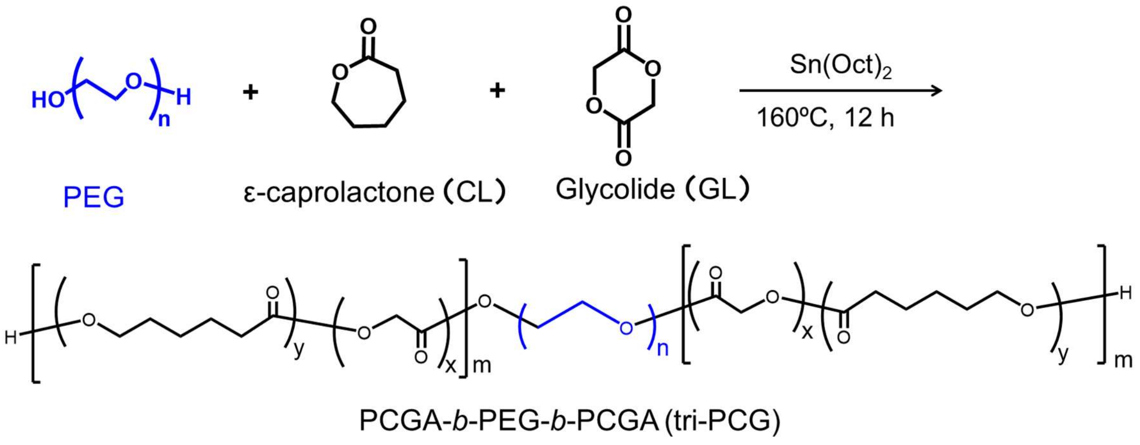

Scheme S1. Synthesis of PCGA-*b*-PEG-*b*-PCGA (tri-PCG) by ring-opening polymerization.

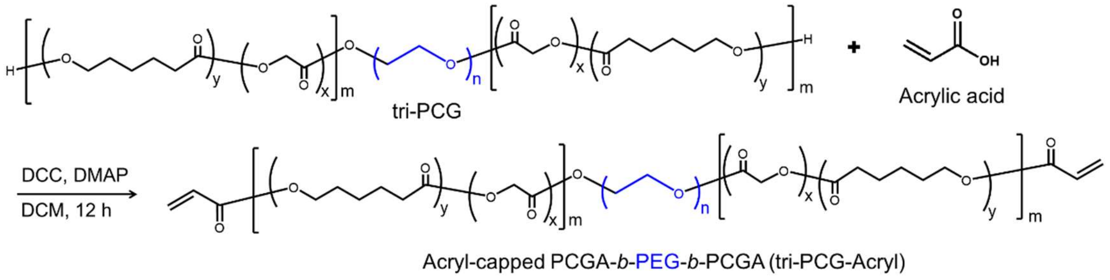

Scheme S2. Synthesis of acryl capped-PCGA-PEG-PCGA (tri-PCG-Acryl).

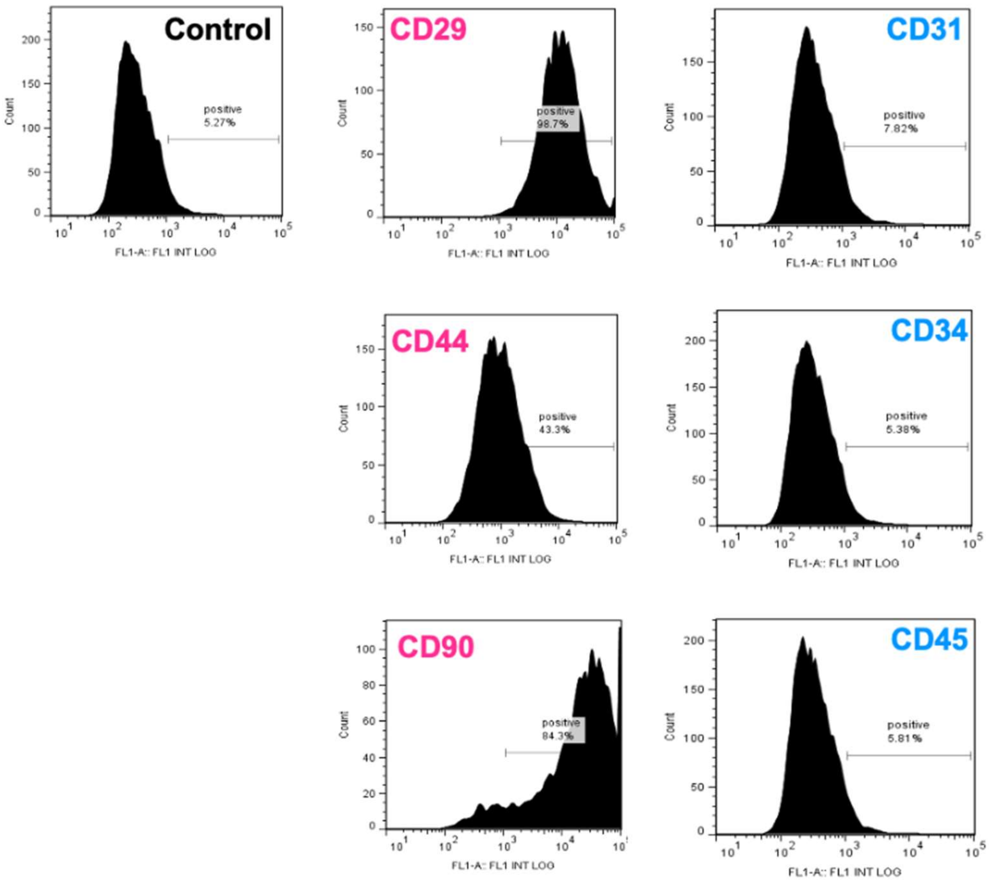

Figure S1. Flow cytometric analysis for AdSCs isolated from C57BL/6N mice [36]. CD29, CD44, and CD90 were used as positive makers, and CD31, CD34, and CD45 was used as negative makers.

FITC anti-mouse CD31 Antibody, FITC anti-mouse CD45 Antibody, FITC anti-mouse CD90.2 Antibody, FITC anti-mouse/human CD44 Antibody, FITC anti-mouse/rat CD29 Antibody, and PE anti-mouse CD34 Antibody were purchased from BioLegend (San Diego, USA). The suspension of AdSCs containing 2 × 10^5 cells/500 µL was centrifuged (400g, 4°C, 5 min). After removal of supernatant, the solution containing antibody (100 µL) was added and incubated for 30 min with ice-cooling. After washing with PBS twice, PBS (500 µL) was added and performed flowcytometic analysis (Gallios, Beckman Coulter, Inc., Brea, CA, USA). The amounts of antibody used were 1.0 µg/10^6 cells for anti-CD31 and anti-CD34, and 0.25 µg/10^6 cells for other antibodies. The results shows the isolated cells had high content of mesenchymal stem cells.

References

[36] Nagata H, Ii M, Kohbayashi E, et al. Cardiac adipose-derived stem cells exhibit high differentiation potential to cardiovascular cells in C57BL/6 Mice. Stem Cells Transl Med. 2016; 5: 141-151.
[45] Sun M, Wang S, Li Y, et al. Adipose-derived stem cells improved mouse ovary function after chemotherapy-induced ovary failure. Stem Cell Res Ther. 2013; 4: 80.
https://resources.rndsystems.com/images/site/wp-msc-13763.pdf

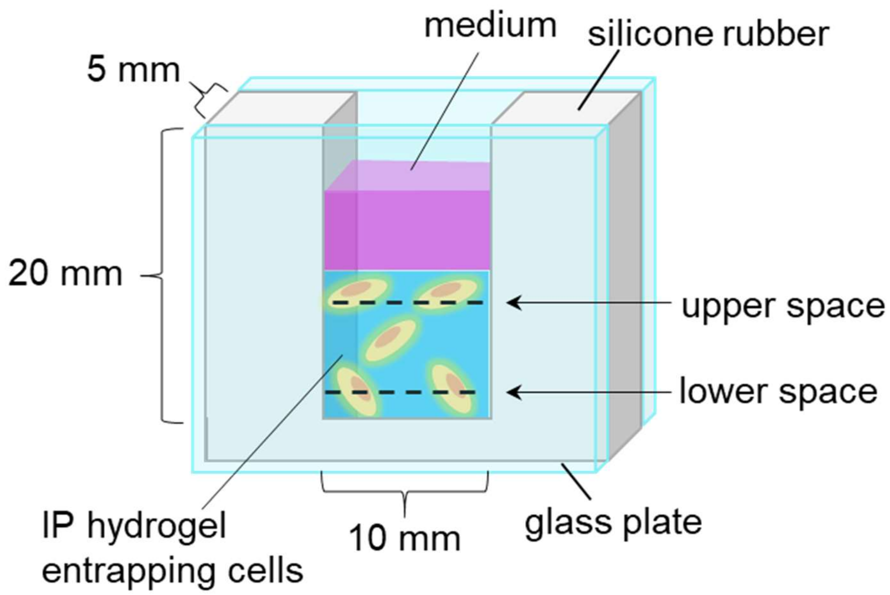

Figure S2. Illustration of a vertical flat plate-type culture vessel. The silicone rubber was sandwiched with slide glasses and IP hydrogels containing AdSCs were prepared in the hollow place.

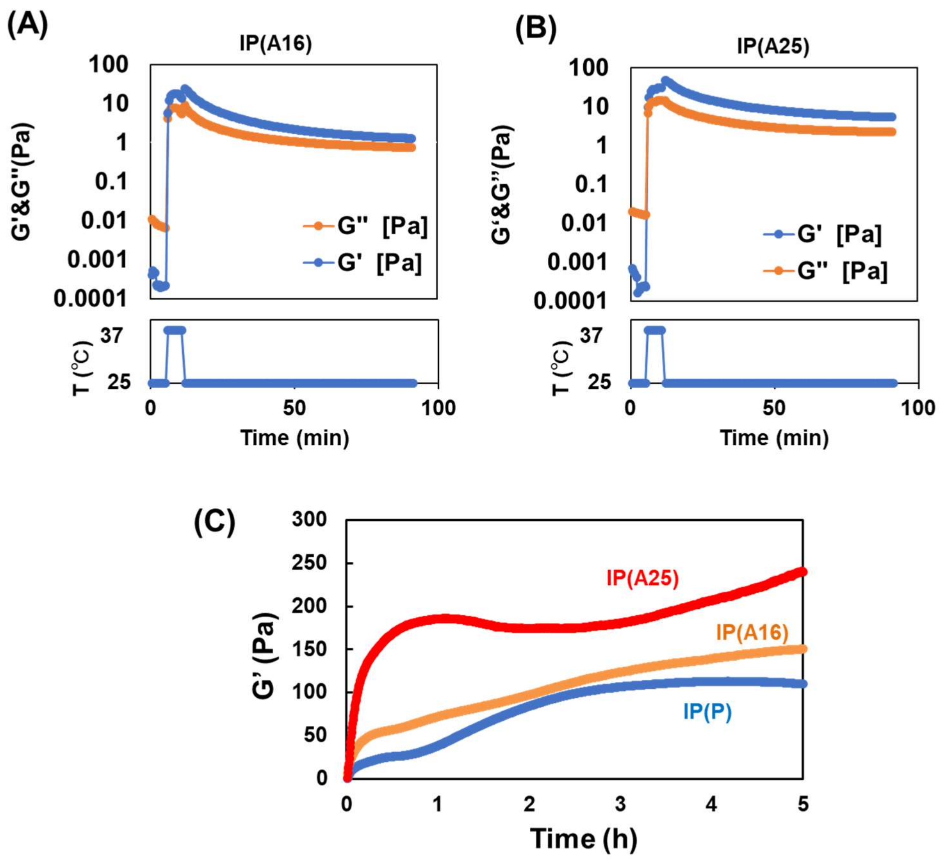

Figure S3. Results of rheological measurements for IP(A16) and IP(A25).

(A)(B) Time course of storage (*G*', blue) and loss moduli (*G*", orange) for IP(A16) (A) and IP(A25) (B) upon heating to 37 °C for 5 min and subsequent cooling to 25 °C. Total copolymer concentration = 15 wt%. Temperature change schedule is shown at the bottom of each figure.

(C) Time course of storage moduli (*G'*) of IP(P) (blue), IP(A16) (orange) and IP(A25) (red) after heating to 37°C and further maintaining at 37°C. Total polymer concentration = 15 wt%.

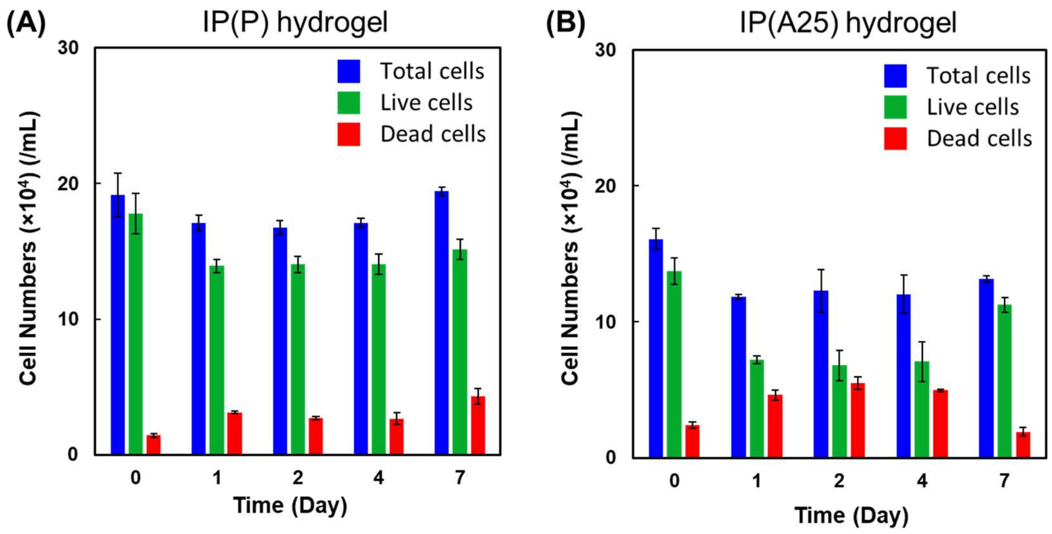

Figure S4. Proliferation of AdSCs cultured in IP hydrogels with medium ADSC-1 (containing 5% FBS) at 37°C for 7days. (A) AdSCs in IP(P) hydrogel, (B) AdSCs in IP(A25) hydrogel. Blue, green and red bars represent number of total cells, number of live cells, and number of dead cells, respectively. Cells (2×10^4) were mixed with IP formulations at day 0 and incubated in IP hydrogels sunk in the serum-containing medium (ADSC-2). Polymer concentration was 15wt%.

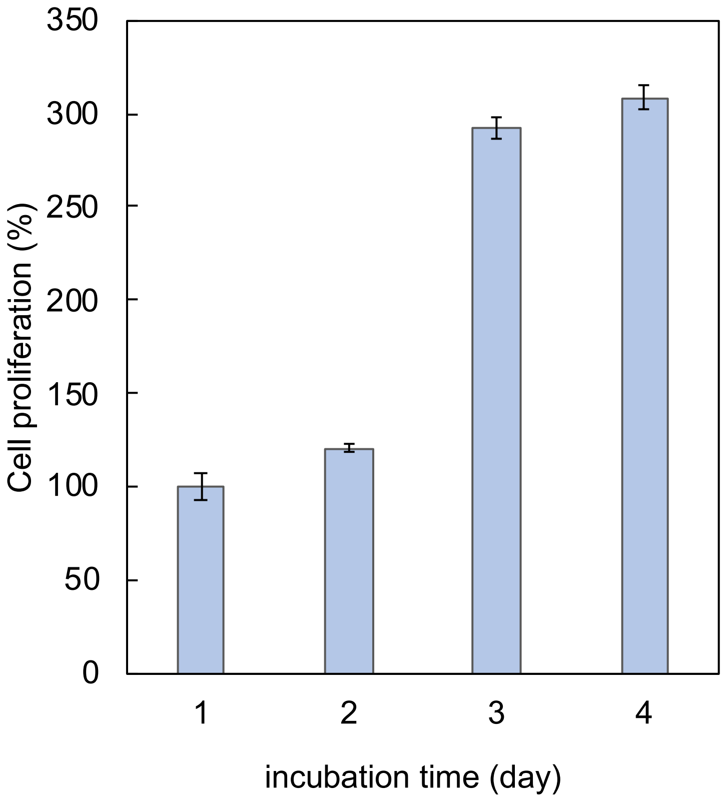

Figure S5. Proliferation of AdSCs cultured on TCPS in medium ADSC-1 (containing 5% FBS) at 37°C for 4 days.

The number of the cells were estimated by WST-8 assay. WST-8 reagent was obtained from Dojindo Molecular Technologies (Tokyo, Japan). Cell Counting Kit-8 (WST-8 reagent) was purchased from DOJINDO LABORATORIES Co., Ltd. (Kumamoto, Japan). The absorbance of 96-well plate for cytotoxicity assay were measured by iMark microplate reader (Bio-Rad Laboratories, Inc., Hercules, CA, USA).
